# Serum starvation drives ALIX-dependent extracellular vesicle biogenesis and determines tumor progression

**DOI:** 10.1172/jci.insight.197924

**Published:** 2026-06-08

**Authors:** Xueqiang Peng, Jiaxing Liu, Guolong Zeng, Yafei Xiao, Zhixiong Hao, Guangpeng He, Hongyuan Jin, Yu Gao, Shilei Tang, Shibo Wei, Yan Li, Yifan Yu, Liang Yang, Hangyu Li

**Affiliations:** 1 Department of General Surgery, The Fourth Affiliated Hospital, China Medical University, Shenyang, Liaoning, China.; 2 Shenyang Key Laboratory for Biomedical and Intelligent Mesh, Shenyang, Liaoning, China.; 3 Shenyang Clinical Medical Research Center for Diagnosis, Treatment and Health Management of Early Digestive Cancer, Shenyang, Liaoning, China.; 4 Liaoning Province-Zimbabwe Belt and Road Joint Laboratory on Biomedical Data Sharing and Cancer Prevention and Control, Jinzhou, Liaoning, China.; 5 The First Affiliated Hospital of Anhui Medical University, Hefei, Anhui, China.; 6 Immunology Frontier Research Center, The University of Osaka, Osaka, Japan.; 7 Department of General Surgery, The First Affiliated Hospital of Jinzhou Medical University, Jinzhou, Liaoning, China.

**Keywords:** Cell biology, Oncology, Cancer

## Abstract

Tumor cells are constantly confronted with nutrient deprivation; however, the effect of serum starvation on the remodeling of endosomal compartments and extracellular vesicles (EVs) in tumor cells remains unclear. Here, we found that serum starvation pronouncedly promotes multivesicular body (MVB) biogenesis, EV formation, and cargo selection. Specifically, by generating a constitutively active Rab5Q79L mutant to induce the enlargement of MVB, we revealed for the first time to our knowledge that ANXA3 is sorted into intraluminal vesicles (ILVs) of MVB. Mechanistically, we confirmed that serum starvation regulates the endosomal sorting complex required for transport–associated (ESCRT-associated) protein ALG-2 interacting protein X (ALIX), which recruits ESCRT-III to MVB and binds to annexin A3 (ANXA3) to mediate its sorting into ILVs of MVB. Our study highlights that serum starvation promotes an ALIX-dependent ESCRT-III recruitment pathway, which loads protumor ANXA3 cargo to exert a profound effect on tumor progression.

## Abstract 2

<strong>Serum Starvation Drives Extracellular Vesicle Biogenesis and Determines Tumor Progression</strong>

## Graphical abstract

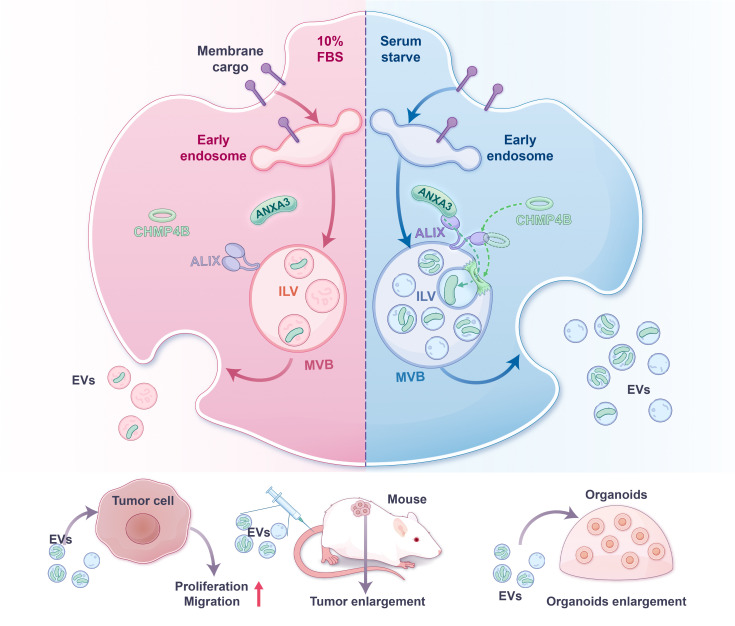

## Introduction

Tumor cells are constantly exposed to various stressful microenvironments, and they can adapt to their energetic and biosynthetic needs by reprogramming metabolic processes, thereby maintaining cell proliferation and survival under different stress conditions (1–3). Due to uncontrolled proliferative behavior, cancer cells are frequently situated in microenvironments characterized by hypoxia and limited serum component availability (4). Serum starvation, one of the most common environmental stresses encountered by tumor cells, has also been widely used to investigate adaptive survival mechanisms, such as the acquisition of antiapoptotic responses in cancer cells (2, 5). Under starvation stress, tumor cells initiate a series of vital processes that contribute to oncogenesis and resistance to cell death, such as activation of c-JNK signaling cascades, modulation of mTOR activity, and alterations in the endomembrane system (2, 6). Cancer cells activate intrinsic antiapoptotic mechanisms and secrete extracellular signals, including soluble bioactive molecules and extracellular vesicles (EVs), which mediate cellular communication, thereby influencing tumor initiation, progression, and patient prognosis (7–9).

Previously, we have described in detail the biogenesis mechanisms of multivesicular bodies (MVB) and EVs (10, 11). EVs are lipid bilayer-enclosed vesicles containing proteins, nucleic acids, lipids, and metabolites acting as important messengers to the recipient cells. (10–12). EVs are defined and classified based on their size, biological origin, and cargo; apoptotic bodies are larger than 1,000 nm and contain histones and DNA; microvesicles (MVs) range from 100 to 1,000 nm in diameter and are mainly budding from the plasma membrane; exosomes are 30–200 nm in diameter and originate from the secretion of intraluminal vesicles (ILVs) into the microenvironment following the fusion of MVB with the plasma membrane (10, 13). Under stresses, including external environmental factors or genetic mutations, tumor cells can secrete heterogeneous EVs, which extensively involved in remodeling the tumor microenvironment and are essential in promoting tumor growth, metastasis, and immune evasion (9, 14–16). This correlation between cellular stress and the composition of EVs suggests the existence of precise cargo-sorting mechanisms. A well-established model proposes that membrane or cytosolic cargoes targeted for secretion are sorted into ILVs within MVB via either endosomal sorting complex required for transport–dependent (ESCRT-dependent) or ESCRT-independent pathways (9, 10, 12, 17). The ESCRT machinery consists of 4 distinct ESCRT complexes and associated proteins, such as ALG-2 interacting protein X (ALIX) and vacuolar protein sorting gene 4 (VPS4), which function sequentially to mediate the sorting of cargoes into ILVs of MVB. Specifically, cargo is initially recognized and captured by ESCRT-0, followed by ESCRT-I and ESCRT-II, which drive the membrane invagination and budding at the site of the ESCRT-0 cargo complex (18). Subsequently, ESCRT-III subunits mediate the scission of the bud, enabling the ILV formation from the MVB limiting membrane (9, 17). ESCRT-independent sorting mechanisms include ceramide-dependent pathways and sorting via tetraspanins such as CD63 (18, 19). These distinct mechanisms can act cooperatively or independently to generate different MVB subpopulations. For example, Kilinc et al. demonstrated that oncogenic mutations such as *MYC* and *AURKB* alter the composition of EVs through ESCRT pathway components TSG101 and ALIX, which in turn pronouncedly promote cell malignant biological behaviors (9). Wang et al. found that MFGE8 promotes ESCRT-dependent PD-L1 sorting, triggering the secretion of PD-L1^+^ EVs and thereby enhancing immunotherapy resistance (17).

Nevertheless, serum starvation has been shown to affect EV protein composition and secretion (5, 8, 14, 16). However, the biogenesis and cargo sorting mechanisms of MVB under serum starvation remain largely unexplored. Investigating the biogenesis and cargo sorting of MVB under serum starvation will enhance our understanding of cellular stress homeostasis, elucidate the intrinsic factors contributing to EV heterogeneity, and provide a foundation for the targeted manipulation of EV secretion and function, ultimately advancing translational research in targeted therapy and drug delivery. In this study, we used superresolution immunofluorescence (IF) microscopy and EM to reveal that serum starvation increases MVB formation and EV secretion in tumor cells. Notably, we found that, during this process, ALIX recognizes ANXA3 cargo and recruits CHMP4B (ESCRT-III) to the MVB membrane, facilitating the sorting of ANXA3 into ILVs, which are subsequently secreted as ANXA3^+^ EVs. Finally, through in vitro cell-based assays, organoid models, and mouse s.c. tumor experiments, we confirmed that serum starvation–induced ANXA3^+^ EVs promote the progression of tumors. These findings indicate that serum starvation can pronouncedly alter MVB biogenesis and, through aggressive EV secretion, can have profound effects on recipient cells.

## Results

### Serum starvation induces ILV budding and MVB biogenesis.

Due to their unregulated growth patterns, tumor cells often reside in microenvironments with severe hypoxia and limited serum component supply at later stages. Prolonged exposure to such conditions frequently leads to the evolution of antiapoptotic capabilities in these tumor cells (4). Tumor cells cultured in vitro exhibit marked alterations in metabolic activity following serum starvation. When stress persists, these cells cease proliferative activity and activate stress response pathways, such as autophagy and endocytosis, to maintain cellular homeostasis (1, 4, 20). At the subcellular level, tumor cells, under nutrient-rich conditions, exhibit well-organized internal structures and intact organelles, such as mitochondria, with rare occurrences of autophagosomes and MVB. However, when exposed to environmental stress for several minutes up to 4 hours, tumor cells rapidly activate processes such as microautophagy, macroautophagy, and the formation of MVB to meet cellular energy demands and sustain survival (1, 21).

**Figure 1:**
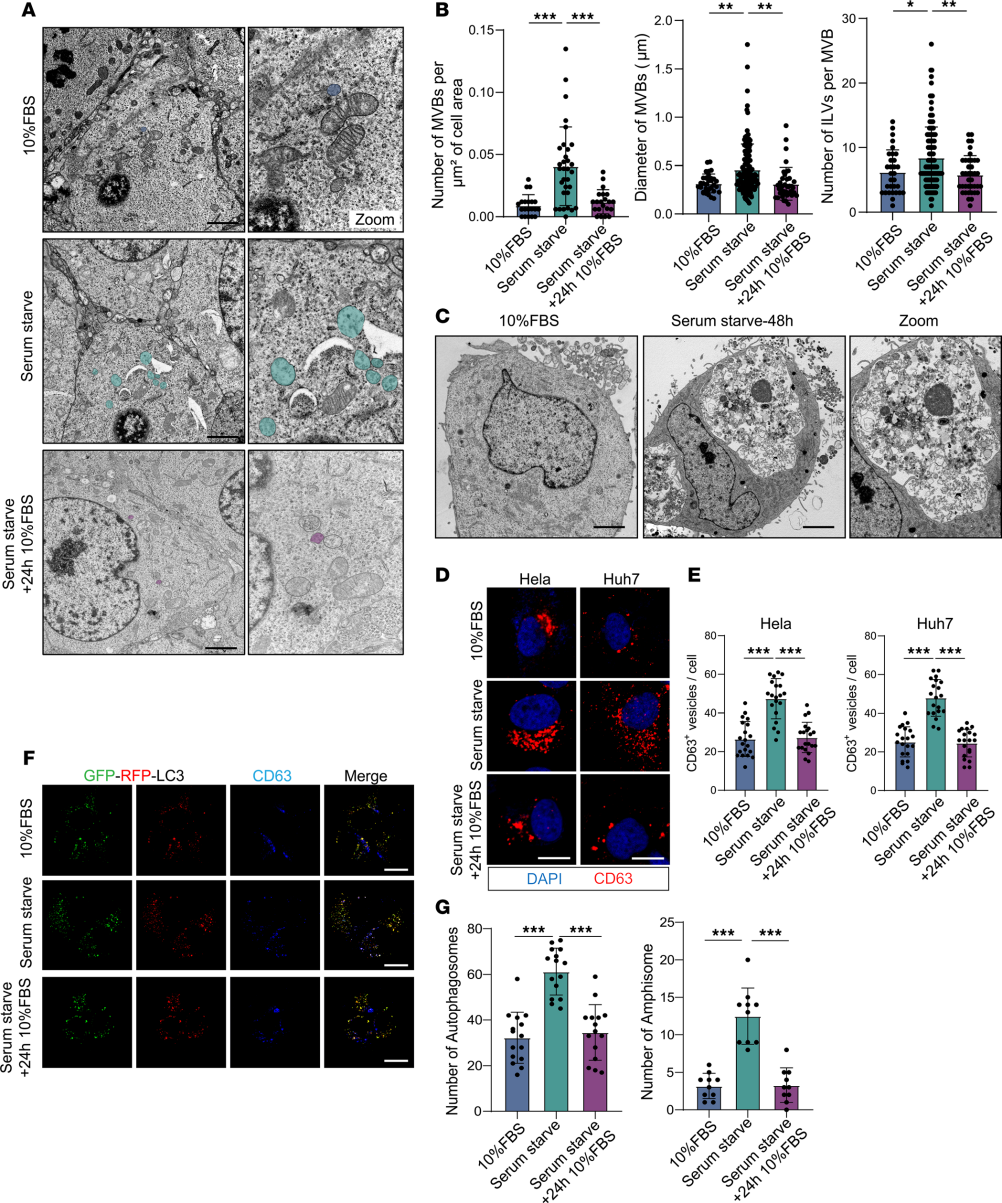
Serum starvation induces ILV budding and MVB biogenesis in tumor cells. (**A**) TEM of cells cultured with 10% FBS (top), under serum starvation (middle), or recultured with 10% FBS for 24 hours after starvation (bottom). MVB are pseudocolored. Scale bar: 2 μm. Enlarged views are shown at right. (**B**) Quantification of MVB number per μm^2^ of cell area (left), MVB diameter (middle), and ILV number per MVB (right) under the indicated conditions. *n* = 20, 32, and 21 fields (left) and 31, 128, and 39 MVB (middle and right). (**C**) TEM of cells cultured with 10% FBS or subjected to 48 hours serum starvation. Scale bar: 2 μm. Enlarged views are shown at right. (**D**) Confocal images of CD63^+^ vesicles in cells cultured with 10% FBS, under serum starvation, or recultured with 10% FBS after starvation. Nuclei were stained with DAPI. Scale bar: 10 μm. (**E**) Quantification of CD63^+^ vesicles in **D**. *n* = 20 cells. (**F**) Polar-SIM images showing CD63 (blue) colocalization in cells stably expressing tandem fluorescent LC3 under the indicated conditions. LC3 (yellow) marks autophagosomes; LC3/CD63 colocalization (white) indicates amphisomes. Scale bar: 10 μm. (**G**) Quantification of autophagosomes (left) and amphisomes (right) in **F**. *n* = 15 cells (left) and 10 cells (right). Data are presented as mean ± SD. Statistical analysis was performed using ordinary 1-way ANOVA followed by Tukey’s multiple-comparison test. *P < 0.05, **P < 0.01, ***P < 0.001.

To investigate the effect of serum starvation on organelle dynamics in tumor cells, we established a starvation stress model in tumor cell lines. Transmission electron microscopy (TEM) was employed to investigate the ultrastructural changes in HeLa cells. Under nutrient-rich conditions, HeLa cells displayed well-defined endoplasmic reticulum (ER) and mitochondrial structures. In contrast, after 24 hours of serum starvation, there was a marked increase in the number of MVB, a marked enlargement of MVB diameter, and an increased number of ILVs (Figure 1, A and B). To determine whether this stress-induced effect is reversible, we restored nutrient supply to the previously starved cells for 24 hours. The results showed a marked reduction in the number of MVB and a decrease in their diameter and ILV content, indicating a reestablishment of cellular structural homeostasis (Figure 1, A and B). However, when the stress duration was extended to 48 hours or longer, large autolysosomes (2–4 μm in diameter) were observed in tumor cells that had not undergone apoptosis. These large autolysosomes filled the cytoplasm, resulting in nuclear condensation (Figure 1C). TEM analysis indicated that these large autolysosomes likely originate from the fusion of multiple autophagolysosomes. Moreover, contact sites and fusion events between these large autolysosomes and the plasma membrane were observed, leading to the release of membrane vesicles into the extracellular space, consistent with previous reports (21).

Furthermore, confocal microscopy was used to examine changes in CD63^+^ MVB in tumor cells following starvation treatment. Consistent with the TEM results, starvation led to a marked increase in the number of MVB (Figure 1, D and E), suggesting that cells substantially upregulate intracellular vesicle formation in response to microenvironmental stress, thereby adapting to environmental changes (21, 22). Upon restoration of nutrient supply for 24 hours, the number of MVB returned to baseline levels (Figure 1, D and E). It is well established that external environmental cues regulate the fate of different MVB subtypes within the cell. MVB can either fuse with lysosomal compartments for degradation of their contents or be transported to the cell periphery, where they fuse with the plasma membrane to secrete EVs (10, 14, 23). To further elucidate the distribution of MVB under starvation stress, we performed confocal microscopy on HeLa cells transfected with a dual-fluorescent LC3 construct (GFP-RFP-LC3) and examined the colocalization with endogenous CD63. The results showed an increased number of white fluorescent puncta, indicating the colocalization of GFP-RFP-LC3 and CD63, confirming that starvation induces the formation of amphisomes. Notably, after 24 hours of nutrient restoration, the number of amphisomes was pronouncedly reduced (Figure 1, F and G), suggesting that starvation stress promotes organelle fusion events within the cell. These results demonstrate that serum starvation enhances ILV budding and MVB biogenesis in tumor cells.

### Serum starvation increases EV secretion.

Studies have revealed that serum starvation can affect the heterogeneity of EVs (5, 8, 24). However, the underlying mechanisms of EV biogenesis under serum starvation and their effect on the tumor microenvironment remain unclear. In addition to altering intracellular metabolism and homeostatic mechanisms, serum starvation may also affect the quality and composition of EVs, thereby exerting critical downstream effects on tumor biology (21, 24). To better investigate the differences in the quality and composition of stress-induced EVs from tumor cells, it is essential to ensure that the tumor cells are maintained in a state of stress equilibrium. Excessive apoptosis or necrosis would lead to the release of cell debris and nonsecretory vesicles, which could interfere with the functional analysis of sEVs (16, 21). Therefore, we first performed CCK8 and apoptosis assays to establish a time course, and we selected a treatment window in which the proportion of apoptotic HeLa, Huh7, and HCT116 cells remained below 5% as the optimal period for EV induction (Supplemental Figure 1, A–D; supplemental material available online with this article; https://doi.org/10.1172/jci.insight.197924DS1) (5). Both CCK8 and flow cytometry–based apoptosis assays consistently indicated that apoptosis levels exceeded 5% at 48 hours, whereas a 24-hour induction period was appropriate for maintaining cell viability and minimizing apoptotic interference (Supplemental Figure 1, A–D).

**Figure 2:**
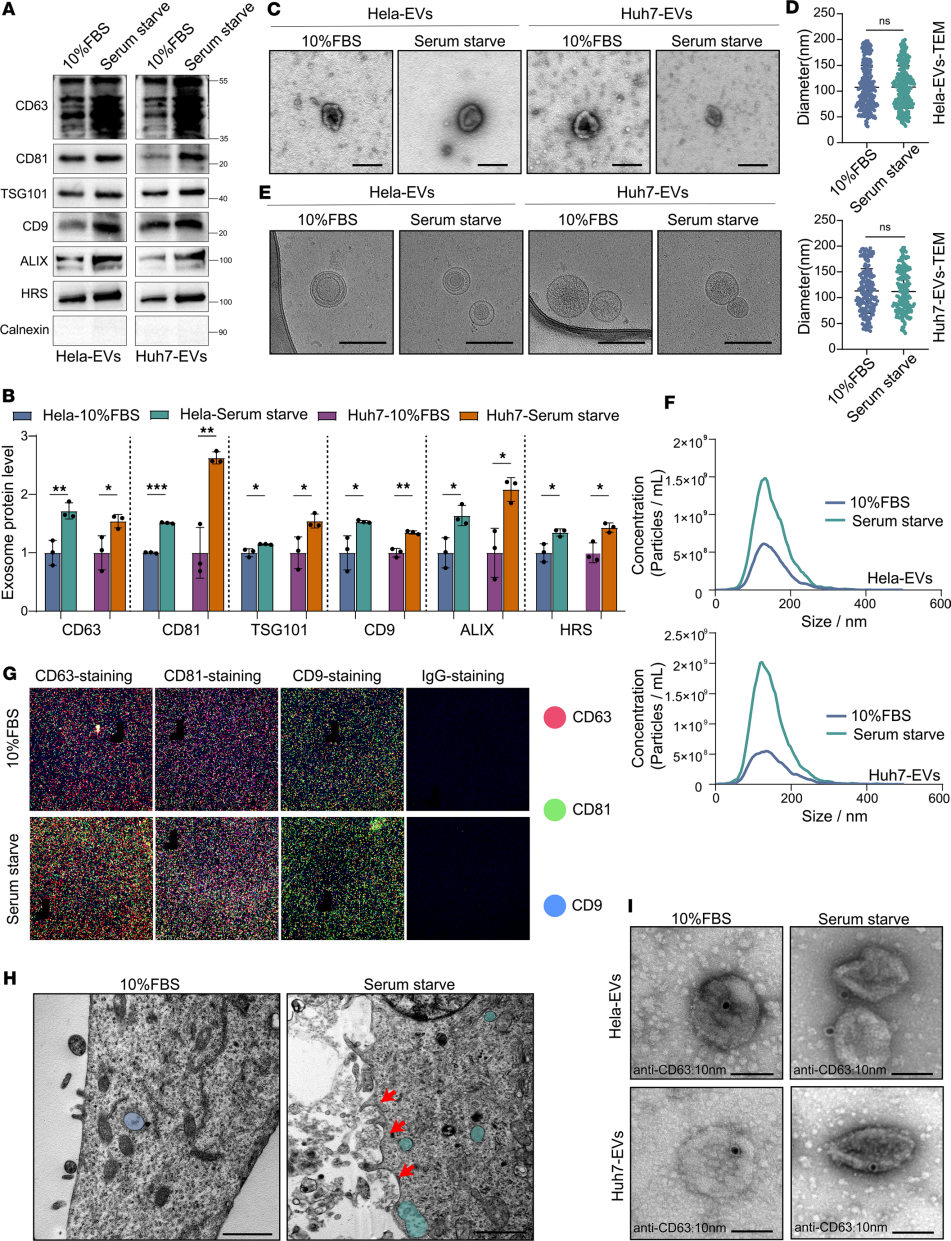
Serum starvation induces EVs secretion. (**A**) Western blot analysis of EV markers in HeLa-EVs and Huh7-EVs under 10% FBS or serum starvation. CD63, CD81, TSG101, CD9, ALIX, and HRS were used as EV markers; calnexin was used to assess cellular contamination. (**B**) Quantification of EV marker expression in HeLa-EVs and Huh7-EVs. *n* = 3. (**C**) TEM images of HeLa-EVs and Huh7-EVs under 10% FBS or serum starvation. Scale bar: 200 nm. (**D**) TEM-based diameter distribution of HeLa-EVs (top) and Huh7-EVs (bottom). *n* = 259 EVs (top) and 177 EVs (bottom). (**E**) Cryo-EM images of HeLa-EVs and Huh7-EVs. Scale bar: 200 nm. (**F**) NTA of HeLa-EVs (top) and Huh7-EVs (bottom) showing size distribution and concentration. (**G**) ExoView analysis of surface CD63 (red), CD81 (green), and CD9 (blue) on EVs under 10% FBS or serum starvation. IgG served as a negative control. (**H**) TEM of MVB formation in HeLa and Huh7 cells under 10% FBS or serum starvation. MVB are pseudocolored; red arrows indicate MVB-plasma membrane fusion. Scale bar: 1 μm. (**I**) IEM showing CD63 distribution on EVs using gold-labeled anti-CD63 antibody. Gold particles were mainly localized on EVs, indicating specific CD63 localization. Scale bar: 100 nm. Data are presented as mean ± SD. Comparisons between 2 groups were done with 2-tailed Student’s *t* test. *P < 0.05, **P < 0.01, ***P < 0.001.

To assess the secretion of EVs from tumor cells following starvation stress, we employed differential ultracentrifugation — a well-established technique for isolating EVs since there is currently no universally optimal method for absolute EV purification. Tumor cells (HeLa, Huh7, and HCT116) were subjected to serum starvation for 24 hours, and EVs were subsequently isolated from the corresponding conditioned media. Western blot analysis confirmed the presence of canonical sEV markers — including tetraspanins (CD63, CD81, CD9) and ESCRT-related proteins (HRS, ALIX, TSG101) — while the ER marker Calnexin was absent (Figure 2, A and B, and Supplemental Figure 2A) (13). These results indicate that serum starvation promotes the release of EVs (Figure 2, A and B, and Supplemental Figure 2A). TEM and cryo-electron microscopy (Cryo-EM) were used to morphologically characterize the isolated EVs, confirming their typical size and morphology of EVs (Figure 2, C and E). In addition, TEM analysis revealed no marked change in the diameter of EVs released from tumor cells following starvation treatment (Figure 2D). Nanoparticle tracking analysis (NTA) showed that the total particle number and the number of EVs (particles < 200 nm in size) derived from serum-starved tumor cells increased. Consistently, NTA results demonstrated no marked difference in the average size of EVs between the control and serum-starved groups (Figure 2F and Supplemental Figure 2, B–E).

Additionally, we utilized ExoView technology to characterize different EV subpopulations in HeLa cell supernatants. This approach enabled the detection of 3 single-positive EV populations (CD63^+^, CD81^+^, CD9^+^), 3 double-positive populations (CD63^+^CD81^+^, CD81^+^CD9^+^, CD63^+^CD9^+^), and 1 triple-positive population (CD63^+^CD81^+^CD9^+^), with protein expression profiles assessed under different microenvironmental conditions (Figure 2G). Importantly, TEM analysis of membrane structural changes in tumor cells under serum starvation revealed the presence of MVB and potential MVB–plasma membrane fusion and EV secretion events following 24 hours of starvation. These observations indicate that serum starvation stress drives MVB-dependent EV secretion (Figure 2H). This phenomenon has also been observed in the secretion of Wnt7a^+^ EVs, as recently reported by Gurriaran-Rodriguez et al. (25, 26). Furthermore, to confirm EV specificity, EVs isolated from HeLa and Huh7 cells were shown to be CD63^+^ (Figure 2I). In sum, these results indicate that serum starvation increases EV secretion in tumor cells.

### Proteomic characteristics of EVs induced by serum starvation.

**Figure 3:**
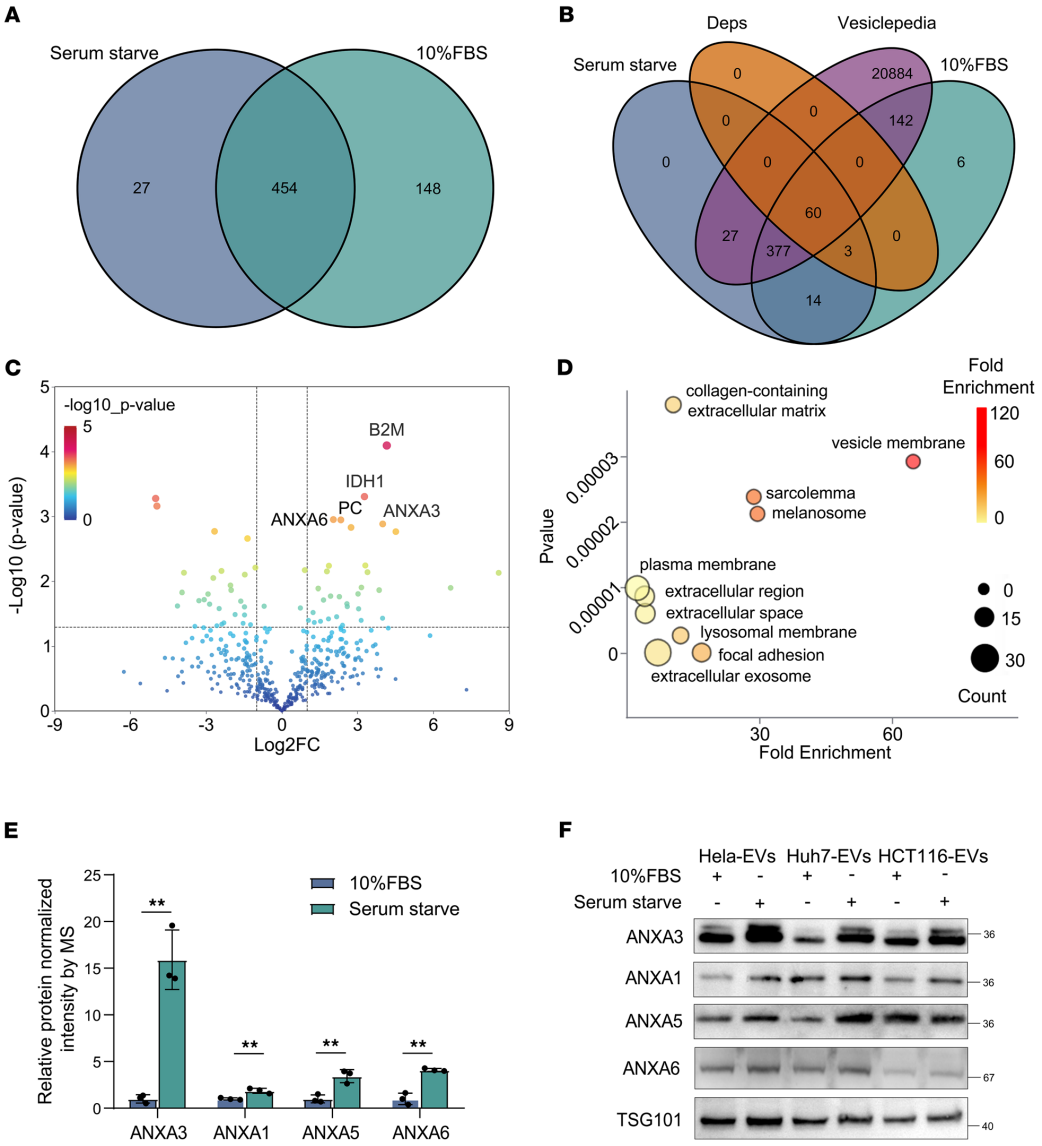
Proteomic characteristics of EVs induced by serum starvation. (**A**) Venn diagram comparing EV proteins identified under serum starvation and 10% FBS. Numbers indicate shared and unique proteins. (**B**) Venn diagram showing overlap among proteins from the serum starvation group, DEPs, the 10% FBS group, and Vesiclepedia. (**C**) Volcano plot of differential EV protein expression under serum starvation versus 10% FBS. (**D**) GO cellular component enrichment of DEPs shown as a bubble plot. Color indicates fold enrichment, bubble size indicates protein number, and the *y* axis shows –log_10_(*P* value). (**E**) Relative levels of ANXA3, ANXA1, ANXA5, and ANXA6 in HeLa EVs under 10% FBS or serum starvation based on quantitative proteomics. *n* = 3. (**F**) Western blot analysis of ANXA3, ANXA1, ANXA5, and ANXA6 in EVs from HeLa, Huh7, and HCT116 cells under 10% FBS or serum starvation. TSG101 served as an EV marker. Data are presented as mean ± SD. Comparisons between 2 groups were done with 2-tailed Student’s *t* test. **P < 0.01.

To precisely characterize the proteomic profile of EVs under starvation conditions, we employed a 5-dimensional (5D) label-free quantitative proteomics approach. Each sample was analyzed in 3 independent biological replicates. Proteins were considered positively identified if at least 2 unique peptides were detected in 2 or more experiments (14, 27). The results showed that 602 proteins were identified in EVs isolated from control HeLa cells, whereas 481 proteins were identified in EVs derived from serum-starved cells (Figure 3A). Protein expression analysis revealed that, compared with the control group, 63 proteins (13.1%) were pronouncedly altered in EVs under starvation conditions, with 29 proteins (6.0%) downregulated and 34 proteins (7.1%) upregulated. In addition, unique protein compositions were observed in each experimental group, indicating that EVs display pronounced cargo heterogeneity (Figure 3, B and C). We compared our EV proteomics data with the Vesiclepedia database and found that most proteins in our dataset (96.3%) had already been reported in other EV studies (Figure 3B). Although most proteins matched those in existing databases, our study identified several previously unreported proteins. For example, among the 17 proteins found in both the serum starvation and control groups, 3 proteins (CFAP97, TENM1, and CTSL) exhibited marked differences. Six proteins were detected exclusively in the control group — PHB1, SMAP, ATP5F1A, ATP5F1B, TUT4, and MDFIC2. Additionally, 27 proteins, although listed in vesicle databases, were detected only in the serum starvation group, suggesting that these proteins may be involved in specific biological processes. GO enrichment analysis revealed that these 27 proteins are mainly involved in biological pathways such as actin filament organization, apoptotic process, endothelial cell migration, and positive regulation of the epidermal growth factor receptor signaling pathway (Supplemental Table 1).

We further focused on the 34 pronouncedly upregulated differential proteins and performed GO enrichment analysis on this subset. The top 10 enriched pathways are ranked by significance (*P* value) and are presented in Figure 3D. These enriched GO terms are primarily associated with EVs and their membrane structures, including “extracellular exosome,” “extracellular region,” and “focal adhesion.” Notably, “vesicle membrane” exhibited the highest fold enrichment (Fold Enrichment = 64.67, *P* = 2.92 × 10^–5^, FDR < 0.01), suggesting that membrane-associated proteins of vesicles may undergo specific regulatory changes under starvation conditions. Using the WebGestalt database, we further constructed a network diagram of these top 10 enriched terms, highlighting the key role of vesicle membranes in linking vesicles and organelle membranes (Supplemental Figure 3A).

We further analyzed the 4 proteins involved in the “vesicle membrane” category — ANXA1, ANXA3, ANXA5, and ANXA6. Recent studies have demonstrated that the annexin family is critical in cell membrane repair, exosome secretion, and MV release (28, 29). Proteomic sequencing data reveal that ANXA1, ANXA3, ANXA5, and ANXA6 exhibited differential expression in the serum starvation group, with ANXA3 displaying the highest fold change and a pronouncedly greater upregulation compared with the other family members (Figure 3E). This finding was further validated by Western blot analysis of EVs derived from HeLa, Huh7, and HCT116 cells, confirming that ANXA3 protein is highly enriched in the vesicle membrane and likely plays a crucial functional role (Figure 3F and Supplemental Figure 3B). Meanwhile, GSEA revealed that ANXA3, a core vesicle membrane gene, exhibited the highest normalized enrichment score (Supplemental Figure 3C and Supplemental Table 2). ANXA3 is an important member of the annexin family of Ca^2+^-dependent phospholipid-binding proteins, also known as lipocortin III. It is pronouncedly upregulated in various types of tumors, including hepatocellular carcinoma, lung cancer, prostate cancer, and ovarian cancer. Additionally, ANXA3 is closely associated with the progression of cancer and inflammatory diseases (30–34). To further determine whether serum starvation–induced secretion of ANXA3^+^ EVs is tumor specific, tumor cells and their corresponding normal cell lines were subjected to serum starvation, followed by isolation of EVs and assessment of ANXA3^+^ EV levels. Western blot analysis showed that serum starvation pronouncedly increased ANXA3 protein levels in EVs derived from Huh7, HeLa, and HCT116 cells, whereas no pronounced changes were observed in EVs from the corresponding normal cell lines THLE-2, HcerEpic, and NCM460 (Supplemental Figure 3, D and E). These results indicate that serum starvation selectively promotes enrichment of ANXA3 protein in tumor cell–derived EVs, with no pronouncedly regulatory effect observed in normal cells. These findings suggest that ANXA3 may be involved in starvation-induced vesicle-associated biological changes in tumors.

### ANXA3 is enriched in starvation-induced EVs.

**Figure 4:**
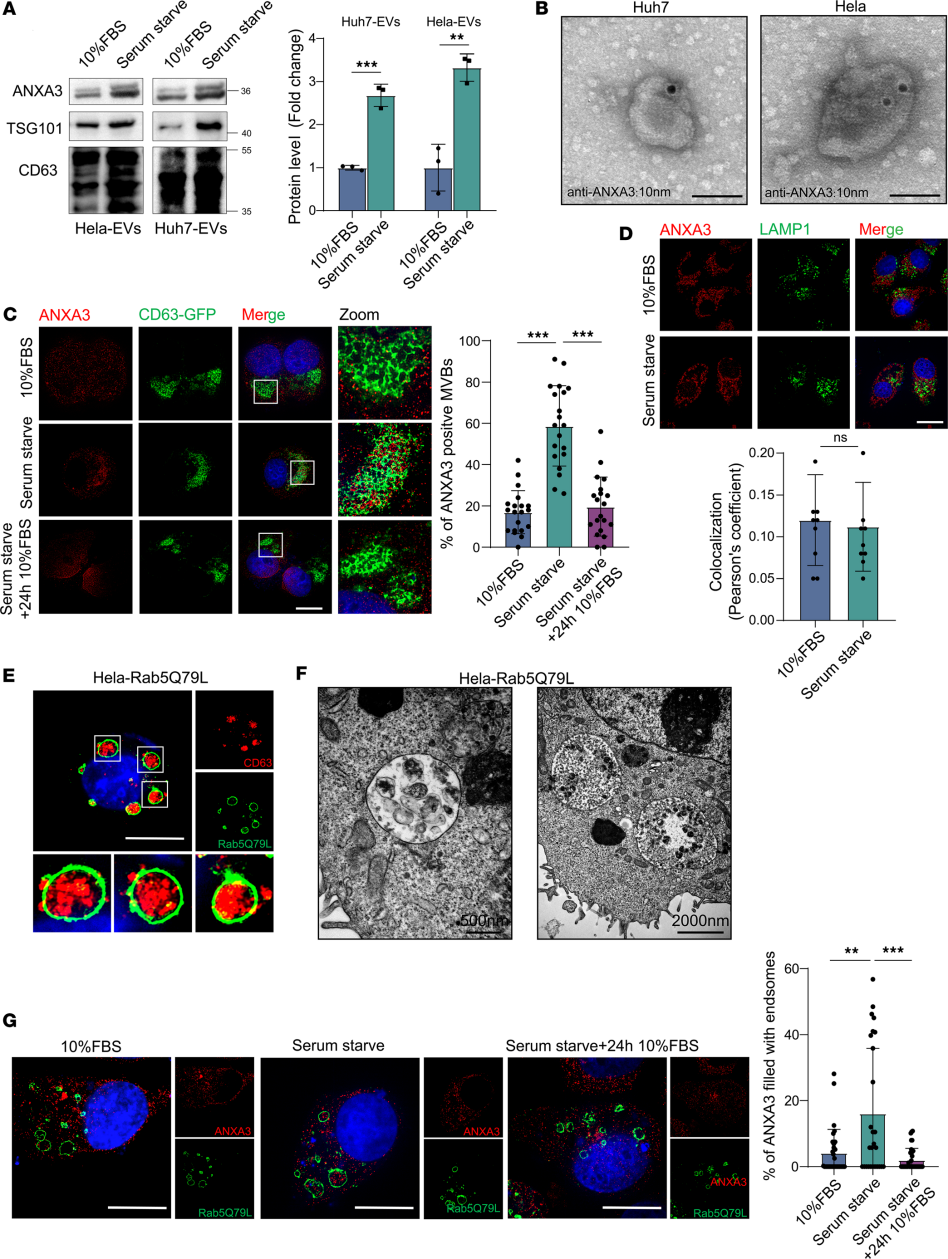
ANXA3 is enriched in starvation-induced EVs. (**A**) Western blot analysis of ANXA3 in Huh7-EVs and HeLa-EVs, with TSG101 and CD63 as EV markers. Right, quantification relative to the 10% FBS group. *n* = 3. (**B**) IEM showing ANXA3 localization in EVs using gold-labeled anti-ANXA3 antibody. Gold particles were mainly detected on EVs, indicating specific ANXA3 enrichment. Scale bar: 100 nm. (**C**) Polar-SIM images of ANXA3 (red) and CD63-GFP (green) in cells cultured in 10% FBS, under serum starvation, or recultured in 10% FBS after starvation. Nuclei were stained with DAPI (blue). Scale bar: 10 μm. Right, percentage of ANXA3^+^ MVB among CD63^+^ MVB. *n* = 20 cells. (**D**) Confocal images of ANXA3 (red) and LAMP1 (green); nuclei were stained with DAPI (blue). Bottom, Pearson’s correlation analysis of colocalization. *n* = 10 cells. (**E**) Dual-fluorescence imaging of Rab5Q79L (green) and CD63 (red) in HeLa-Rab5Q79L cells showing CD63 accumulation within Rab5Q79L^+^ endosomal lumens. Scale bar: 10 μm. (**F**) TEM of HeLa-Rab5Q79L cells showing abnormally enlarged MVB. Scale bars: 500 nm (left) and 2000 nm (right). (**G**) Polar-SIM images of ANXA3 (red) and Rab5Q79L (green) in HeLa-Rab5Q79L cells under the indicated conditions; nuclei were stained with DAPI (blue). Scale bar: 10 μm. Right, quantification of the percentage of ANXA3^+^ area within Rab5Q79L^+^ endosomes under the indicated conditions. *n* = 31, 27, 35 Rab5Q79L^+^ endosomes. Data are presented as mean ± SD. Statistical analysis was performed using ordinary 1-way ANOVA followed by Tukey’s multiple-comparison test. Comparisons between 2 groups were done with 2-tailed Student’s *t* test. **P < 0.01, ***P < 0.001.

Studies have reported that ANXA3 can be sorted into exosomes (31, 35), where it participates in intercellular communication, thereby promoting tumor progression (31) and facilitating osteoclast differentiation (36). However, the mechanisms underlying ANXA3 sorting remain poorly understood. Although some studies have reported that ANXA3^+^ EVs fall within the 30–200 nm size range, it remains challenging to distinguish between MVB-dependent EVs and plasma membrane–derived MVs within this size range (31, 35). To clarify the specific EV subpopulation enriched for ANXA3, we carried out the following investigations. First, to examine the expression levels of ANXA3 in EVs after serum starvation, Western blot confirmed that ANXA3 was upregulated in EVs derived from serum-starved HeLa, Huh7, and HCT116 cells (Figure 4A and Supplemental Figure 4A). To further validate the presence of ANXA3 on EVs, immunogold TEM was performed. ANXA3^+^ gold particles were clearly observed on EVs derived from both Huh7 and HeLa cells, confirming the localization of ANXA3 on EVs (Figure 4B). To further determine the cellular distribution of ANXA3, HeLa cells stably expressing CD63-GFP were generated by transduction with a CD63-GFP lentiviral construct. Confocal microscopy revealed a high degree of colocalization between CD63-GFP and endogenous CD63, confirming the successful establishment of CD63-GFP HeLa cells (Supplemental Figure 4B). To further clarify the subcellular distribution of ANXA3, Polar-SIM imaging showed that serum starvation led to an increased proportion of ANXA3^+^ MVB, while resupplementation with serum reduced the proportion of ANXA3^+^ MVB (Figure 4C).

In contrast, there was no pronounced change in the colocalization of ANXA3 with LAMP1, a lysosomal marker, under these conditions (Figure 4D). Moreover, our results show that starvation stress induced the formation of amphisomes. Previous studies have also demonstrated that amphisomes and autophagy are involved in EV secretion (21). To determine whether autophagy or amphisome formation participates in ANXA3 secretion under serum starvation, we generated *ATG3*-knockdown, autophagy-deficient cells. Under starvation conditions, autophagy was pronouncedly impaired in these cells (Supplemental Figure 4C). Western blot indicated that ATG3 does not participate in the secretion of ANXA3^+^ EVs (Supplemental Figure 4D). This suggests that, under serum starvation, ANXA3 is enriched explicitly in distinct MVB compartments. However, since MVB are rare subcellular compartments, it is challenging to distinguish discrete intraluminal cargo at the resolution of conventional fluorescence microscopy. To address this, we constructed a constitutively active Rab5Q79L model, which induces the formation of enlarged MVB, thereby facilitating the distinction between limiting membranes and the intraluminal compartments of MVB (37, 38). First, we established the HeLa cells model expressing Rab5Q79L and found that CD63 was efficiently enriched in the endosomal compartments (Figure 4E). At the subcellular level, we observed the accumulation of numerous classical endosomal structures, with their lumens filled with ILVs and small acidic vesicles (Figure 4F). Next, we confirmed that starvation stress led to an increased localization of ANXA3 within Rab5Q79L^+^ endosomal compartments. In contrast, after 24 hours of nutrient restoration, ANXA3 localization within these Rab5Q79L compartments was pronouncedly reduced compared with the starvation group (Figure 4G). Collectively, these data demonstrate that starvation stress promotes the sorting of ANXA3 into MVB, thereby promoting ANXA3^+^ EV secretion.

### Sorting of ANXA3 into MVB is dependent on ALIX.

**Figure 5:**
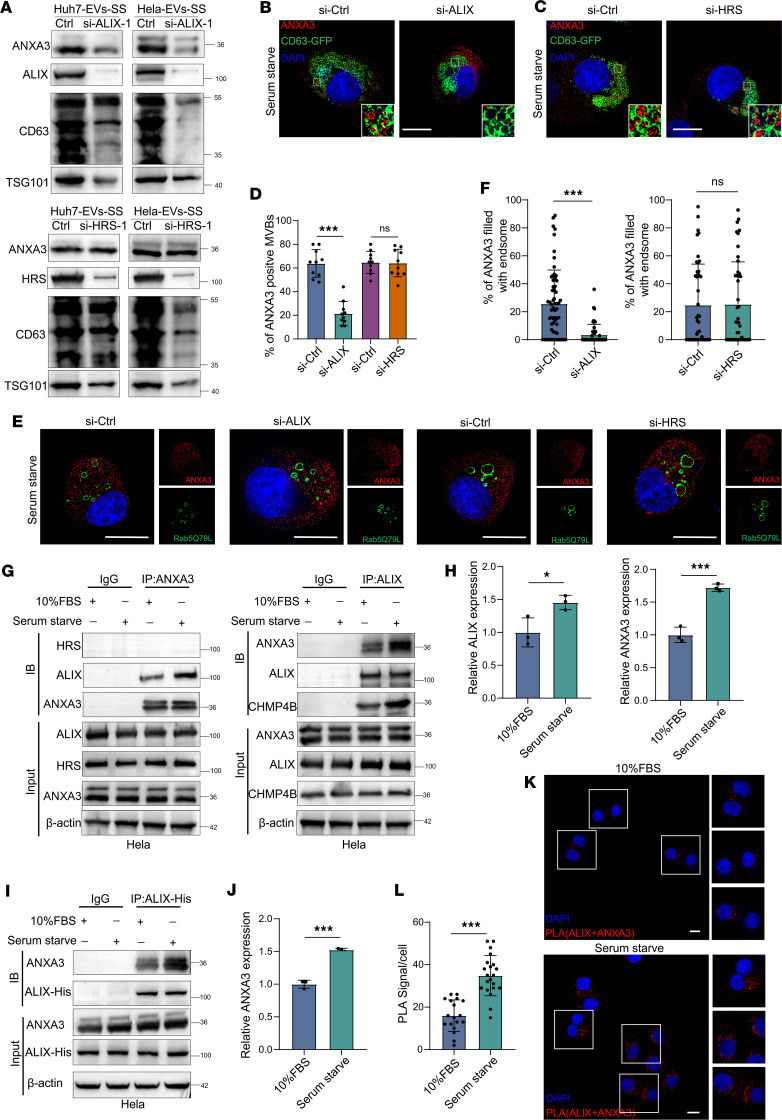
Sorting of ANXA3 into MVB is dependent on ALIX. (**A**) Western blot analysis of ANXA3 in Huh7-EVs and HeLa-EVs under serum starvation (SS) after siRNA-mediated ALIX or HRS knockdown. CD63 and TSG101 served as EV markers. (**B** and **C**) Polar-SIM images of ANXA3 (red) and CD63-GFP (green) in serum-starved cells after ALIX (**B**) or HRS (**C**) knockdown. Nuclei were stained with DAPI (blue). Insets show enlarged views. Scale bar: 10 μm. (**D**) Quantification of ANXA3^+^ MVB among CD63^+^ MVB in **B** and **C**. *n* = 10 cells. (**E**) Polar-SIM images of ANXA3 (red) and Rab5Q79L (green) in serum-starved HeLa-Rab5Q79L cells after siALIX or siHRS transfection. Nuclei were stained with DAPI (blue). Scale bar: 10 μm. (**F**) Quantification of ANXA3 occupancy in Rab5Q79L^+^ endosomes in **E**. *n* = 61, 48, 45, 45 Rab5Q79L^+^ endosomes. (**G**) Reciprocal Co-IP assays in HeLa cells cultured under 10% FBS or SS showing the interaction between ANXA3 and ALIX. Left, IP: ANXA3; right, IP: ALIX. IgG served as a negative control, and whole-cell lysates were used as input. (**H**) Quantification of Co-IP results in **G**. Left, ALIX coprecipitated with ANXA3; right, ANXA3 coprecipitated with ALIX, under 10% FBS or SS. *n* = 3. (**I** and **J**) HeLa cells were cotransfected with ALIX-His, and Co-IP (IP: ALIX-His) was performed to detect the ANXA3–ALIX-His interaction under 10% FBS or SS (**I**). (**J**) Quantification of ANXA3 coprecipitated with ALIX-His. *n* = 3. (**K** and **L**) PLA showing ALIX–ANXA3 interaction (red puncta) under 10% FBS (left) or SS (right), with nuclei stained by DAPI (blue). Boxed regions are enlarged. Scale bar: 10 μm (**K**). (**L**) Quantification of PLA signals. *n* = 18, 21 cells. Data are presented as mean ± SD. Statistical analysis was performed using ordinary 1-way ANOVA followed by Tukey’s multiple-comparison test. Comparisons between 2 groups were done with 2-tailed Student’s *t* test. *P < 0.05, ***P < 0.001.

Eukaryotic cells possess a highly conserved system for cargo sorting into MVB (12, 39, 40). The mechanisms for sorting different types of cargo are highly diverse; current studies suggest that even for individual EVs, parallel and distinct sorting pathways may exist. Moreover, the exact sorting mechanism can function in different subcellular compartments, which contributes to the heterogeneity of EV cargo to a certain extent (41). Specifically, ubiquitinated cargoes located at the plasma membrane or within the cytoplasm are sorted into ILVs through both ESCRT-dependent and ESCRT-independent mechanisms (39, 40). To elucidate the mechanism underlying ANXA3 sorting into EVs, we first disrupted major cargo sorting pathways in HeLa and Huh7 cells by silencing *ALIX* (an ESCRT-associated protein) and *HRS* (ESCRT-0) using siRNA (Supplemental Figure 5A). Western blot analysis of EVs under different conditions showed that *si-ALIX* pronouncedly reduced ANXA3, CD63, and TSG101 levels in EVs. In contrast, *si-HRS* did not substantially alter ANXA3 levels in EVs but decreased levels of CD63 and TSG101, indicating that HRS also plays a critical role in ILV formation (Figure 5A). Returning to the cellular context of serum starvation–induced stress, we used GFP-CD63–expressing HeLa cells to assess the effect of *si-ALIX*. Returning to the intracellular response under serum starvation stress, in HeLa cells expressing GFP-CD63, confocal fluorescence imaging showed that *si-ALIX* treatment pronouncedly reduced the number of ANXA3^+^ MVB following starvation (Figure 5, B and D). In contrast, *si-HRS* had no marked effect on the number of ANXA3^+^ MVB (Figure 5, C and D). In HeLa cells expressing Rab5Q79L, we interfered with *ALIX* and *HRS* by siRNA and assessed the distribution of ANXA3 after starvation stress. The results showed that *si-ALIX* impaired the sorting of ANXA3 into the MVB lumen, whereas *si-HRS* had no marked effect (Figure 5, E and F). These findings suggest that ALIX is the dominant mediator of ANXA3 sorting into ILVs within MVB, whereas HRS is not an important contributor.

Next, to further clarify the functional interaction between ANXA3 and the sorting protein ALIX, we performed Co-IP experiments to analyze the binding of ANXA3 with HRS and ALIX in HeLa and Huh7 cells under starvation stress. The results show that, compared with the control group, starvation stress pronouncedly induced the association between ALIX and ANXA3 (Figure 5, G and H, and Supplemental Figure 5, B and C). At the same time, there was no apparent interaction between ANXA3 and HRS under the same conditions (Supplemental Figure 5, D and E). By constructing exogenous tagged protein expression vectors for HRS-Myc and ALIX-His, we confirmed that serum starvation induction led to a marked association between ANXA3 and ALIX-His. In contrast, there was no marked interaction between ANXA3 and HRS-Myc (Figure 5, I and J, and Supplemental Figure 5F). These results indicate that serum starvation promotes the binding of ALIX to ANXA3 rather than HRS. Finally, proximity ligation assay (PLA), which detects transient protein-protein interactions at the nanometer scale, revealed a positive signal between ANXA3 and ALIX in the serum starvation group (Figure 5, K and L). In summary, these findings support that ALIX, but not HRS, is necessary for the sorting of ANXA3 into ILVs within MVB under starvation stress.

### ANXA3^+^ EVs promote tumor cell migration and proliferation.

**Figure 6:**
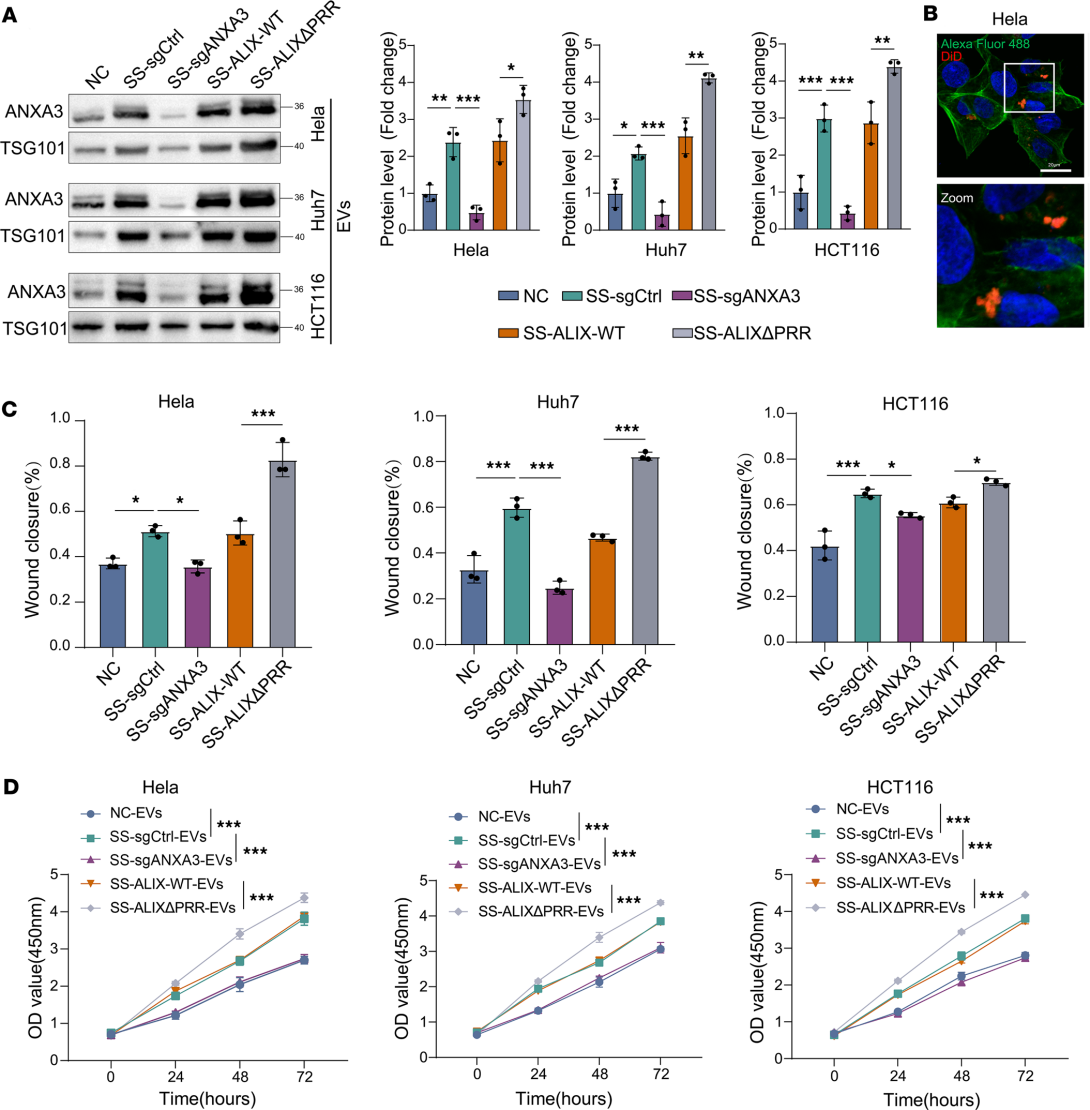
ANXA3^+^ EVs promote tumor cell migration and proliferation. (**A**) Western blot analysis of ANXA3 in EVs from HeLa, Huh7, and HCT116 cells under the indicated conditions: NC, SS-sgCtrl, SS-sgANXA3, SS-ALIX-WT, and SS-ALIXΔPRR. TSG101 served as an EV marker. Right, quantification of ANXA3 levels. *n* = 3. (**B**) Confocal images of EV uptake by HeLa cells. Cell membranes were labeled with Alexa Fluor 488 (green) and EVs with DiD (red). Insets show enlarged intracellular EVs. Scale bar: 20 µm. (**C**) Quantification of wound closure at 24 hours in the scratch assay. *n* = 3. (**D**) CCK-8 analysis of cell proliferation at 0, 24, 48, and 72 hours (OD450). *n* = 3. Data are presented as mean ± SD. Statistical analyses were performed using either ordinary 1-way ANOVA followed by Tukey’s multiple-comparison test or 2-way ANOVA followed by Šidák’s multiple-comparison test. *P < 0.05, **P < 0.01, ***P < 0.001.

There is a high degree of heterogeneity among EVs in the tumor microenvironment, and these vesicles play essential roles in tumor adaptation, survival, and progression (9, 15, 42). The above experimental results confirm that serum starvation reshapes the characteristics of EVs. To further clarify the regulatory effects of these EVs on recipient cells, we used the CRISPR-Cas9 system to knock out the *ANXA3* gene, thus generating ANXA3-deficient EVs (Supplemental Figure 6A). ALIX’s proline-rich region (PRR) acts as an autoinhibitory domain through interaction with its BRO1 domain, maintaining ALIX in a closed, self-inhibited conformation (40). We constructed an ALIXΔPRR mutant (deletion of the inhibitory domain facilitates membrane association) to activate the ALIX-dependent sorting pathway (40) to determine whether this activation can promote the sorting of ANXA3 into ILVs. We successfully established an ALIXΔPRR-mCherry cell line (Supplemental Figure 6, B and C). Subsequently, we collected the following types of EVs: NC-EVs, SS-sgCtrl-EVs, SS-sgANXA3-EVs, SS-ALIX-WT-EVs, and SS-ALIXΔPRR-EVs. Western blot analysis showed that the SS-ALIXΔPRR group exhibited the highest expression of ANXA3 (Figure 6A), further highlighting the critical role of ALIX in regulating ANXA3 sorting.

Subsequently, after prelabeling EVs with DiD and coculturing them with HeLa cells for 24 hours, confocal microscopy demonstrated that the EVs could be taken up by tumor cells (Figure 6B). Wound healing and CCK8 assays were used to evaluate the proliferation and migration abilities of HeLa, Huh7, and HCT116 cells after coculture with the above EVs. The results show that serum starvation–induced EVs promoted tumor cell proliferation and migration, with the SS-ALIXΔPRR-EV group exhibiting a pronouncedly enhanced effect. In contrast, the SS-sgANXA3-EV group displayed reduced proliferative capacity compared with the SS-sgCtrl-EV group (Figure 6, C and D, and Supplemental Figure 6D). These findings highlight ANXA3 as a key cargo responsible for tumor cell migration and the proliferation effect of ALIX-dependent EVs in vitro.

### ANXA3^+^ EVs drive s.c. tumor growth in mice.

**Figure 7:**
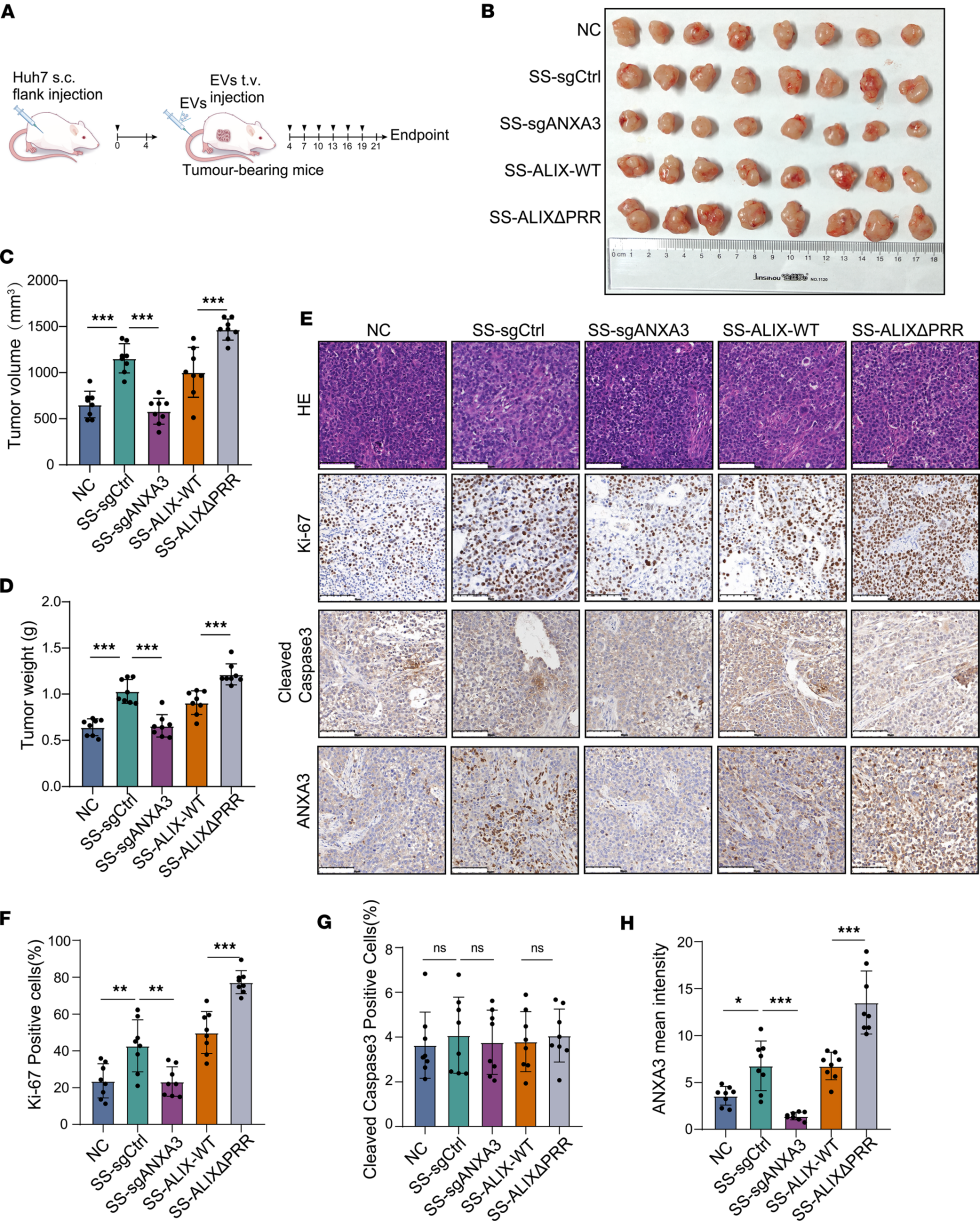
ANXA3^+^ EVs promote s.c. tumor growth in mice. (**A**) Schematic of the animal experiment. Huh7 cells were s.c. injected into nude mice. Three days later, EVs from the indicated groups were administered by tail vein injection every 3 days, and tumors were collected on day 21. (**B**) Representative tumor images from mice treated with EVs from NC, SS-sgCtrl, SS-sgANXA3, SS-ALIX-WT, and SS-ALIXΔPRR groups. (**C** and **D**) Quantification of tumor volume (**C**) and tumor weight (**D**). *n* = 8. (**E**) Representative IHC images of tumor tissues, including H&E, Ki-67, cleaved caspase-3, and ANXA3 staining. Scale bar: 100 μm. (**F**) Quantification of Ki-67^+^ cells in tumor tissues. *n* = 8. (**G**) Quantification of cleaved caspase-3^+^ cells in tumor tissues. *n* = 8. (**H**) Quantification of ANXA3 staining intensity in tumor tissues. *n* = 8. Data are presented as mean ± SD. Statistical analysis was performed using ordinary 1-way ANOVA followed by Tukey’s multiple-comparison test. *P < 0.05, **P < 0.01, ***P < 0.001.

After establishing s.c. tumor models in BALB/c-nude mice, the tumor-bearing mice received tail vein injections of the various EV preparations described above in separate groups. Twenty-one days later, s.c. tumors were harvested (Figure 7, A and B). Tumor volume (Figure 7C) and mass (Figure 7D) were subsequently analyzed. The results confirmed that enrichment of ANXA3 in EVs promotes tumor growth. Meanwhile, IHC analysis showed that EVs derived from serum starvation stress could induce the expression of ANXA3 and Ki67 in tumors. Specifically, the SS-ALIXΔPRR-EV group exhibited pronouncedly increased ANXA3 and Ki67 expression, while the SS-sgANXA3-EV group showed reduced ANXA3 and Ki67 expression compared with the SS-sgCtrl-EV group. However, the expression level of cleaved Caspase-3 remained unchanged among the groups (Figure 7, E–H). These results demonstrate that enrichment of ANXA3^+^ EVs induced by starvation stress promotes the progression of s.c. tumors in mice.

To further validate whether the tumor-promoting effects of SS-EVs depend on the ANXA3/ALIX axis, s.c. tumor models were established using cancer cells with genetic KO of *ALIX* or *ANXA3*, which was confirmed by Western blotting (Supplemental Figure 6A and Supplemental Figure 7A). Tumor-bearing mice were treated with NC-EVs or SS-EVs. As expected, SS-EVs markedly promoted tumor growth in control cells, whereas *ALIX* KO pronouncedly suppressed tumor growth, as reflected by reduced tumor size, weight, and volume, even in the presence of SS-EVs (Supplemental Figure 7, B–D). IHC analyses revealed reduced Ki-67 expression in tumors derived from the *ALIX*-KO group (Supplemental Figure 7, E and F). Similarly, ANXA3 KO pronouncedly inhibited tumor growth and largely weakened the tumor-promoting effects of SS-EVs (Supplemental Figure 7, G–I), with consistently reduced Ki-67 expression (Supplemental Figure 7, J and K). Collectively, these findings demonstrate that the protumorigenic effects of serum starvation–induced EVs are critically dependent on ANXA3 and ALIX, confirming the functional importance of the ANXA3–ALIX–EV axis in tumor progression.

### ANXA3^+^ EVs promote CRC organoid formation.

**Figure 8:**
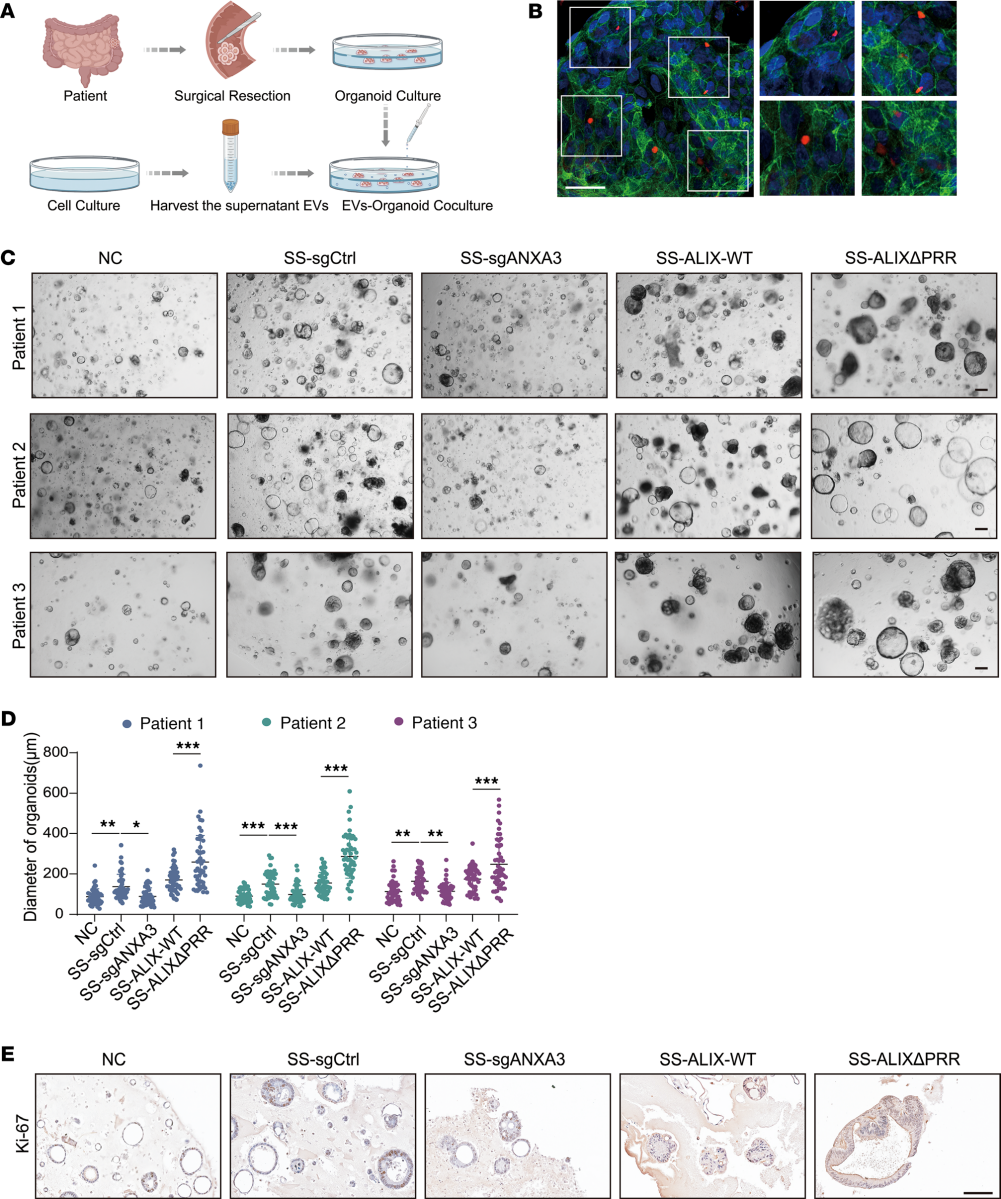
ANXA3^+^ EVs promote the growth of CRC organoids. (**A**) Schematic of organoid preparation and EV coculture. CRC tissues were digested to establish organoids, which were then cocultured with EVs. (**B**) Confocal images showing EV uptake by organoids. Cell membranes were labeled with Alexa Fluor 488 (green) and EVs with DiD (red). Insets show intracellular EVs. Scale bar: 50 μm. (**C**) Representative bright-field images of organoids from 3 patients cocultured with EVs from NC, SS-sgCtrl, SS-sgANXA3, SS-ALIX-WT, and SS-ALIXΔPRR groups. Scale bar: 100 μm. (**D**) Quantification of organoid diameter in the 3 patient-derived organoids under the indicated conditions. *n* = 150 organoids. (**E**) IHC analysis of Ki67 in Patient 1-derived CRC organoids after coculture with HCT116-derived EVs from the 5 indicated groups. Scale bar: 200 μm. Data are presented as mean ± SD. Statistical analysis was performed using ordinary 1-way ANOVA followed by Tukey’s multiple-comparison test. *P < 0.05, **P < 0.01, ***P < 0.001.

Organoids are 3-dimensional, self-renewing cultures that can recapitulate the complexity of organ tissues in vitro (43). We performed multidimensional characterization to assess the authenticity and stability of colorectal cancer (CRC) organoids, including representative bright-field images of continuous growth on days 1, 3, and 5 (Supplemental Figure 8A). Serial paraffin sections were used for H&E staining and immunohistochemical (IHC) analysis to evaluate the histopathological features of organoids in comparison with their corresponding tumor tissues (Supplemental Figure 8B). IHC was performed for Ki67 and characteristic CRC markers, including CK20, CDX-2, Villin, and CK7. The results showed that the organoids cultured in vitro exhibited a high degree of similarity to patient-derived tumor tissues (Supplemental Figure 8C). Meanwhile, confocal imaging showed the expression of Ki67, CK20, and CDX-2 in CRC organoids, confirming their colorectal origin (Supplemental Figure 8D). TME further revealed the ultrastructural features of cell clusters within the organoids, demonstrating characteristic properties of CRC cells, including abundant microvilli structures (Supplemental Figure 8E). To further evaluate the effects of starvation-induced EVs on CRC organoid formation and growth, we cocultured CRC organoids with different EV preparations (Figure 8A). After labeling HCT116 cell–derived EVs with DiD dye and coculturing them with organoids for 24 hours, confocal imaging revealed that the CRC organoids had taken up the EVs (Figure 8B). Subsequently, we isolated 5 groups of EVs derived from HCT116 cells: NC, SS-sgCtrl, SS-sgANXA3, SS-ALIX-WT, and SS-ALIXΔPRR. These different EVs were cocultured with CRC organoids, and morphological changes were recorded after 72 hours. The results showed that enrichment of ANXA3 in EVs pronouncedly promoted the growth of CRC organoids (Figure 8, C and D). Next, organoids derived from Patient1 were cocultured with 5 groups of HCT116 cell–derived EVs (NC, SS-sgCtrl, SS-sgANXA3, SS-ALIX-WT, SS-ALIXΔPRR), and IHC staining was performed to detect changes in the proliferation marker Ki67. The results showed that EVs from serum starvation stress could induce Ki67 expression in organoids, with the SS-ALIXΔPRR-EV group showing the most marked increase. In contrast, the SS-sgANXA3-EV group exhibited reduced Ki67 expression compared with the SS-sgCtrl-EV group (Figure 8E). Taken together, our study demonstrates that ANXA3^+^ EVs secreted under serum starvation conditions serve as key “survival signals” released in tumor cells in response to stress.

## Discussion

Malignant tumors exhibit dynamic regulation between cell death and proliferation, enabling them to sustain continuous growth (1, 4, 44, 45). During tumor progression, cancer cells often encounter varying degrees of nutrient deprivation (1, 44). In response to adverse microenvironmental conditions, tumor cells and related stromal cells activate protective programs to ensure their survival (1, 44, 46). Here we focus on the fact that serum starvation stress promotes the release of EVs in tumor cells, which regulate tumor stress homeostasis. Specifically, we demonstrated that serum starvation triggers MVB-dependent secretion of ANXA3^+^ EVs, and this process involves an unconventional ALIX-dependent ESCRT-III recruitment pathway, rather than the HRS pathway, for sorting ANXA3 into ILVs within MVB. Our experiments revealed that EVs derived from serum starvation cells were able to rescue s.c. tumor growth in *ALIX* or *ANXA3* knockout cells. Moreover, ANXA3^+^ EVs enhanced the malignant biological behaviors of recipient cells. Our findings suggest that tumor cells can secrete EVs to mediate the cellular communications involved in cancer progression.

Microenvironmental stresses — including oncogenic mutations (9), hypoxia (47), neutrophil expansion (14), and nutrient deprivation (22) — can induce specific EV secretion (11). Serum starvation, as a classic model, has frequently been used to induce autophagy and to study intracellular stress response mechanisms (20, 24). However, as a critical component of the eukaryotic endomembrane system, the biogenesis and fate of MVB under serum starvation stress remain unclear. Although some articles have revealed that serum starvation can exhibit specific secretory properties in neuroblastoma cells (16), microglia (48), and myeloma cells (5), these studies not only failed to disclose the internal causes of serum starvation EV changes but also, to a certain extent, overlooked plasma membrane–derived EVs (10, 16, 29). Electron microscopy revealed that the biogenesis of MVB in tumor cells increased pronouncedly after 24 hours of serum starvation and returned to a low basal level upon restoration of nutrient supply for 24 hours. When cancer cells suffered serum starvation for 48 hours, large secretory autophagic vesicles were observed, likely resulting from the fusion of intracellular autophagic vesicles under prolonged stress and implying that tumor cells are on the brink of necrosis and apoptosis under sustained stress conditions (21). We have been studying the mechanisms underlying MVB biogenesis and MVB transport/docking machinery (10, 14, 49). So, what is the fate of MVB induced by serum starvation? Previous studies at least suggest that a subset of MVB is destined for the secretion of EVs. Our experiments reveal that tumor cells exhibited pronouncedly increased EV secretion under serum starvation conditions, with an apoptosis rate of less than 5%. TME demonstrated events of MVB at the cell membrane boundary fusing with the plasma membrane and releasing their contents — an event also documented in previous research (50, 51).

Meanwhile, our experiments confirmed that starvation stress promotes the secretion of EVs by tumor cells without marked changes in EV size. Together, these results demonstrate that serum starvation at least regulates a subset of secretory MVB and enhances EV secretion (5, 16, 48). Bec et al. reported that serum starvation induces the sorting of proteasome 19S regulatory particles (19SRP) and translation initiation complexes into EVs during either the biogenesis of EVs or endosomal microautophagy in mouse embryonic fibroblasts and melanoma cells (24). Under serum starvation, cells respond to maintain their integrity and homeostasis, particularly through the remodeling of vesicle membranes and their trafficking to adapt to the new environment (24). Label-free mass spectrometry (MS) revealed the proteomic profile of EVs, showing marked activation of vesicle membrane protein families. Among the core vesicle membrane proteins, 4 key genes were identified: ANXA1, ANXA3, ANXA5, and ANXA6. Studies have shown that annexins play multiple roles in membrane trafficking and MVB (36, 52, 53). Notably, Randy Schekman (University of California, Berkeley, Berkeley, California, USA) demonstrated that Ca^2+^-dependent ANXA6 localizes to MVB, which participate in exosome secretion and membrane repair (28), and also reported that calpain regulates the secretion of MVs containing ANXA1 and ANXA2, facilitating the rapid repair of damaged cell membranes (29).

Our experiments found that ANXA3 was upregulated in EVs under starvation stress (32, 33). Moreover, experiments have confirmed that this regulatory phenomenon has tumor specificity. ANXA3 can participate in tumor malignant progression through autocrine and/or paracrine mechanisms (32, 36). Recently, EV extraction kits with TiO_2_ microsphere-based tissue EV capture have also confirmed the presence of ANXA3 in EVs (35). Additionally, mechanical forces have been shown to promote periodontal ligament stem cells to regulate Rab27B-mediated secretion of ANXA3^+^ EVs, thereby facilitating osteoclast differentiation. Moreover, confocal experiments confirmed that ANXA3 can be sorted into Rab5Q79L endosomes in tumor cells. Combined with ANXA3 immunoEM results, this demonstrates that ANXA3 is enriched in starvation-induced EVs. Early endosomes undergo a series of protein sorting events, leading to cargo entry into ILVs, and gradually mature into MVB rich in ILVs. These MVB are then either targeted to lysosomes for degradation or fused with the plasma membrane to secrete EVs (10). The relatively conserved sorting mechanisms in eukaryotic cells mainly include ESCRT-dependent and ESCRT-independent pathways (10, 13). The ESCRT machinery consists of complexes (ESCRT-0, -I, -II, and -III) and ESCRT-III–associated proteins, such as VPS4 (10, 13). The central core component of ESCRT-III is CHMP4 (SNF7 in yeast), which forms helical structures capable of storing mechanical energy and plays a central role in membrane remodeling and ILV formation (10, 13). The nucleation and recruitment of ESCRT-III complexes on endosomes primarily involve the canonical ESCRT-0, -I, and -II pathways, as well as the ESCRT-0/BRO1–dependent mechanism. By interfering with HRS and ALIX, using superresolution confocal microscopy, we revealed that ANXA3 is sorted into ILVs via the ALIX-mediated pathway (40). ALIX consists of an N-terminal BRO1 domain, a central V-shaped domain, and a flexible C-terminal PRR (40). ALIX initiates ESCRT-III complex assembly by binding to CHMP4B through its BRO1 domain, thereby driving the formation of ESCRT-III and facilitating membrane scission to generate ILVs (40). This pathway also induces the sorting of tetraspanins, such as CD63, CD81, and CD9, into ILVs (40). Our results at least indicate that starvation stress triggers an ALIX-dependent ESCRT-III endosomal recruitment pathway, facilitating the sorting of ANXA3 into ILVs. In ALIXΔPRR mutant experiments, ANXA3 was enriched in EVs. The fundamental basis of EV heterogeneity lies in the complex intracellular cargo sorting and secretion mechanisms. Even within a single mature MVB (containing numerous ILVs), completing cargo sorting typically requires the cooperation of multiple sorting mechanisms (18, 54). We demonstrate that starvation stress activated the ALIX-dependent ESCRT-III endosomal recruitment pathway, which generated ANXA3^+^ EVs. Interestingly, previous studies have shown that the annexins can induce MVB formation and participate in cargo sorting (52). For example, annexin A1 mediates ER-to-endosome contact, thereby facilitating cargo sorting into ILVs (53). Therefore, we have reason to speculate that this process may involve the direct binding of ANXA3 to ALIX, forming a molecular interaction network with other partners to promote cargo sorting, which will further experimental investigation in the future (40). Additionally, free ANXA3 can be internalized via clathrin-mediated endocytosis and activate the JNK pathway, thereby regulating HCC stemness and tumor growth (32). ANXA3 can bind Ca^2+^ ions and phospholipid membranes, thereby controlling the uptake of target cells, which suggests, to some extent, that starvation-induced EVs may have an advantage in mediating intercellular communication (36). Starvation-derived ANXA3^+^ EVs promoted the proliferation and migration of tumor cells. Furthermore, CRC organoid and mouse models reveal that starvation-derived ANXA3^+^ EVs facilitate tumor progression.

In sum, our study reveals a mechanism that allows tumor cells to maintain homeostasis. Under starvation stress, tumor cells tend to remodel their MVB formation and EV secretion, producing more aggressive EVs that facilitate intercellular communication. Serum starvation triggers an ALIX-dependent ESCRT-III endosomal recruitment pathway, leading to ANXA3 cargo sorting into ILV, thereby eliciting tumor-promoting functions. While this process may appear to be a waste of energy or resources from the perspective of the stressed cell, it could be seen as a sophisticated coevolutionary strategy that has developed during tumor evolution.

## Methods

### Sex as a biological variable.

All animal experiments were performed using female mice only, primarily because their relatively docile behavior facilitates routine handling and experimental manipulation. Therefore, the current in vivo findings do not address potential sex-specific differences. In the patient-derived organoid studies, specimens were collected from both male and female patients; however, analyses were not stratified by sex. We observed that starvation-derived EVs produced broadly similar effects across organoids with different genetic backgrounds.

### Cell culture.

THLE-2, HcerEpic, and Huh7 cells were cultured in DMEM; NCM460 cells were cultured in RPMI-1640; HeLa cells were cultured in MEM; and HCT116 cells were cultured in McCoy’s 5A medium, all supplemented with 10% FBS and 1% penicillin-streptomycin, at 37°C in a humidified incubator with 5% CO_2_.

### Animal studies.

Female BALB/cA-nu nude mice (6–8 weeks) were purchased from Huafukang Laboratory Animal Co. Ltd. and maintained under SPF conditions at the Experimental Animal Center of China Medical University. Huh7 cells (1 × 10^6^ in 100 μL PBS) were injected s.c. into the right axilla. After tumor establishment, mice received EVs from different conditions via tail vein injection every 3 days. Body weight and tumor size were monitored throughout. Tumors were collected on day 21 for further analysis.

### Patient’s tumor tissue and organoid culture.

CRC tissues were obtained in 2024 from 3 patients at the Fourth Affiliated Hospital of China Medical University, Patient 1, 75-year-old male, right-sided colon adenocarcinoma; Patient 2, 74-year-old male, right-sided colon adenocarcinoma; and Patient 3, 70-year-old female, right-sided colon adenocarcinoma. Resected tissues were minced and digested at 37°C for 30–40 minutes. The supernatant was filtered through a 100 μm strainer, centrifuged at 300*g* for 5 minutes, and the pellet was washed twice with PBS (300*g*, 5 minutes each). Cells were embedded in Matrige and seeded in 48-well plates. Organ cells were cultured in CRC Organoid Culture Medium, all supplemented at 37°C in a humidified incubator with 5% CO_2_.

### siRNA, shRNA, CRISPR/Cas9, lentiviruses, and transfection.

siRNA transfection was performed with Lipofectamine 3000 (Invitrogen, Thermo Fisher Scientific) per the manufacturer’s protocol. Cells in 6-well plates were transfected at 70%–80% confluence. Per well, 5 μg siRNA and 10 μL P3000 in 125 μL Opti-MEM were mixed with 5 μL Lipofectamine 3000 in 125 μL Opti-MEM, incubated at room temperature for 10–15 minutes, added to cells, and cultured for 24–72 hours before protein analysis. For *ANXA3* sgRNA, *ALIX* sgRNA, *ATG3* shRNA, GFP-Rab5Q79L, EGFP-CD63, ALIXΔPRR-mCherry, Myc-HRS, and His-ALIX expression, cells were infected for 48 hours. Viral volume was calculated as: viral volume (μL) = MOI × cell number/viral titer (TU/mL) × 1000. Cells were then selected with puromycin (1–5 μg/mL, optimized by cell sensitivity) until nontransfected controls were eliminated. Surviving cells were considered stably expressing cells. Knockdown or overexpression efficiency was confirmed by western blotting and other assays.

### Purification of EVs.

EVs were purified by improved differential centrifugation as described previously (13, 55). Conditioned medium was sequentially centrifuged at 4°C at 300*g* for 10 minutes, 2,000*g* for 20 minutes, and 10,000*g* for 30 minutes, filtered through a 0.22 μm filter, and ultracentrifuged at 100,000*g* for 90 minutes (Optima XPN-100, Beckman). Pelleted EVs were washed in PBS, ultracentrifuged again at 100,000*g* for 90 minutes at 4°C, and finally resuspended in 100 μL PBS.

### Proteomic analysis.

LC-MS/MS. Purified EVs in PBS were processed using 5–7 μg protein per sample by DTT reduction, IAA alkylation, and overnight tryptic digestion at 37°C (1:50, w/w). Peptides were desalted with C18 StageTips, eluted in 50% acetonitrile/0.1% TFA, vacuum-dried, reconstituted in 0.1% formic acid, and 200 ng was injected for LC-MS/MS. Separation was performed on a Bruker nanoElute coupled to a timsTOF Pro with CaptiveSpray. Data were acquired in PASEF mode and analyzed with PaSER 2023 using TIMScore and CCS for PSM and FDR control. Proteins were identified against the UniProt human database.

### Bioinformatics.

Differential expression was analyzed by 2-tailed Student’s *t* test in Python 3.9.5 (SciPy 1.11.4; NumPy 1.26.3). EV-associated proteins for comparison were obtained from Vesiclepedia (56). GO analysis of DEPs was performed with DAVID, and GO enrichment pathway networks were generated with WebGestalt. GSEA was performed using GSEA v4.1.0. Venn diagrams were generated with the VennDiagram package (57) (v1.7.3) in R 4.2.2. Volcano and GO bubble plots were generated using CNSknowall.

### NTA.

Nanoparticle size and concentration were measured using a ZetaView PMX 110 (Particle Metrix). Ultracentrifugation-purified EVs were resuspended in sterile PBS and, if needed, filtered through a 0.22 μm filter. Samples were mixed, loaded into the chamber, and analyzed at 23°C with automatic acquisition at multiple positions. Each sample was measured at least 3 times, and averages were calculated. Size distribution and concentration were determined using ZetaView software based on the Stokes–Einstein equation. Raw data were exported for statistical analysis and plotting.

### Electron microscopy.

Cells were washed with PBS, fixed overnight at 4°C in 0.1 M sodium cacodylate buffer with 2% glutaraldehyde and 2% paraformaldehyde, post-fixed in 1% OsO_4_ at 4°C for 1 hour, dehydrated in graded ethanol (50%, 70%, 90% for 10 minutes each; 100% twice for 5 minutes), embedded in epoxy resin, sectioned (70–100 nm) with a Leica Ultracut UCT and diamond knife, mounted on copper grids, stained with 2% aqueous uranyl acetate, and imaged on an H-7650 TEM at 80 kV. MVB number was normalized to image area (MVB/μm^2^), MVB diameter was measured along the short axis, and ILVs/MVB were counted.

### EV TEM.

EVs were washed twice with sterile PBS, suspended in 0.1 M sodium cacodylate buffer, fixed overnight at 4°C in 2% glutaraldehyde, postfixed in 1% OsO_4_ at 4°C for 1 hour, dehydrated as above, embedded in epoxy resin, sectioned (70–100 nm), mounted on copper grids, stained with 2% aqueous uranyl acetate, and imaged on a Hitachi H-7650 TEM at 80 kV. EV diameter was measured along the short axis in randomly selected fields and expressed in μm.

### Organoid TEM.

Organoids were washed 3 times with sterile PBS, fixed overnight at 4°C in 0.1M sodium cacodylate buffer with 2% glutaraldehyde and 2% paraformaldehyde, postfixed in 1% OsO_4_ at 4°C for 1 hour, dehydrated in graded ethanol, embedded in epoxy resin, sectioned (70–100 nm) with a Leica Ultracut UCT and diamond knife, mounted on copper grids, stained with 2% aqueous uranyl acetate, and imaged on a Hitachi H-7650 TEM at 80 kV.

### EV IEM.

EVs were blocked with PBS containing 50 mM glycine for 10 minutes and 5% BSA for 10 minutes, incubated with anti-ANXA3 or anti-CD63 primary antibodies in PBS/1% BSA at 4°C for 2 hours, washed, incubated with 10 nm gold-conjugated goat anti-rabbit or anti-mouse IgG secondary antibodies in PBS/1% BSA at room temperature for 1 hour, washed, negatively stained with 2% uranyl acetate for 30 seconds, air-dried, and imaged on a Hitachi H-7650 TEM at 80 kV.

### EV cryo-EM.

Liquid EV samples were applied to copper grids, blotted to a thin film, plunge-frozen in liquid ethane, transferred to liquid nitrogen for storage, screened and analyzed at medium resolution on a 200 kV cryo-EM, and imaged for high-resolution data collection on a 300 kV cryo-EM using FFI and AFIS, at ~500 images/h.

### NanoView assay.

Samples were centrifuged at 300*g* for 10 minutes and 2,000*g* for 20 minutes, and the supernatant was filtered through a 0.22 μm membrane. Chips were blocked with 100 μL blocking buffer for 1 hour at room temperature. Pretreated samples were diluted 1:1–1:10 in blocking buffer, and 50 μL was added per well and incubated overnight (16 hours) at room temperature. Chips were washed 3 times for 5 minutes each with wash buffer and were then incubated with 50 μL diluted fluorescent detection antibody for 2 hours at room temperature in the dark. After 3 additional 5-minute washes, chips were rinsed with ultrapure water, air-dried on filter paper protected from light, scanned using the ExoView R100 (NanoView Biosciences), and analyzed with NanoViewer software.

### Western blot.

Cells or EVs were lysed in RIPA buffer with protease inhibitor on ice, sonicated, and centrifuged at 12,000*g*; the supernatant was collected. Protein concentration was measured using a protein quantification kit. Samples were mixed with loading buffer and denatured by boiling (soluble proteins) or incubation at 37°C (membrane proteins), separated by PAGE, and transferred to PVDF membranes. Membranes were blocked, incubated with primary antibody overnight at 4°C, washed with TBST, incubated with secondary antibody, and washed again. Bands were detected by ECL and quantified using Fiji. The specific dilution concentration can be found in Supplemental Table 4.

### Co-IP.

Cells were washed 3 times with prechilled PBS, collected, lysed in prechilled lysis buffer with protease inhibitors, and incubated at 4°C for 30 minutes. Lysates were centrifuged at 12,000*g* for 10–15 minutes at 4°C, and the supernatant was incubated with primary antibody at 4°C for 2–4 hours. Protein A/G magnetic beads (50 μL; Biolinkedin) were washed 3 times with 500 μL prechilled PBS, before being incubated with the antigen-antibody mixture at 4°C for 2–4 hours. Beads were collected magnetically, washed 3 times with 100 μL lysis buffer, mixed with 100 μL 1× SDS-PAGE loading buffer, and heated at 100°C for 10 minutes for subsequent analysis.

### Polar-SIM and IF.

Cells or organoids were fixed with 4% PFA for 15 minutes (cells) or 25 minutes (organoids), washed with PBS, permeabilized with 0.1% Triton X-100 for 20 minutes, blocked with 5% BSA for 40 minutes, incubated with primary antibodies overnight at 4°C, washed, incubated with fluorescent secondary antibodies for 2 hours at room temperature, washed, stained with DAPI for 3 minutes, and rewashed with PBS. Samples were protected from light throughout. Imaging was performed using a Polar-SIM superresolution microscope (Airy Technology). SIM images were reconstructed with Airy SIM software using “preprocessing (Dark)” and “post-processing (MRA/MLE)” under consistent parameters across samples.

Cargo colocalization with CD63-GFP. Cargo^+^ MVB were manually counted per field and divided by total CD63-GFP^+^ MVB in the same field to obtain the percentage of cargo localized to CD63-GFP^+^ MVB. Analysis was performed under minimal background and consistent parameters. Statistics were calculated per cell and shown as mean ± SD. Cargo colocalization with GFP-Rab5Q79L. All successfully transfected cells were analyzed. GFP-Rab5Q79L^+^ rings were outlined in ImageJ using oval or freehand selection, and ring area was measured with “Analyze Particles.” Cargo^+^ areas within each ring were segmented in the corresponding channel. Cargo occupancy was calculated as cargo^+^ area divided by total ring area. Analysis was performed under minimal background and consistent parameters. Statistics were calculated per ring and shown as mean ± SD.

### PLA.

In situ PLA was performed using the DUO92101 kit per the manufacturer’s instructions. Cells on coverslips were fixed with 4% paraformaldehyde, permeabilized with 0.05% Triton X-100, blocked with 40 μL Duolink blocking solution at 37°C for 60 minutes, and incubated with primary antibodies in Duolink antibody diluent overnight at 4°C. The next day, PLUS and MINUS PLA probes (1:5 in antibody diluent) were applied for 1 hour at 37°C, followed by ligation with 1× Duolink ligation solution for 30 minutes at 37°C and amplification with 1× amplification solution for 100 minutes at 37°C. After DAPI counterstaining, images were acquired by confocal microscopy.

### Proliferation assays.

Proliferation of HeLa, Huh7, and HCT116 cells was measured using the CCK-8 kit (Abbkine). Cells were seeded in 96-well plates under different conditions, incubated with 10 μL CCK-8 at 37°C for 30 minutes, and absorbance was measured at 450 nm.

### Migration assays.

Wound-healing assays were performed in 6-well plates by scratching cell monolayers with a 200 μL pipette tip. Wells were gently washed 2–3 times with PBS to remove detached cells, low-serum medium was added, and cells were incubated. Images were taken at 0 and 24 hours to assess migration.

### Flow cytometric analyses.

Apoptosis was analyzed using an Abbkine apoptosis detection kit. Cells were digested with EDTA-free trypsin, resuspended in 500 μL 1× Annexin V binding buffer, stained with 5 μL Annexin V–AbFluor 488 and 2 μL PI for 15 minutes at room temperature in the dark, and analyzed by flow cytometry within 30 minutes.

### IHC.

IHC was used for initial histopathological evaluation. Organoids were fixed in methanol for 30 minutes; tumor tissues were fixed in 4% PFA overnight, dehydrated, and paraffin-embedded. Sections (4 μm) were deparaffinized, rehydrated, and subjected to antigen retrieval in sodium citrate buffer (pH 7.4) at subboiling temperature in a microwave for 15 minutes. After cooling and washing, endogenous peroxidase was blocked with 3% H_2_O_2_ for 10 minutes, followed by blocking with 5% BSA in PBS for 30 minutes at room temperature. Primary antibodies for organoids and matched tumors included Ki67, CK20, CDX-2, CK7, and Violin; those for s.c. tumors included Ki67, caspase-3, and ANXA3. Sections were then incubated with matched secondary antibodies, developed with DAB, counterstained, dehydrated, and mounted.

### Statistics.

All experiments were independently repeated at least 3 times. Data were analyzed using ImageJ (NIH), GraphPad Prism 9.0, and FlowJo 10.0.7. Quantitative data are presented as the mean ± SD. Comparisons between 2 groups were performed using an unpaired 2-tailed Student’s *t* test. Comparisons among multiple groups were performed using 1-way ANOVA, followed by appropriate post hoc multiple-comparison tests. Experiments involving 2 independent variables were analyzed using 2-way ANOVA, followed by appropriate post hoc multiple-comparison tests. *P* < 0.05 was considered statistically significant.

### Study approval.

All animal studies were approved by the Animal Ethics and Welfare Committee of China Medical University (approval no. CMU2023871) and performed in compliance with the committee’s institutional guidelines and regulations. 

The procurement of CRC tissue specimens was approved by the Ethics Committee of the Fourth Affiliated Hospital of China Medical University (approval no. EC-2023-KS-043). Written informed consent was obtained from all patients prior to sample collection. All procedures involving human specimens were conducted in accordance with the institutional guidelines and regulations of the Ethics Committee of the Fourth Affiliated Hospital of China Medical University.

### Data availability.

The raw proteomics data generated in this study have been deposited in the ProteomeXchange Consortium (dataset identifier: PXD073980) via the iProX partner repository (https://www.iprox.cn) under project ID IPX0015459000 (https://www.iprox.cn/page/project.html?id=IPX0015459000).

## Author contributions

XP contributed writing the original draft, conceptualization, investigation, review and editing, funding acquisition, project administration, and visualization. JL contributed conceptualization, investigation, writing, validation, formal analysis, methodology, resources, supervision, and project administration. GZ contributed writing, investigation, validation, formal analysis, methodology, data curation, software, and visualization. YX contributed formal analysis, software, validation, and formal analysis. ZH contributed validation, formal analysis, and methodology. YL contributed investigation, methodology, and resources. SW contributed investigation, methodology, and resources. ST contributed investigation, methodology, and resources. GH contributed validation, formal analysis, and methodology. HJ contributed investigation, resources, and validation. YG contributed resources. YY contributed validation. LY contributed writing the original draft, conceptualization, review and editing, methodology, resources, validation, supervision, and funding acquisition. HL contributed writing the original draft, conceptualization, investigation, review and editing, methodology, resources, funding acquisition, data curation, validation, supervision, formal analysis, software, project administration, and visualization.

## Conflict of interest

The authors have declared that no conflict of interest exists.

## Funding support

- National Natural Science Foundation of China (82303373 and 82372919)
- Liaoning Provincial Local Science and Technology Development Fund (2024020198-JH6/1008)
- Shenyang Young and Middle-aged Science and Technology Talents Support Program (RC230583)
- Science and Technology Projects of Shenyang (22-321-31-02 and 21-104-0-04).

## Supplementary Material

Supplemental data

Unedited blot and gel images

Supporting data values

## Version 1 06/08/2026

Electronic publication
