# Supplementary material for: Serum starvation drives ALIX-dependent extracellular vesicle biogenesis and determines tumor progression
Source: JCI Insight. 2026 Jun 8;11(11):e197924. doi: 10.1172/jci.insight.197924 (PMC13313550; doi:10.1172/jci.insight.197924)
Supplement: Supplemental data [file jciinsight-11-197924-s271.pdf]

Supplementary materials

Figure S1. Selection of the serum starvation time window.

(A–C) Apoptosis of Huh7 (A), Hela (B), and HCT116 (C) cells cultured in 10% FBS or serum-starved for 24 or 48 h was analyzed by Annexin V/PI staining and flow cytometry. Representative plots are shown at left, with quantification of Annexin V-positive cells at right. n=3.

(D) Cell viability (OD450) of Huh7, Hela, and HCT116 cells cultured in 10% FBS or under serum starvation was measured at 0, 24, 48, and 72 h. n=3.

Data are presented as mean ± SD. Statistical analyses were performed using either ordinary one-way ANOVA followed by Tukey's multiple comparisons test or two-way ANOVA followed by Sidak's multiple comparisons test. ns, not significant, $**P < 0.01$, $***P < 0.001$.

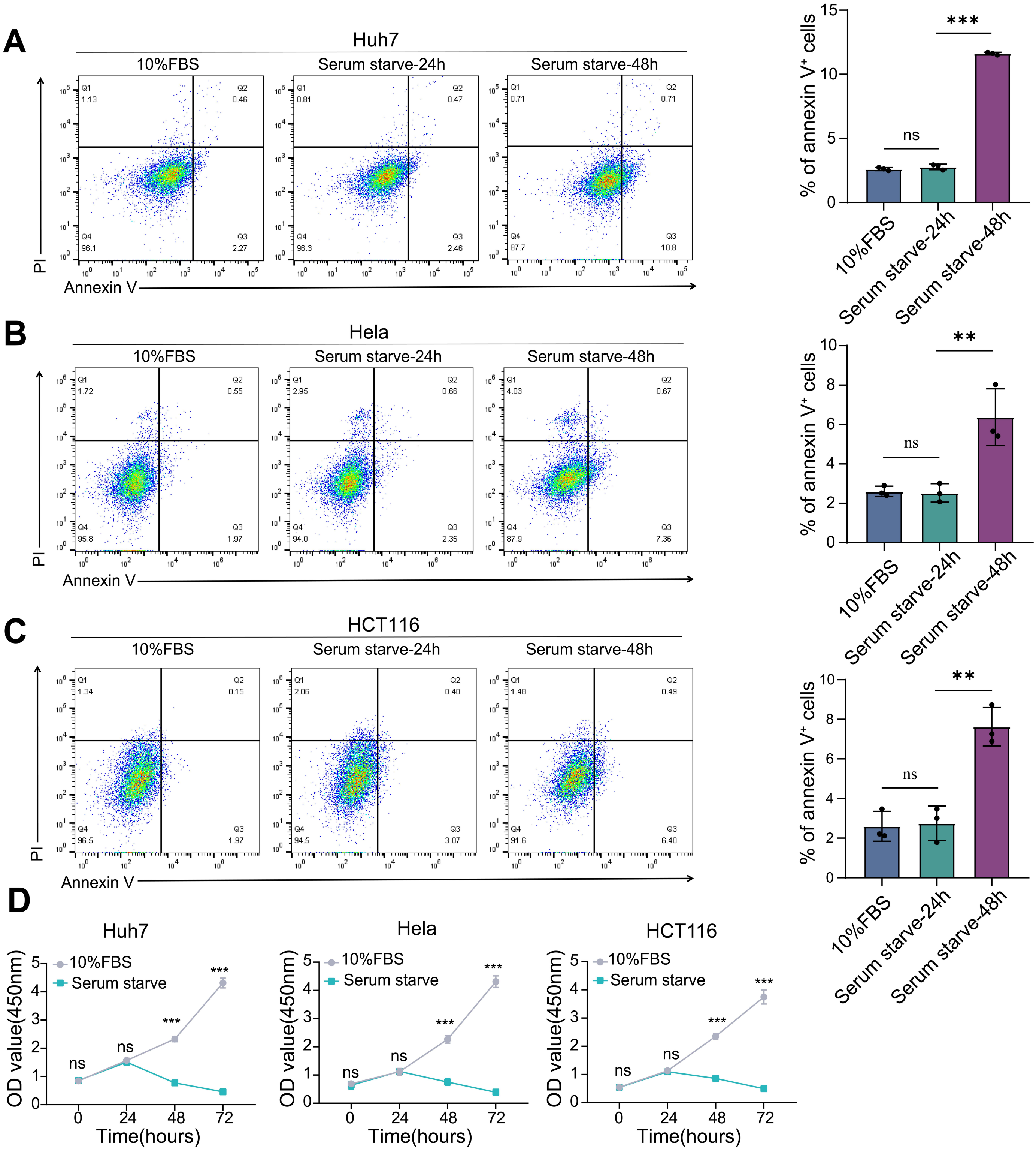
A
Huh7
10%FBS
Serum starve-24h
Serum starve-48h
Q1 1.13
Q2 0.46
Q4 96.1
Q3 2.27
Q1 0.81
Q2 0.47
Q4 96.3
Q3 2.46
Q1 0.71
Q2 0.71
Q4 87.7
Q3 10.8
PI
Annexin V
% of annexin V+ cells
ns
***
B
Hela
Q1 1.72
Q2 0.55
Q4 95.8
Q3 1.97
Q1 2.95
Q2 0.66
Q4 94.0
Q3 2.35
Q1 4.03
Q2 0.67
Q4 87.9
Q3 7.36
**
C
HCT116
Q1 1.34
Q2 0.15
Q4 96.5
Q3 1.97
Q1 2.06
Q2 0.40
Q4 94.5
Q3 3.07
Q1 1.48
Q2 0.49
Q4 91.6
Q3 6.40
D
Huh7
Hela
HCT116
10%FBS
Serum starve
OD value(450nm)
Time(hours)

Figure S2. Serum starvation induces EVs secretion

(A) Western blot analysis of classical EV markers (CD63, CD81, TSG101, CD9, ALIX, and HRS) in HCT116-EVs; calnexin served as a negative control for cellular contamination. Right, quantification of EV marker expression in HCT116-EVs. n=3.

(B) NTA of EVs size distribution and concentration in HCT116 cells under 10% FBS or serum starvation.

(C) NTA comparison of mean EVs size from Hela, Huh7, and HCT116 cells under 10% FBS or serum starvation. n=3.

(D) Total EVs concentration of Hela-, Huh7-, and HCT116-derived EVs measured by NTA. n=3.

(E) NTA measurement of EVs (<200 nm) in Hela-EVs, Huh7-EVs, and HCT116-EVs. n=3.

Data are presented as mean ± SD. Comparisons between 2 groups were done with 2-tailed Student's t test. ns, not significant, *$P < 0.05$, **$P < 0.01$, ***$P < 0.001$.

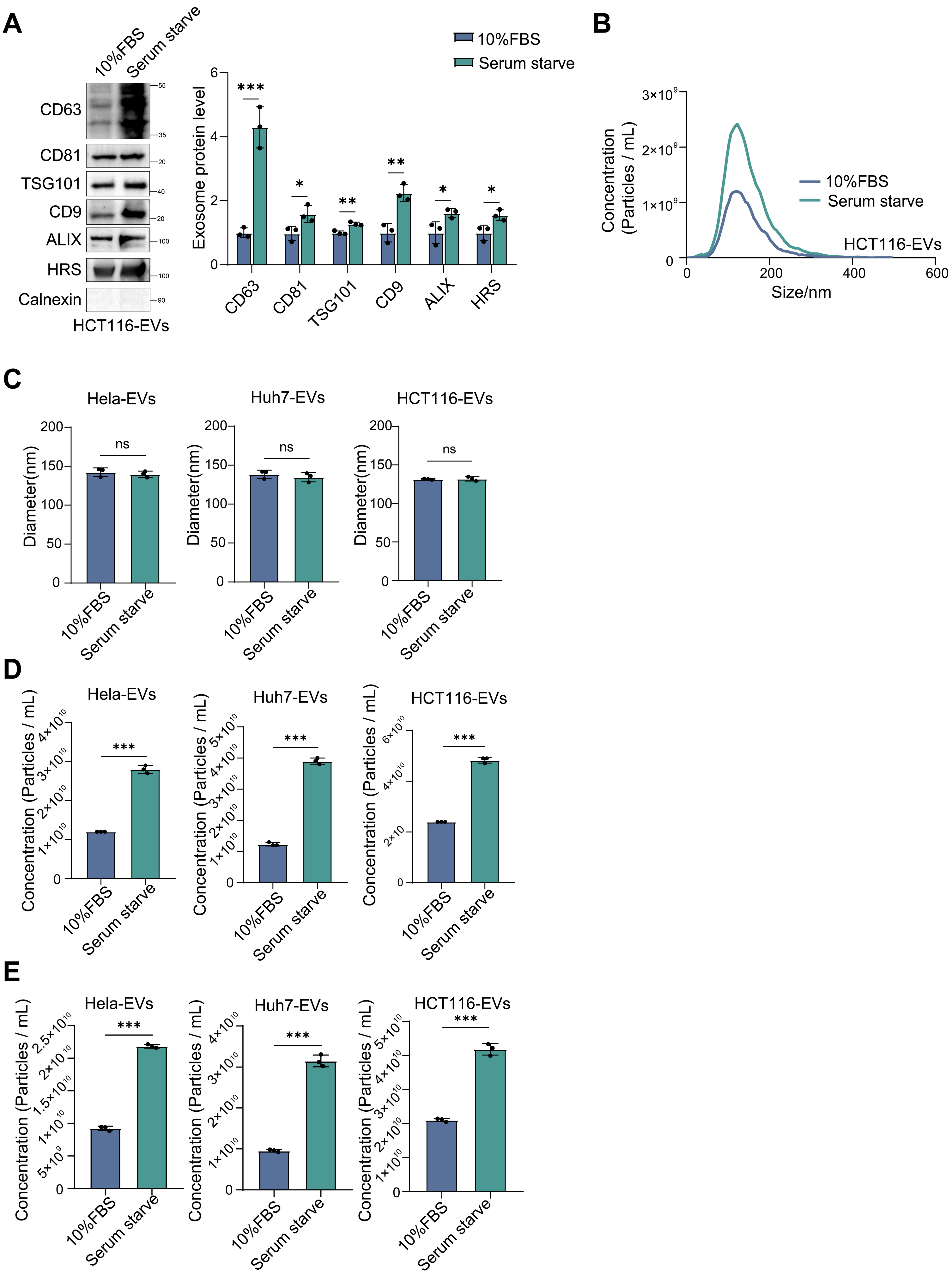
A
10%FBS
Serum starve
CD63
CD81
TSG101
CD9
ALIX
HRS
Calnexin
55
35
20
40
20
100
100
90
HCT116-EVs
10%FBS
Serum starve
Exosome protein level
0
2
4
6
***
*
**
**
*
*
CD63
CD81
TSG101
CD9
ALIX
HRS
B
Concentration (Particles / mL)
3×10⁹
2×10⁹
1×10⁹
0
10%FBS
Serum starve
HCT116-EVs
0
200
400
600
Size/nm
C
Hela-EVs
Huh7-EVs
HCT116-EVs
Diameter(nm)
200
150
100
50
0
ns
10%FBS
Serum starve
D
Hela-EVs
Huh7-EVs
HCT116-EVs
Concentration (Particles / mL)
4×10¹⁰
3×10¹⁰
2×10¹⁰
1×10¹⁰
5×10¹⁰
6×10¹⁰
0
***
10%FBS
Serum starve
E
Hela-EVs
Huh7-EVs
HCT116-EVs
Concentration (Particles / mL)
2.5×10¹⁰
2×10¹⁰
1.5×10¹⁰
1×10¹⁰
5×10⁹
4×10¹⁰
3×10¹⁰
5×10¹⁰
0
***
10%FBS
Serum starve

Figure S3. Identification of ANXA3 as a key EV cargo protein regulated by serum starvation

(A) GO cellular component hierarchical analysis (tree map) of the enrichment results in Fig. 3D. Red boxes indicate terms highlighted in Fig. 3D, blue boxes indicate vesicle membrane-related hierarchies, and yellow boxes indicate non-hierarchical associations.

(B) Quantification of ANXA3, ANXA1, ANXA5, and ANXA6 in Hela-EVs, Huh7-EVs, and HCT116-EVs. n=3.

(C) GSEA of exosomal membrane-associated proteins showing enrichment of vesicle membrane-related proteins under serum starvation.

(D) Western blot analysis of ANXA3 and TSG101 in EVs from THLE-2, Huh7, HcerEpic, Hela, NCM460, and HCT116 cells cultured in 10% FBS or under serum starvation.

(E) Quantification of ANXA3 protein levels. n=3.

Data are presented as mean ± SD. Comparisons between 2 groups were done with 2-tailed Student's t test. ns, not significant, *$P < 0.05$, **$P < 0.01$, ***$P < 0.001$.

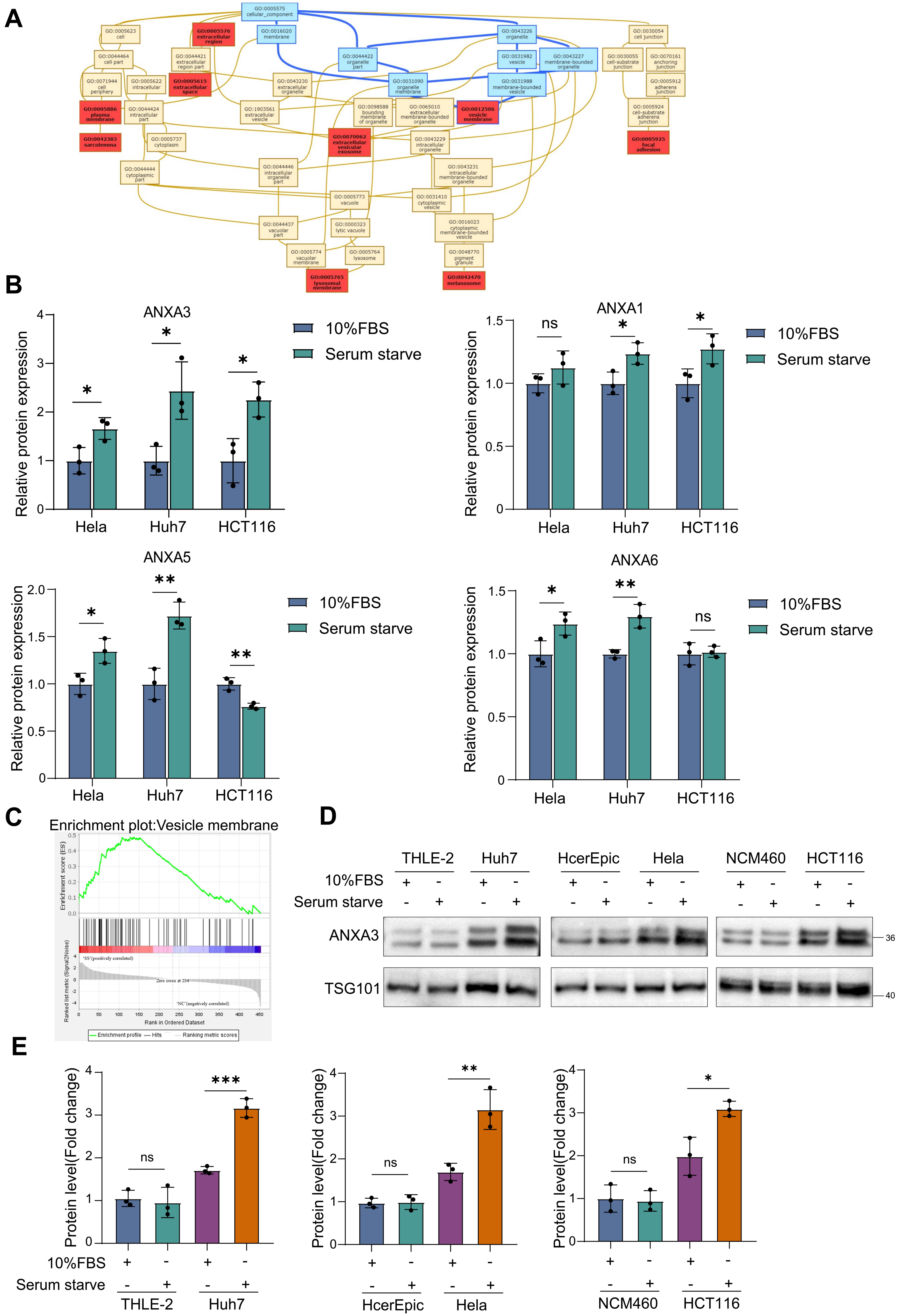

A
GO:0005575 cellular_component
GO:0005623 cell
GO:0005576 extracellular region
GO:0016020 membrane
GO:0043226 organelle
GO:0030054 cell junction
GO:0044464 cell part
GO:0044421 extracellular region part
GO:0044422 organelle part
GO:0031982 vesicle
GO:0043227 membrane-bounded organelle
GO:0030055 cell-substrate junction
GO:0070161 anchoring junction
GO:0071944 cell periphery
GO:0005622 intracellular
GO:0005615 extracellular space
GO:0043230 extracellular organelle
GO:0031090 organelle membrane
GO:0031988 membrane-bounded vesicle
GO:0005912 adherens junction
GO:0005886 plasma membrane
GO:0044424 intracellular part
GO:1903561 extracellular vesicle
GO:0098588 bounding membrane of organelle
GO:0065010 extracellular membrane-bounded organelle
GO:0012506 vesicle membrane
GO:0005924 cell-substrate adherens junction
GO:0042383 sarcolemma
GO:0005737 cytoplasm
GO:0070062 extracellular vesicular exosome
GO:0043229 intracellular organelle
GO:0005925 focal adhesion
GO:0044444 cytoplasmic part
GO:0044446 intracellular organelle part
GO:0043231 intracellular membrane-bounded organelle
GO:0005773 vacuole
GO:0031410 cytoplasmic vesicle
GO:0044437 vacuolar part
GO:0000323 lytic vacuole
GO:0016023 cytoplasmic membrane-bounded vesicle
GO:0005774 vacuolar membrane
GO:0005764 lysosome
GO:0048770 pigment granule
GO:0005765 lysosomal membrane
GO:0042470 melanosome
B
ANXA3
ANXA1
ANXA5
ANXA6
10%FBS
Serum starve
Relative protein expression
Hela
Huh7
HCT116
ns
*
**
C
Enrichment plot:Vesicle membrane
Enrichment score (ES)
'SS'(positively correlated)
Zero cross at 234
'NC'(negatively correlated)
Ranked list metric (Signal2Noise)
Rank in Ordered Dataset
Enrichment profile — Hits — Ranking metric scores
D
THLE-2
Huh7
HcerEpic
Hela
NCM460
HCT116
10%FBS
Serum starve
ANXA3
TSG101
36
40
E
Protein level(Fold change)
ns
***
**
*
10%FBS
Serum starve
THLE-2
Huh7
HcerEpic
Hela
NCM460
HCT116

Figure S4. Autophagy does not participate in ANXA3 secretion

(A) Western blot analysis of ANXA3 in EVs from HCT116 cells cultured in 10% FBS or under serum starvation, with TSG101 and CD63 as EV markers. Right, quantification of ANXA3. n = 3.

(B) Confocal images of CD63-GFP (green) and endogenous CD63 (red) in cells. Right, line-scan analysis of fluorescence intensity. Scale bar, 10 μm.

(C) Western blot analysis of LC3 I/II and ATG3 in Hela and Huh7 cells transfected with shCtrl or shATG3. β-actin served as a loading control.

(D) Western blot analysis of ANXA3 in Hela-EVs and Huh7-EVs after shCtrl or shATG3 transfection, with TSG101 as an EV marker. Bottom, quantification. n = 3.

Data are presented as mean ± SD. Comparisons between 2 groups were done with 2-tailed Student's t test. ns, not significant, $*P < 0.05$.

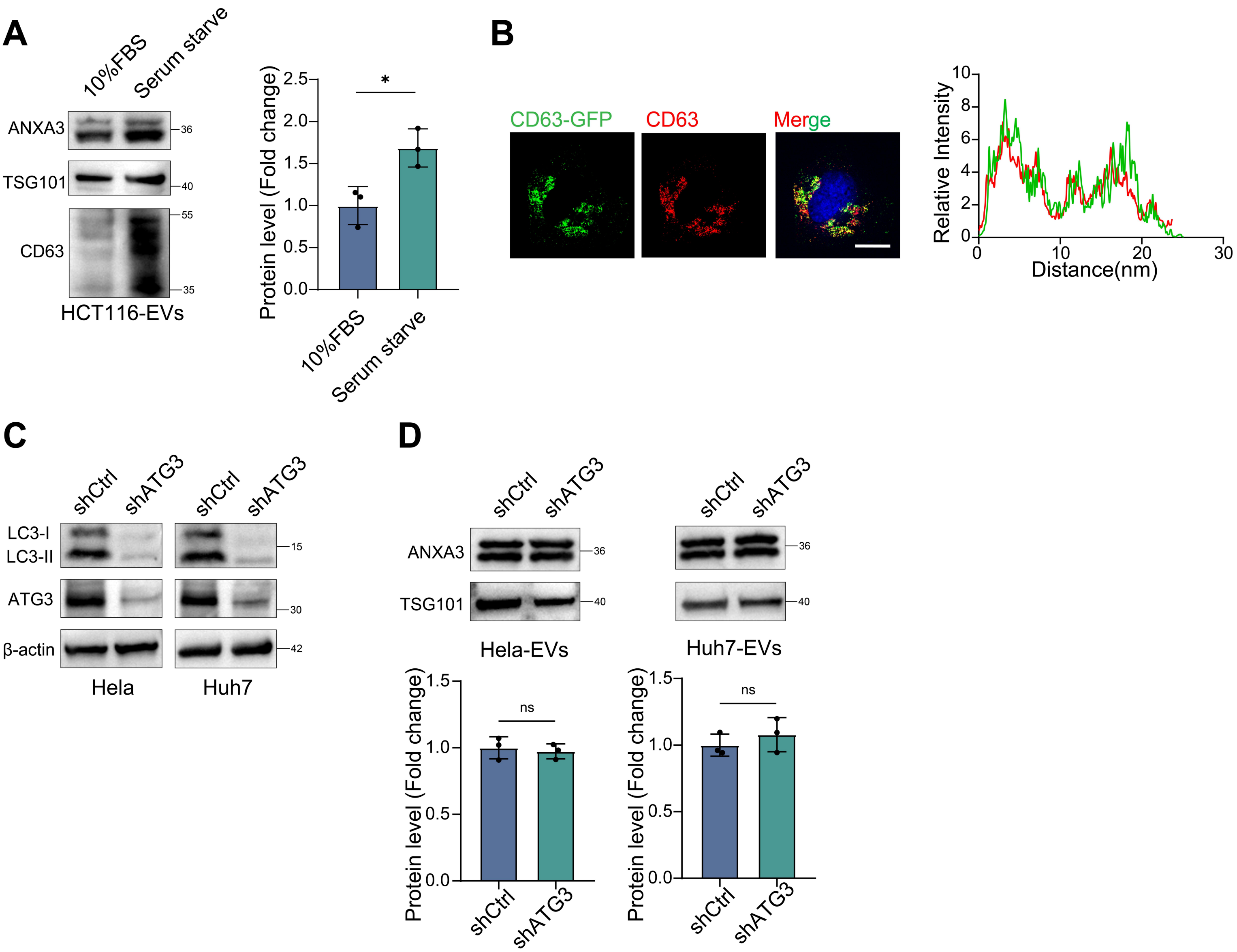
A
10%FBS
Serum starve
ANXA3
TSG101
CD63
36
40
55
35
HCT116-EVs
Protein level (Fold change)
2.5
2.0
1.5
1.0
0.5
0.0
*
10%FBS
Serum starve
B
CD63-GFP
CD63
Merge
Relative Intensity
10
8
6
4
2
0
0
10
20
30
Distance(nm)
C
shCtrl
shATG3
shCtrl
shATG3
LC3-I
LC3-II
ATG3
β-actin
15
30
42
Hela
Huh7
D
shCtrl
shATG3
shCtrl
shATG3
ANXA3
TSG101
36
40
36
40
Hela-EVs
Huh7-EVs
Protein level (Fold change)
1.5
1.0
0.5
0.0
ns
shCtrl
shATG3
Protein level (Fold change)
1.5
1.0
0.5
0.0
ns
shCtrl
shATG3

Figure S5. ANXA3 sorting into MVBs requires ALIX but not HRS

(A) Western blot validation of ALIX or HRS knockdown in Huh7 and Hela cells, with β-actin as a loading control.

(B) Co-IP analysis in Huh7 cells using anti-ANXA3, anti-ALIX, or anti-HRS to examine interactions among ANXA3, ALIX, HRS, and CHMP4B. IgG served as a negative control. Upper, immunoblots; lower, input controls.

(C) Quantification of ALIX co-precipitated with ANXA3 and ANXA3 co-precipitated with ALIX under 10% FBS or serum starvation. n = 3.

(D, E) Co-IP (IP: HRS) in Hela (D) and Huh7 (E) cells cultured in 10% FBS or under serum starvation, followed by immunoblotting for ANXA3 and HRS. Whole-cell lysates were used as input.

(F) Co-IP (IP: HRS-Myc) in Hela cells expressing HRS-Myc under 10% FBS or serum starvation to examine the interaction between ANXA3 and HRS-Myc. β-actin served as a loading control.

Data are presented as mean ± SD. Comparisons between 2 groups were done with 2-tailed Student's t test. ns, not significant, $^{*}P < 0.05$.

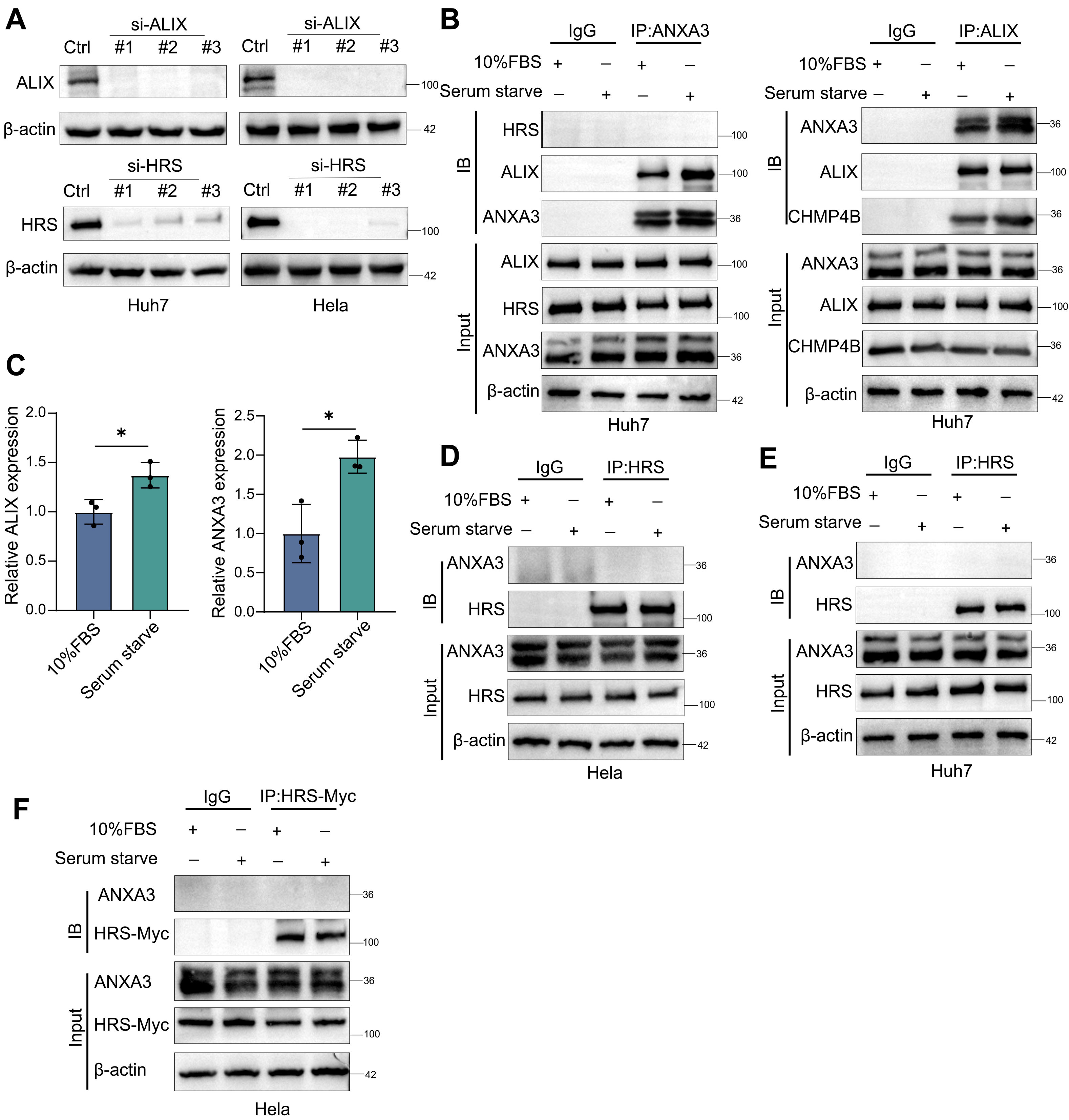
A
si-ALIX
Ctrl #1 #2 #3
ALIX
β-actin
si-HRS
HRS
Huh7
Hela
B
IgG
IP:ANXA3
IP:ALIX
10%FBS
Serum starve
IB
Input
CHMP4B
ANXA3
C
Relative ALIX expression
Relative ANXA3 expression
10%FBS
Serum starve
D
IP:HRS
E
F
IP:HRS-Myc
HRS-Myc

Figure S6. Construction and validation of cell lines expressing ALIX mutant lacking the PRR domain

(A) Western blot analysis of ANXA3 in Hela, Huh7, and HCT116 cells transfected with Ctrl or different sgANXA3 constructs (#1, #2, #3), with β-actin as a loading control.

(B) Confocal images of ALIX localization in Hela, Huh7, and HCT116 cells transfected with empty vector (Ctrl) or ALIXΔPRR-mCherry. Red, mCherry; blue, DAPI. ALIXΔPRR-mCherry was diffusely distributed in the cytoplasm. Scale bar, 10 μm.

(C) Western blot analysis of ALIX expression in Hela, Huh7, and HCT116 cells transfected with ALIXΔPRR-mCherry or empty vector, with β-actin as a loading control.

(D) Representative wound-healing images showing migration in each group at 0 and 24 h. Scale bar: 100 μm.

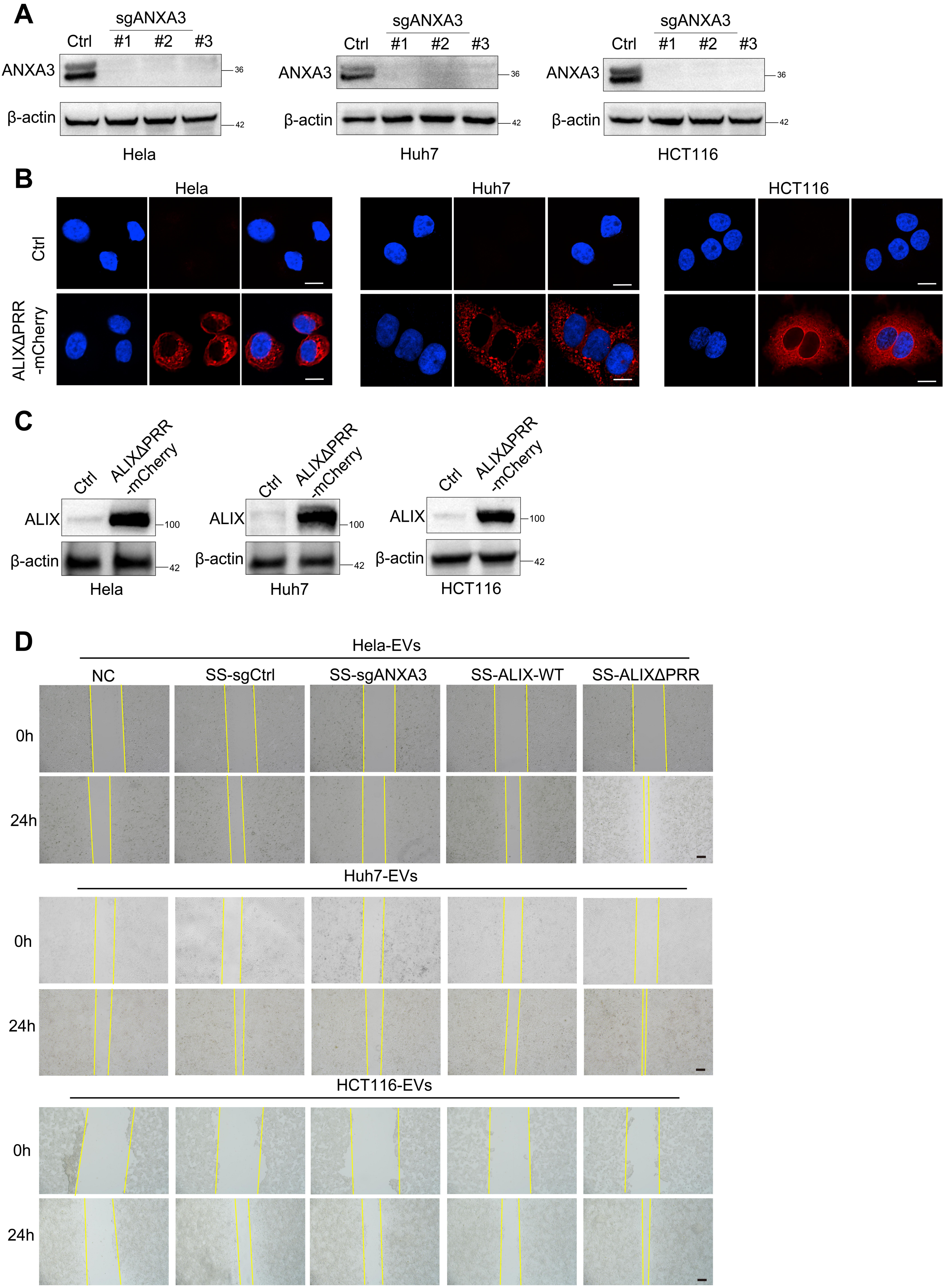
A
sgANXA3
Ctrl
#1
#2
#3
ANXA3
36
β-actin
42
Hela
Huh7
HCT116
B
Hela
Huh7
HCT116
Ctrl
ALIXΔPRR
-mCherry
C
Ctrl
ALIXΔPRR
-mCherry
ALIX
100
β-actin
42
Hela
Huh7
HCT116
D
Hela-EVs
NC
SS-sgCtrl
SS-sgANXA3
SS-ALIX-WT
SS-ALIXΔPRR
0h
24h
Huh7-EVs
HCT116-EVs

Figure S7. Genetic knockout of ALIX or ANXA3 attenuates the tumor-promoting effects of serum starvation-induced EVs in vivo

(A) Western blot validation of ALIX knockout in cancer cells transduced with sgALIX.

(B) Representative images of subcutaneous tumors derived from control or ALIX-knockout cells treated with NC-EVs or SS-EVs.

(C, D) Quantification of tumor weight (C) and tumor volume (D) in the indicated groups. n = 6.

(E) Representative H&E and Ki-67 staining of tumor sections from control or ALIX-knockout cells treated with NC-EVs or SS-EVs.

(F) Quantification of Ki-67-positive cells in tumors shown in (E). n = 6.

(G) Representative images of subcutaneous tumors derived from control or ANXA3-knockout cells treated with NC-EVs or SS-EVs.

(H, I) Quantification of tumor weight (H) and tumor volume (I) in the indicated groups. n = 6.

(J) Representative H&E and Ki-67 staining of tumor sections from control or ANXA3-knockout cells treated with NC-EVs or SS-EVs.

(K) Quantification of Ki-67-positive cells in tumors shown in (J). n = 6.

Data are presented as mean ± SD. Statistical analysis was performed using two-way ANOVA followed by Sidak's multiple comparisons test. *$P < 0.05$, **$P < 0.01$, ***$P < 0.001$.

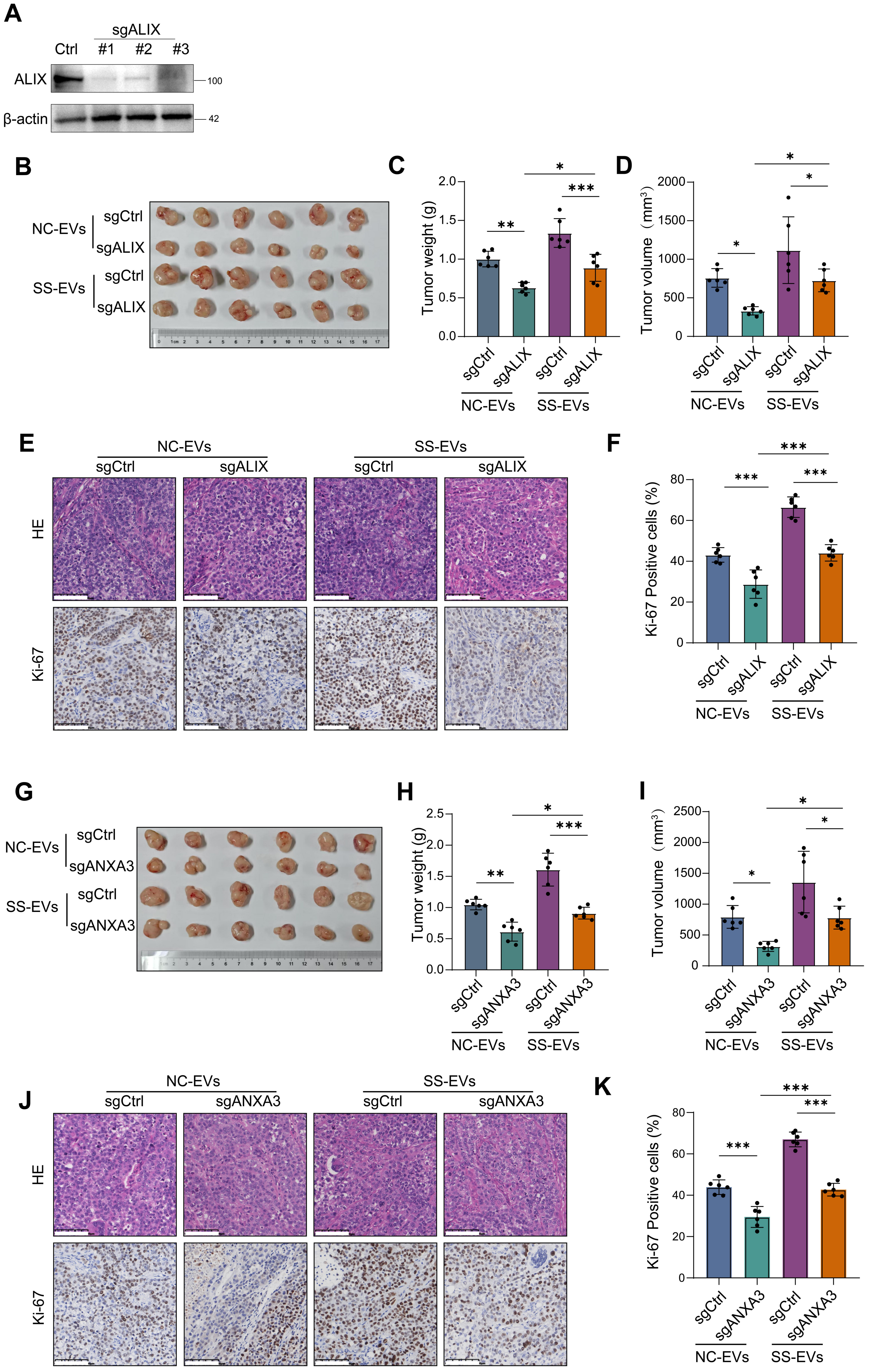

A
sgALIX
Ctrl
#1
#2
#3
ALIX
100
β-actin
42
B
NC-EVs
sgCtrl
sgALIX
SS-EVs
sgCtrl
sgALIX
C
Tumor weight (g)
2.0
1.5
1.0
0.5
0.0
**
*
***
sgCtrl
sgALIX
sgCtrl
sgALIX
NC-EVs
SS-EVs
D
Tumor volume (mm³)
2000
1500
1000
500
0
*
*
*
sgCtrl
sgALIX
sgCtrl
sgALIX
NC-EVs
SS-EVs
E
NC-EVs
SS-EVs
sgCtrl
sgALIX
sgCtrl
sgALIX
HE
Ki-67
F
Ki-67 Positive cells (%)
80
60
40
20
0
***
***
***
sgCtrl
sgALIX
sgCtrl
sgALIX
NC-EVs
SS-EVs
G
NC-EVs
sgCtrl
sgANXA3
SS-EVs
sgCtrl
sgANXA3
H
Tumor weight (g)
2.5
2.0
1.5
1.0
0.5
0.0
**
*
***
sgCtrl
sgANXA3
sgCtrl
sgANXA3
NC-EVs
SS-EVs
I
Tumor volume (mm³)
2500
2000
1500
1000
500
0
*
*
*
sgCtrl
sgANXA3
sgCtrl
sgANXA3
NC-EVs
SS-EVs
J
NC-EVs
SS-EVs
sgCtrl
sgANXA3
sgCtrl
sgANXA3
HE
Ki-67
K
Ki-67 Positive cells (%)
80
60
40
20
0
***
***
***
sgCtrl
sgANXA3
sgCtrl
sgANXA3
NC-EVs
SS-EVs

Figure S8. Characterization of CRC tissues and organoids

(A) Bright-field images of primary CRC-derived organoids from day 1 to day 5. Scale bar, 100 μm.

(B) H&E staining of tumor tissues and organoid sections. Scale bar, 200 μm.

(C) IHC staining of tumor tissues and organoid sections for Ki67, CK20, CDX2, Villin, and CK7. Scale bar, 200 μm.

(D) Confocal images showing Ki67, CDX2, and CK20 expression in organoids. Nuclei were stained with DAPI. Scale bar, 100 μm.

(E) TEM images showing organoid ultrastructure. Scale bar, 5 μm; enlarged view at right.

.

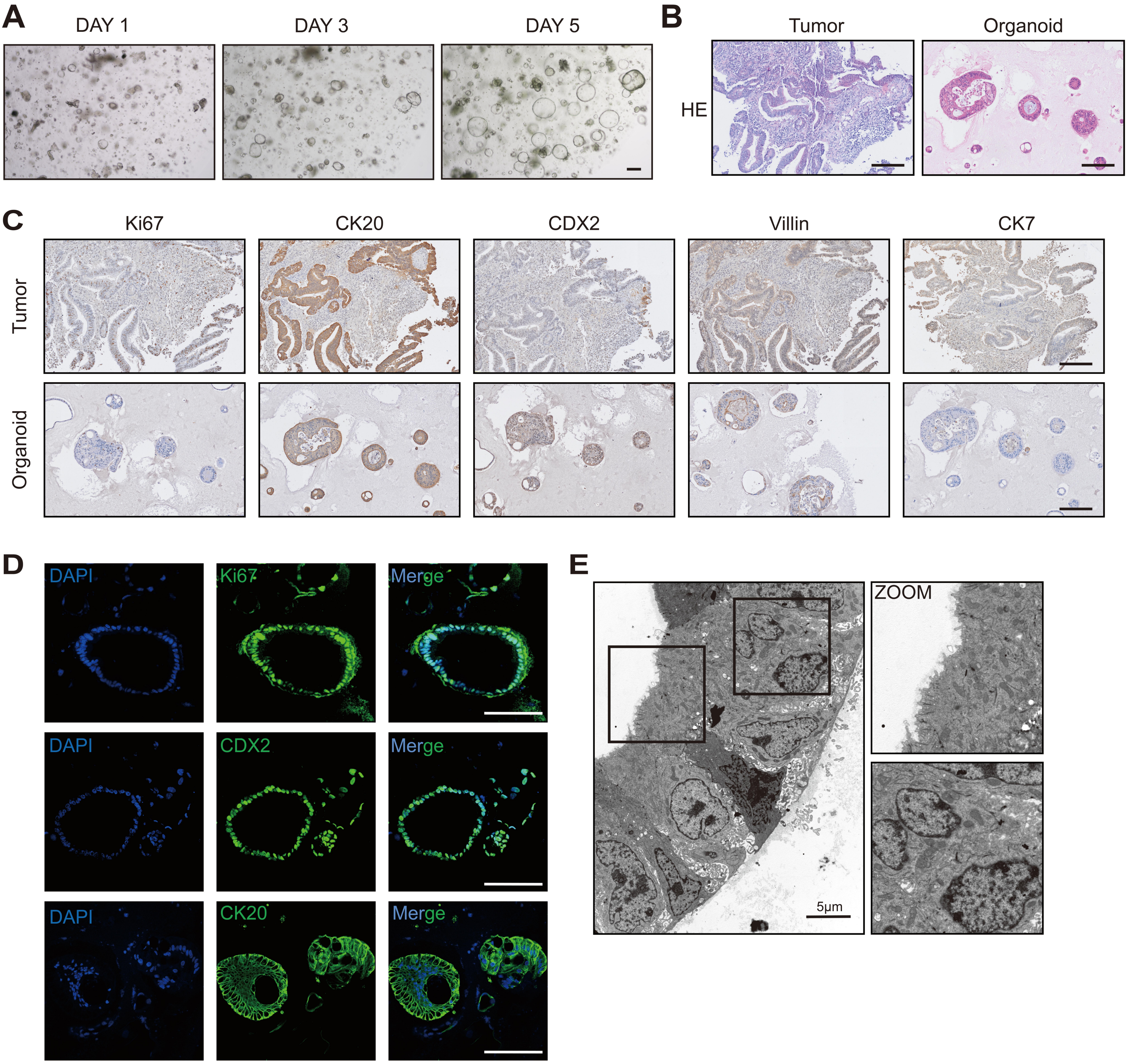
A
DAY 1
DAY 3
DAY 5
B
Tumor
Organoid
HE
C
Ki67
CK20
CDX2
Villin
CK7
Tumor
Organoid
D
DAPI
Ki67
Merge
DAPI
CDX2
Merge
DAPI
CK20
Merge
E
ZOOM
5μm

Table S1. GO enrichment analysis of serum starvation-specific EV proteins.

Category	Term	Description	Count	Percentage (%)	P value	Genes	Fold Enrichment
GOTERM_BP_DIRECT	GO:0007015	actin filament organization	3	11.11	0.02	MARCKS, MYO6, RHOF	14.60
GOTERM_BP_DIRECT	GO:0045742	positive regulation of epidermal growth factor receptor signaling pathway	2	7.41	0.04	RALA, RALB	52.34
GOTERM_BP_DIRECT	GO:0006915	apoptotic process	4	14.81	0.04	MARCKS, RALB, CD47, S100A9	5.12
GOTERM_BP_DIRECT	GO:0043542	endothelial cell migration	2	7.41	0.04	S100A9, FSTL1	42.17
GOTERM_BP_DIRECT	GO:0051603	proteolysis involved in protein catabolic process	2	7.41	0.06	PSMB7, CTSZ	33.73
GOTERM_BP_DIRECT	GO:0031623	receptor internalization	2	7.41	0.06	RALA, RALB	29.19
GOTERM_BP_DIRECT	GO:0098869	cellular oxidant detoxification	2	7.41	0.09	GSTO1, S100A9	20.51
GOTERM_BP_DIRECT	GO:0006954	inflammatory response	3	11.11	0.10	CD47, LYZ, S100A9	5.40
GOTERM_CC_DIRECT	GO:0070062	extracellular exosome	19	70.37	0.00	RALA, GRN, RALB, GSTO1, CTSZ, TACSTD2, TALDO1, RHOF, LYZ, FSTL1, CST3, MARCKS, S100A16, PIP, MYO6, CD47, DIP2B, S100A9, DSC1	6.51
GOTERM_CC_DIRECT	GO:0005576	extracellular region	10	37.04	0.00	CST3, PSMB7, GRN, CDSN, DNAH5, PIP, CTSZ, LYZ, S100A9, FSTL1	3.68

**GOTERM_CC_DIRECT**	**GO:0005886**	**plasma membrane**	**16**	**59.26**	**0.00**	**RALA, GRN, RALB, CDSN, PCDHGC4, CTSZ, RHOF, CST3, MARCKS, S100A16, MYO6, CD47, S100A9, DSC1, JAK1, SLC29A2**	**2.21**
**GOTERM_CC_DIRECT**	**GO:0005615**	**extracellular space**	**9**	**33.33**	**0.00**	**CST3, GRN, S100A16, PIP, CTSZ, TACSTD2, LYZ, S100A9, FSTL1**	**3.42**
**GOTERM_CC_DIRECT**	**GO:1904813**	**ficolin-1-rich granule lumen**	**3**	**11.11**	**0.01**	**CST3, PSMB7, CTSZ**	**18.57**
**GOTERM_CC_DIRECT**	**GO:0005938**	**cell cortex**	**3**	**11.11**	**0.02**	**MARCKS, CTSZ, MYO6**	**13.90**
**GOTERM_CC_DIRECT**	**GO:0030057**	**desmosome**	**2**	**7.41**	**0.03**	**CDSN, DSC1**	**55.26**
**GOTERM_CC_DIRECT**	**GO:0005788**	**endoplasmic reticulum lumen**	**3**	**11.11**	**0.06**	**CST3, CTSZ, FSTL1**	**7.46**
**GOTERM_CC_DIRECT**	**GO:0005768**	**endosome**	**3**	**11.11**	**0.06**	**GRN, MYO6, JAK1**	**6.97**
**GOTERM_CC_DIRECT**	**GO:1904724**	**tertiary granule lumen**	**2**	**7.41**	**0.07**	**CST3, LYZ**	**27.63**
**GOTERM_CC_DIRECT**	**GO:0001533**	**cornified envelope**	**2**	**7.41**	**0.07**	**CDSN, DSC1**	**25.79**
**GOTERM_CC_DIRECT**	**GO:0035580**	**specific granule lumen**	**2**	**7.41**	**0.08**	**CTSZ, LYZ**	**24.56**
**GOTERM_CC_DIRECT**	**GO:0030139**	**endocytic vesicle**	**2**	**7.41**	**0.08**	**RALA, MYO6**	**22.75**
**GOTERM_MF_DIRECT**	**GO:0005509**	**calcium ion binding**	**5**	**18.52**	**0.02**	**S100A16, PCDHGC4, S100A9, DSC1, FSTL1**	**4.79**

**GOTERM_MF_DIRECT**	**GO:0003925**	**G protein activity**	**2**	**7.41**	**0.07**	**RALA, RALB**	**28.60**
**GOTERM_MF_DIRECT**	**GO:0031625**	**ubiquitin protein ligase binding**	**3**	**11.11**	**0.07**	**RALA, RALB, JAK1**	**6.90**
**GOTERM_MF_DIRECT**	**GO:0003924**	**GTPase activity**	**3**	**11.11**	**0.08**	**RALA, RALB, RHOF**	**6.02**
**GOTERM_MF_DIRECT**	**GO:0019003**	**GDP binding**	**2**	**7.41**	**0.10**	**RALA, RALB**	**18.57**

Table S2. GSEA results for vesicle membrane-related gene sets.

	Symbol	Rank in Gene List	Rank Metric Score	Core Enrichment
1	ANXA3	0	4.106	Yes
2	B2M	1	3.936	Yes
3	CD58	12	2.558	Yes
4	ANXA6	18	2.147	Yes
5	HLA-A	20	2.109	Yes
6	CD55	25	1.815	Yes
7	PCSK9	31	1.703	Yes
8	ANXA1	36	1.519	Yes
9	CLIC4	39	1.438	Yes
10	RAP1A	41	1.434	Yes
11	HLA-C	50	1.333	Yes
12	VNN1	51	1.316	Yes
13	GPRC5A	52	1.312	Yes
14	ITGB1	53	1.31	Yes
15	CD109	55	1.239	Yes
16	CD46	56	1.232	Yes
17	BSG	57	1.225	Yes
18	SLC39A14	68	1.095	Yes
19	ATP2B1	69	1.093	Yes
20	CD59	70	1.081	Yes
21	ANXA4	73	1.036	Yes
22	RAB35	80	0.989	Yes
23	FOLR1	85	0.939	Yes
24	DSG1	90	0.913	Yes
25	RHOG	92	0.91	Yes
26	CD9	95	0.893	Yes
27	CD44	99	0.862	Yes
28	SCARB1	104	0.832	Yes
29	ANPEP	105	0.822	Yes
30	ADAM10	107	0.807	Yes
31	SLC30A1	108	0.802	Yes
32	CD63	117	0.749	Yes
33	MYO1C	123	0.682	Yes
34	RAC1	127	0.672	Yes
35	RAB1A	128	0.668	Yes
36	RHOA	134	0.639	Yes
37	RAB10	137	0.631	Yes
38	MYOF	144	0.611	No
39	BST2	149	0.582	No
40	STOM	152	0.564	No
41	ANXA7	176	0.402	No
42	VPS4A	199	0.253	No
43	TFRC	212	0.175	No
44	HSPA8	219	0.145	No
45	RAB7A	223	0.089	No

46	EHD2	237	-0.017	No
47	IQGAP1	240	-0.063	No
48	PSAP	245	-0.083	No
49	ANXA2	252	-0.148	No
50	EHD3	254	-0.157	No
51	ITGAV	264	-0.214	No
52	PDCD6	268	-0.249	No
53	LRP1	310	-0.446	No
54	CALR	325	-0.54	No
55	SCARB2	326	-0.546	No
56	CTSD	340	-0.61	No
57	LAMP2	344	-0.656	No
58	STX4	351	-0.703	No
59	PDIA3	353	-0.713	No
60	SRI	369	-0.836	No
61	IGF2R	375	-0.858	No
62	FABP5	378	-0.874	No
63	SPIRE2	407	-1.172	No
64	APOE	424	-1.42	No
65	CLTC	433	-1.708	No
66	DSP	434	-1.748	No

Table S3 key resources table

REAGENT or RESOURCE	SOURCE	IDENTIFIER
**Antibodies**		
Rabbit monoclonal anti-human LC3B	Cell Signaling Technology	Cat#3868S; RRID:AB_2137707
Mouse monoclonal anti-human CD63	GeneTex	Cat#GTX28219; RRID:AB_374496
Rabbit Polyclonal anti-human CD63	Proteintech	Cat#25682-1-AP; RRID:AB_2783831
Rabbit monoclonal anti-human LAMP1	Cell Signaling Technology	Cat#9091; RRID:AB_2687579
Mouse monoclonal anti-human CD9	Proteintech	Cat#60232-1-Ig; RRID:AB_11232215
Mouse monoclonal anti-human CD81	Proteintech	Cat#66866-1-Ig; RRID:AB_2882203
Rabbit Polyclonal anti-human TSG101	Proteintech	Cat#28283-1-AP; RRID:AB_2881104
Rabbit Polyclonal anti-human Calnexin	Proteintech	Cat#10427-2-AP; RRID:AB_2069033
Rabbit Polyclonal anti-human Cytokeratin 20	Proteintech	Cat#17329-1-AP; RRID:AB_2133592
Rabbit Polyclonal anti-human Ki-67	Proteintech	Cat#27309-1-AP; RRID:AB_2756525
Rabbit Polyclonal anti-human CDX2	Proteintech	Cat#22101-1-AP; RRID:AB_3085684
Rabbit Polyclonal anti-human PCNA	Proteintech	Cat#10205-2-AP; RRID:AB_2160330
Rabbit Polyclonal anti-human Caspase3	Proteintech	Cat#25128-1-AP; RRID:AB_3073913
Rabbit Polyclonal anti-human ATG3	Proteintech	Cat#11262-2-AP; RRID:AB_2059234
Rabbit Polyclonal anti-human CHMP4B	Proteintech	Cat#13683-1-AP; RRID:AB_2877971
Rabbit monoclonal anti-human ALIX	Cell Signaling Technology	Cat#92880; RRID:AB_2800192
Rabbit monoclonal anti-human HRS	Cell Signaling Technology	Cat#15087; RRID:AB_2798700
Mouse monoclonal anti-human EEA1	Cell Signaling Technology	Cat#48453; RRID:AB_2920538
Rabbit Polyclonal anti-human ANXA3	Proteintech	Cat#11804-1-AP; RRID:AB_2057455
Rabbit Polyclonal anti-human ANXA3	ThermoFisher	Cat#PA5-78780; RRID:AB_2745896
Mouse monoclonal anti-human β-actin	Abbkine	Cat#A01010; RRID:AB_2737288
HRP, Goat Anti-Rabbit IgG	Abbkine	Cat#A21020; RRID:AB_2876889
HRP, Goat Anti-Mouse IgG	Abbkine	Cat#A21010; RRID:AB_2728771
Goat Anti-Rabbit IgG/Gold 10nm	Solarbio	Cat#K1034G-G10
Goat Anti-Mouse IgG/Gold 10nm	Solarbio	Cat#K1031G-G10
Dylight 594, Goat Anti-Rabbit IgG	Abbkine	Cat#A23420;
Dylight 594, Goat Anti-Mouse IgG	Abbkine	Cat#A23410; RRID:AB_2939057
DyLight 488, Goat Anti-Mouse IgG	Abbkine	Cat#A23210; RRID:AB_2923050
DyLight 488, Goat Anti-Rabbit IgG	Abbkine	Cat#A23220; RRID:AB_2737289
His-Tag Rabbit mAb	Cell Signaling Technology	Cat#12698S; RRID:AB_2744546
Myc-Tag Mouse mAb	Cell Signaling Technology	Cat#2276S; RRID:AB_331783
**Biological samples**		
Human colorectal cancer tissues	The Fourth Affiliated Hospital of China Medical University	N/A
**Chemicals, peptides, and recombinant proteins**		
DMEM	Procell	PM150210
MEM	Procell	PM150410
McCoy's 5A	Procell	PM150710
Fetal Bovine Serum	Cell-box	164210
Exosome-Free FBS	NovaCells	F002P
Penicillin-Streptomycin Solution	Procell	PB180120
Trypsin-EDTA Solution	Abbkine	BMU109
RIPA Lysis Buffer	EpiZyme	PC103
Protein Sample Loading Buffer	EpiZyme	LT103
Tris-Glycine Electrophoresis Buffer	Servicebio	G2152-1L

Western Transfer Buffer	Servicebio	G2154-1L
Western Blocking Buffer	EpiZyme	PS108P
Opti-Protein Ultra Marker	Abm	G623
Bovine Serum Albumin	Absin	abs49001014
DiD Perchlorate	Absin	abs47014947
AbFluor™ 488-Phalloidin	Abbkine	BMD0082
DAPI solution	Solarbio	C0065
Mounting Medium, Antifading	Solarbio	S2110
Lipofectamine 3000	Invitrogen	L3000015
Colorectal Cancer Organoid Culture Medium	Organpharma	NGH020001
Primary Tissue Storage Solution	Organpharma	NGH030020
Tissue Digestion Solution	Organpharma	NGH030021
Organoid Cryopreservation Medium	Organpharma	NGH030023
Extracellular Matrlx	Organpharma	NGH030024
**Critical commercial assays**		
PAGE Gel Quick Preparation Kit	EpiZyme	PG112
In situ PLA kit	Duolink	DUO92101
Annexin V-FITC/PI Apoptosis Kit	Abbkine	KTA0002
Cell Counting Kit-8	Abbkine	BMU106
Protein Quantification Kit	Abbkine	KTD3001
West Femto Maximum Sensitivity Substrate	Abbkine	BMU102
Protein A/G Magnetic Beads	Biolinkedin	L-1004
**Experimental models: Cell lines**		
Hela	Zhong Qiao Xin Zhou Biotechnology	Cat#ZQ0068; RRID: CVCL_0030
Huh7	Zhong Qiao Xin Zhou Biotechnology	Cat#ZQ0025; RRID: CVCL_0336
HCT116	Zhong Qiao Xin Zhou Biotechnology	Cat#ZQ0125; RRID: CVCL_0291
HcerEpic	Otwo Biotechnology	Cat#HTX2613
THLE-2	Zhejiang Noble Biological Products Co.,Ltd	Cat#nobcell0548; RRID: CVCL_3803
NCM460	Hunan Fenghui Biotechnology Co., Ltd	Cat#CL0393; RRID: CVCL_0460
**Experimental models: Organisms/strains**		
BALB/cA-nu Mice	BEIJING HFK BIOSCIENCE	13001A
**Oligonucleotides**		
Human HRS siRNA 1: CCAUCAAGAAGAAAGUCAATT	This paper	
Human HRS siRNA 2: GGAACGAGCCCAAGUACAATT	This paper	
Human HRS siRNA 3: GCAUGAAGAGUAACCACAUTT	This paper	
Human ALIX siRNA 1: GAGACGCUCCUGAGAUAUUTT	This paper	
Human ALIX siRNA 2: GCUUGACAUUUACCUGGAATT	This paper	
Human ALIX siRNA 3:	This paper	

GGAAGGAUGCUUUCGAUAATT		
Human ANXA3 sgRNA 1: TCCAGACTTTAGCCCATCAG	This paper	
Human ANXA3 sgRNA 2: TCATCAGCATTCTGACTGAG	This paper	
Human ANXA3 sgRNA 3: TTTGCATCAAAGACTGCTGG	This paper	
Human ATG3 shRNA : TGTCATTCCAACAATAGAA	This paper	
Human ALIX sgRNA 1: AAAGACCTCAGAGGTGGACC	This paper	
Human ALIX sgRNA 2: TGCTGGATGAACTTCACCAG	This paper	
Human ALIX sgRNA 3: CGTCCGCTGGACAAGCACGA	This paper	
**Recombinant DNA**		
EGFP-Rab5A Q79L	Addgene	#28046
GFP-CD63	Genechem	N/A
RFP-GFP-LC3B	Genechem	N/A
ALIX Δ PRR-Cherry	This paper	N/A
Myc-HRS plasmid	This paper	N/A
His-ALIX plasmid	This paper	N/A
**Software and algorithms**		
Graphpad Prism 8.0.1	Graphpad	https://www.graphpad.com/
ImageJ 1.53	ImageJ	https://ImageJ.nih.gov
FlowJo_v10.6.2	FlowJo	https://www.flowjo.com/
NIS-Elements Viewer 5.21	NIS	https://www.microscope.healthcare.nikon.com/
NanoViewer 2.8.10	NanoViewer	N/A

Table S4.Specific concentrations of antibodies used in Western blot

Antibody	Source	Dilution
Rabbit monoclonal anti-human LC3B	Cell Signaling Technology	1:1000
Mouse monoclonal anti-human CD63	GeneTex	1:1000
Rabbit Polyclonal anti-human CD63	Proteintech	1:1000
Rabbit monoclonal anti-human LAMP1	Cell Signaling Technology	1:1000
Mouse monoclonal anti-human CD9	Proteintech	1:10000
Mouse monoclonal anti-human CD81	Proteintech	1:3000
Rabbit Polyclonal anti-human TSG101	Proteintech	1:5000
Rabbit Polyclonal anti-human Calnexin	Proteintech	1:10000
Rabbit Polyclonal anti-human ATG3	Proteintech	1:5000
Rabbit Polyclonal anti-human CHMP4B	Proteintech	1:5000
Rabbit monoclonal anti-human ALIX	Cell Signaling Technology	1:1000
Rabbit monoclonal anti-human HRS	Cell Signaling Technology	1:1000
Rabbit Polyclonal anti-human ANXA3	Proteintech	1:3000
Rabbit Polyclonal anti-human ANXA3	ThermoFisher	1:1000
Mouse monoclonal anti-human β-actin	Abbkine	1:10000

HRP, Goat Anti-Rabbit IgG	Abbkine	1:10000
HRP, Goat Anti-Mouse IgG	Abbkine	1:10000
His-Tag Rabbit mAb	Cell Signaling Technology	1:1000
Myc-Tag Mouse mAb	Cell Signaling Technology	1:1000
