# Supplementary material for: Serum starvation drives ALIX-dependent extracellular vesicle biogenesis and determines tumor progression
Source: JCI Insight. 2026 Jun 8;11(11):e197924. doi: 10.1172/jci.insight.197924 (PMC13313550; doi:10.1172/jci.insight.197924)

Unedited gel

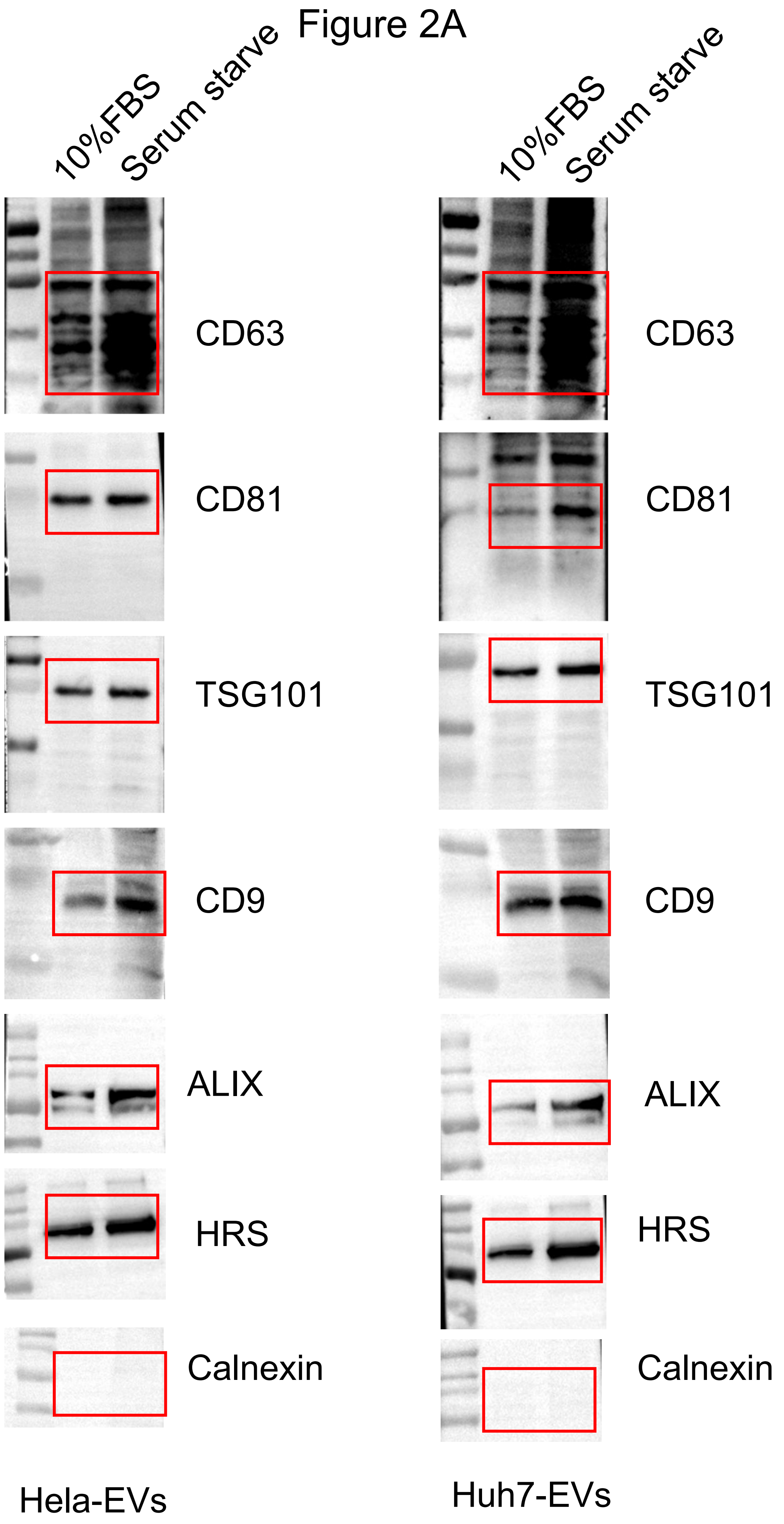

Figure 3F

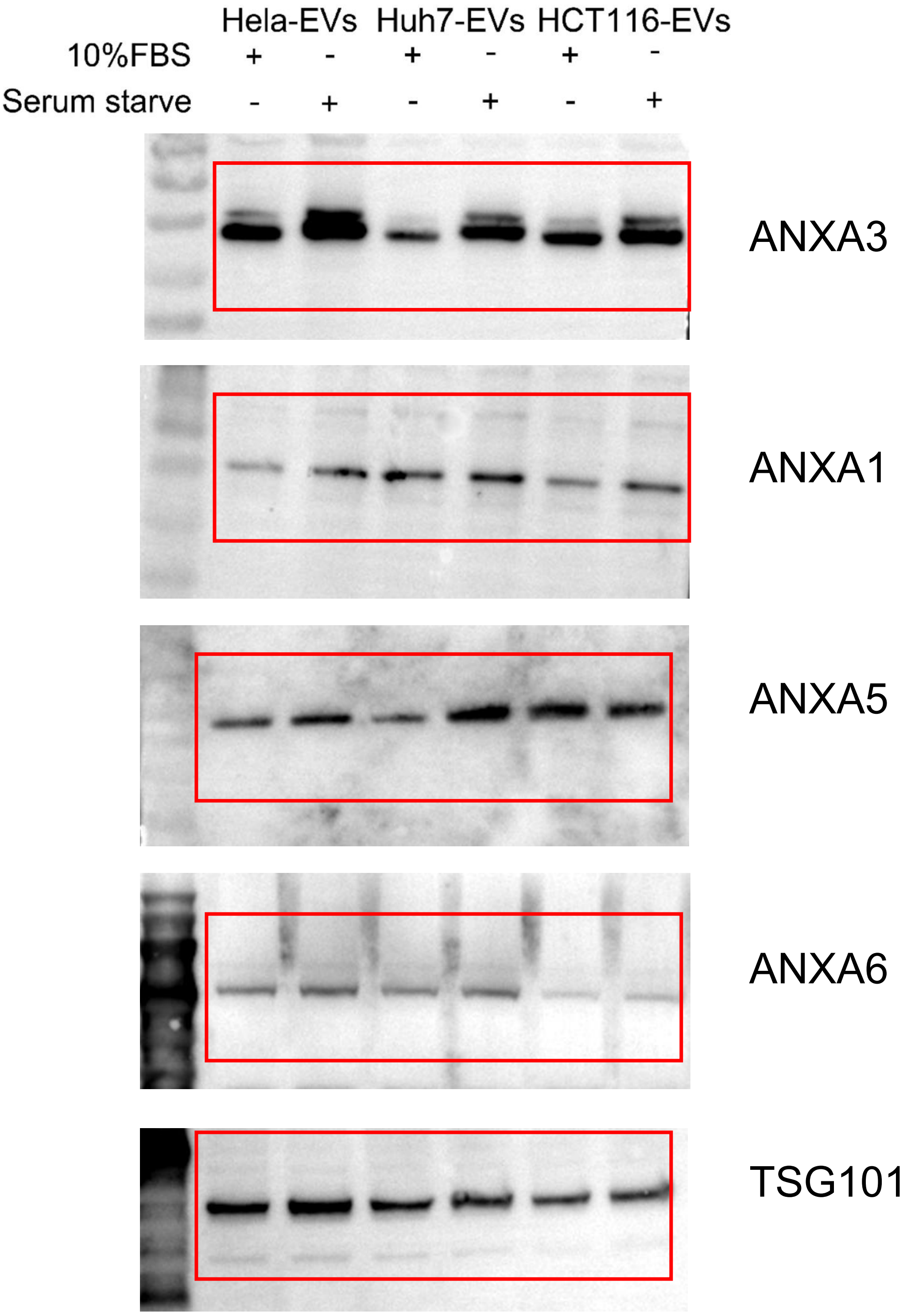

Hela-EVs
Huh7-EVs
HCT116-EVs
10%FBS + - + - + -
Serum starve - + - + - +
ANXA3
ANXA1
ANXA5
ANXA6
TSG101

Figure 4A

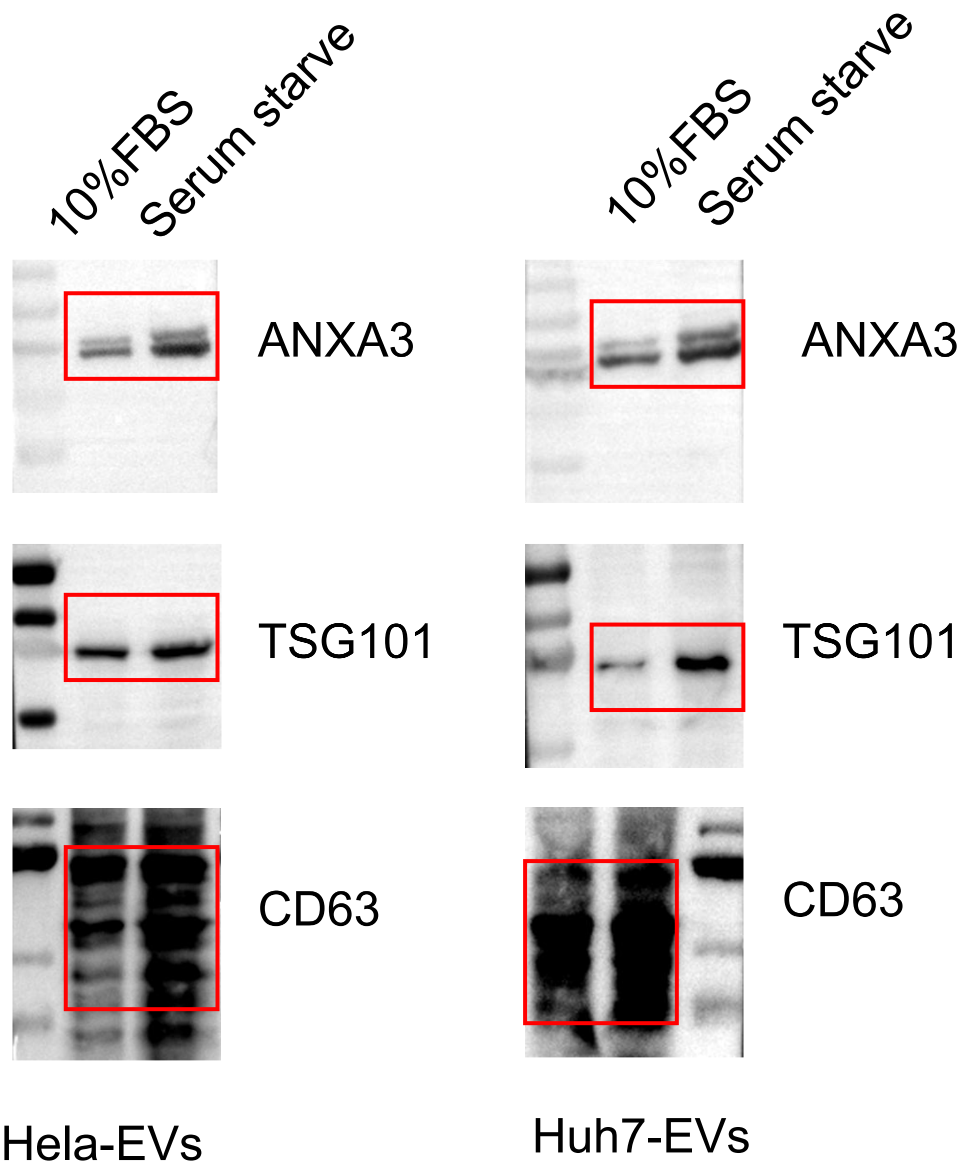
10%FBS
Serum starve
ANXA3
TSG101
CD63
Hela-EVs
10%FBS
Serum starve
ANXA3
TSG101
CD63
Huh7-EVs

Figure 5A

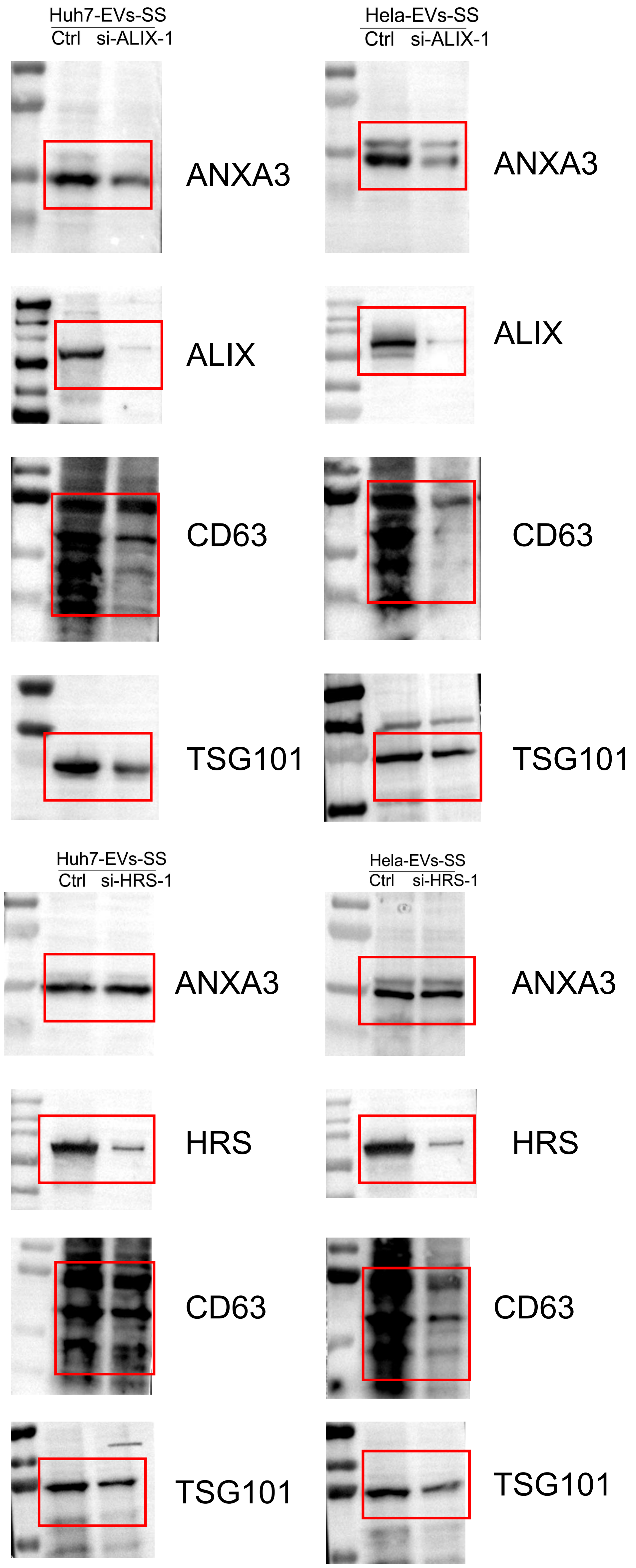
Huh7-EVs-SS
Ctrl si-ALIX-1
Hela-EVs-SS
Ctrl si-ALIX-1
ANXA3
ANXA3
ALIX
ALIX
CD63
CD63
TSG101
TSG101
Huh7-EVs-SS
Ctrl si-HRS-1
Hela-EVs-SS
Ctrl si-HRS-1
ANXA3
ANXA3
HRS
HRS
CD63
CD63
TSG101
TSG101

Figure 5G

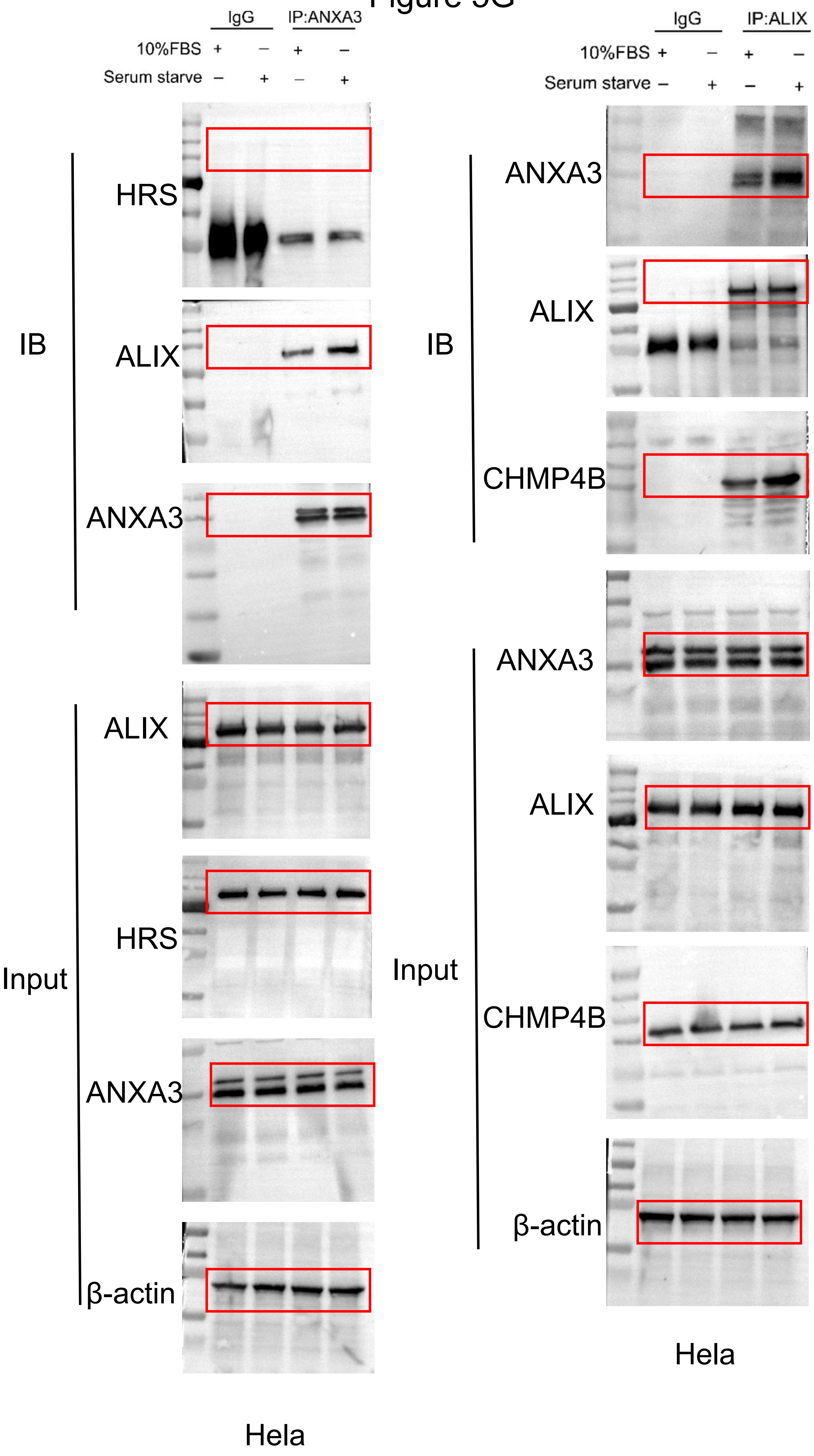
IgG
IP:ANXA3
10%FBS + – + –
Serum starve – + – +
IB
HRS
ALIX
ANXA3
Input
ALIX
HRS
ANXA3
β-actin
Hela
IgG
IP:ALIX
10%FBS + – + –
Serum starve – + – +
IB
ANXA3
ALIX
CHMP4B
Input
ANXA3
ALIX
CHMP4B
β-actin
Hela

Figure 5I

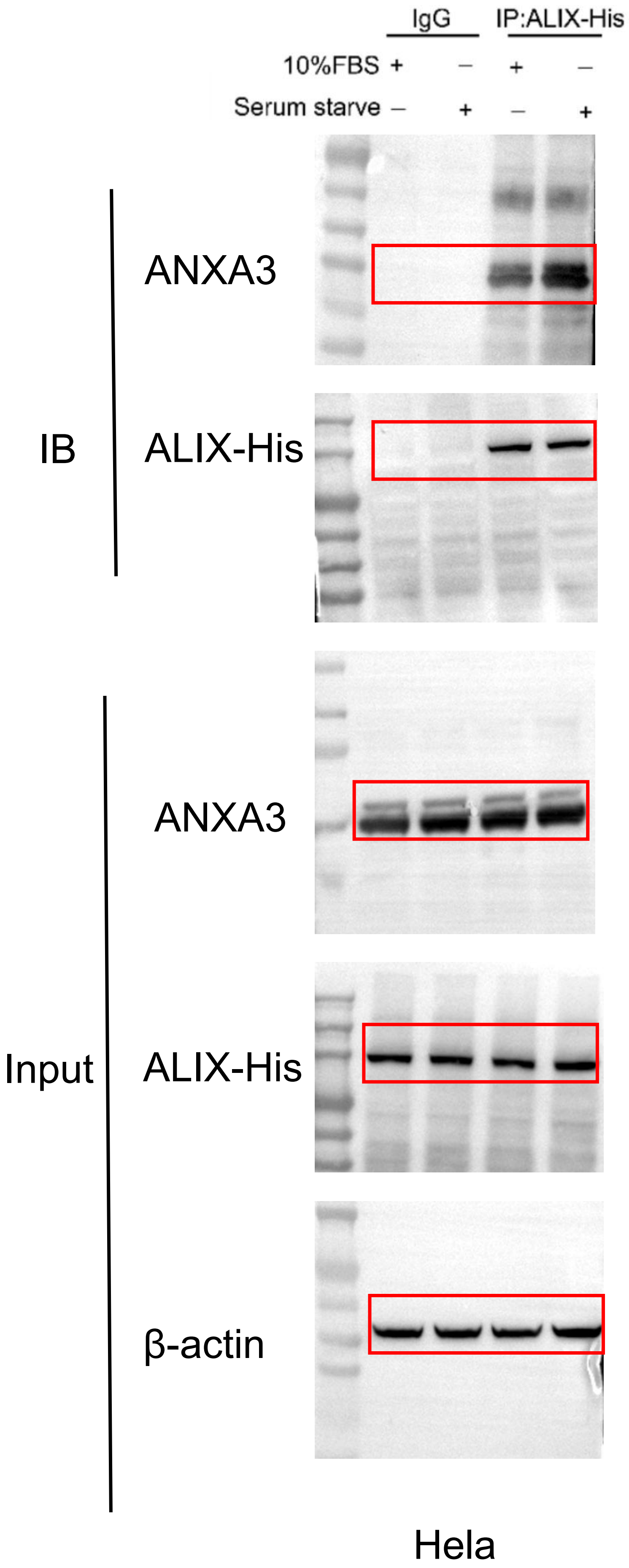
IgG
IP:ALIX-His
10%FBS + – + –
Serum starve – + – +
IB
ANXA3
ALIX-His
Input
ANXA3
ALIX-His
β-actin
Hela

Figure 6A

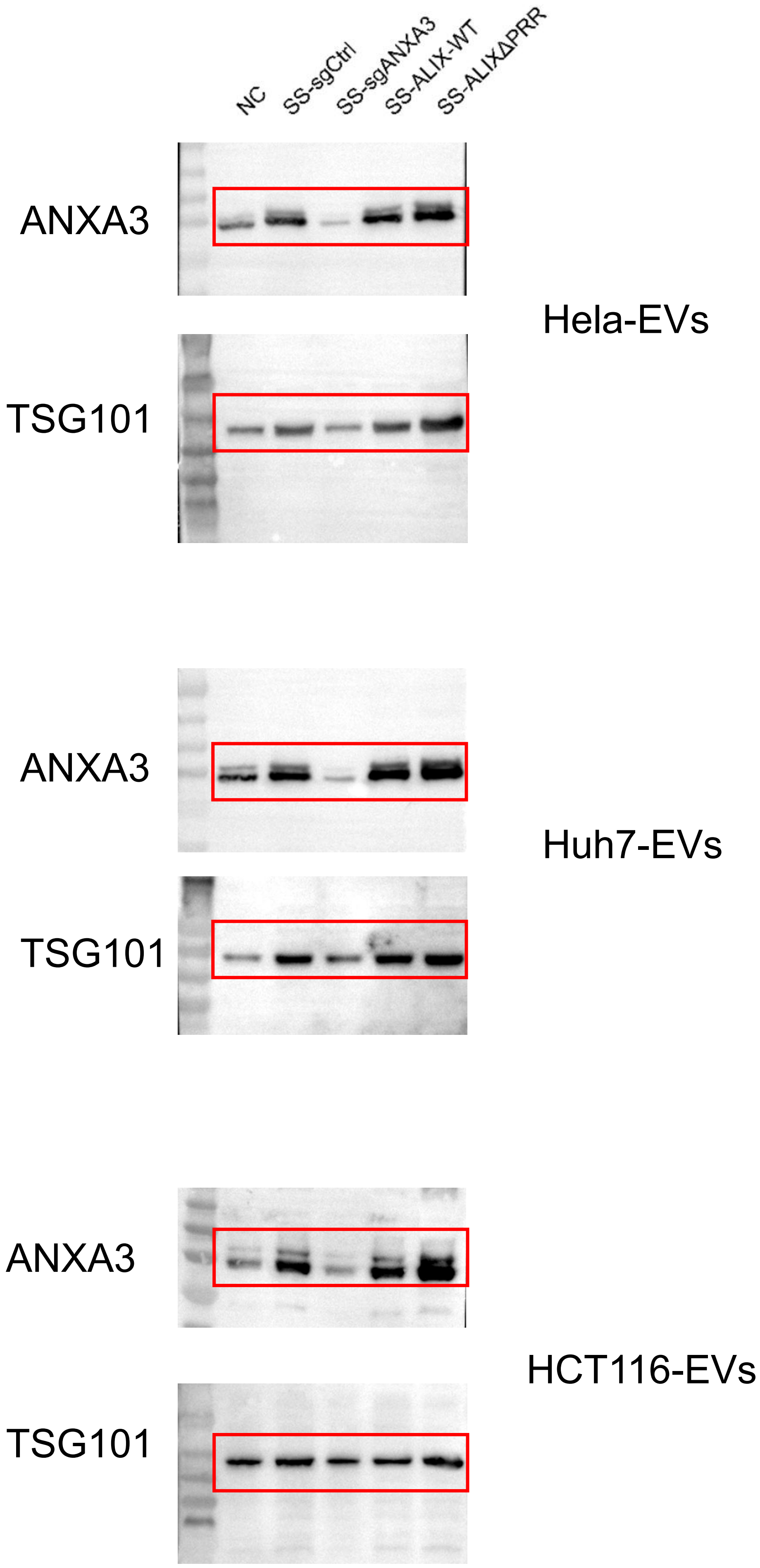

NC
SS-sgCtrl
SS-sgANXA3
SS-ALIX-WT
SS-ALIXΔPRR
ANXA3
TSG101
Hela-EVs
ANXA3
TSG101
Huh7-EVs
ANXA3
TSG101
HCT116-EVs

Figure S2A

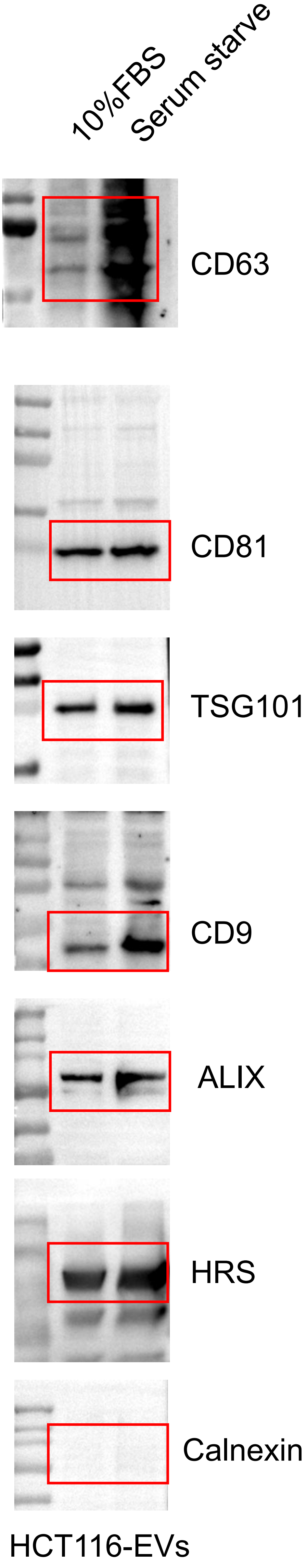

10%FBS
Serum starve
CD63
CD81
TSG101
CD9
ALIX
HRS
Calnexin
HCT116-EVs

Figure S3D

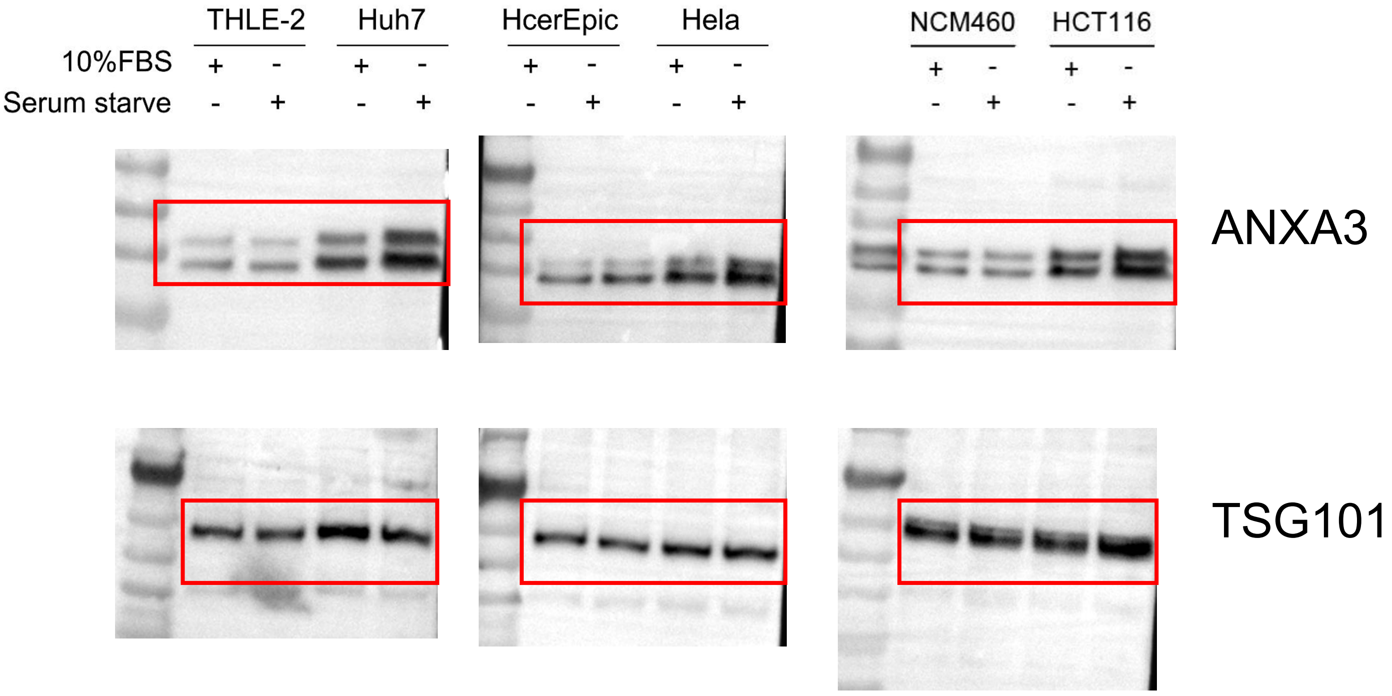
THLE-2
Huh7
HcerEpic
Hela
NCM460
HCT116
10%FBS + - + - + - + - + - + -
Serum starve - + - + - + - + - + - +
ANXA3
TSG101

Figure S4A

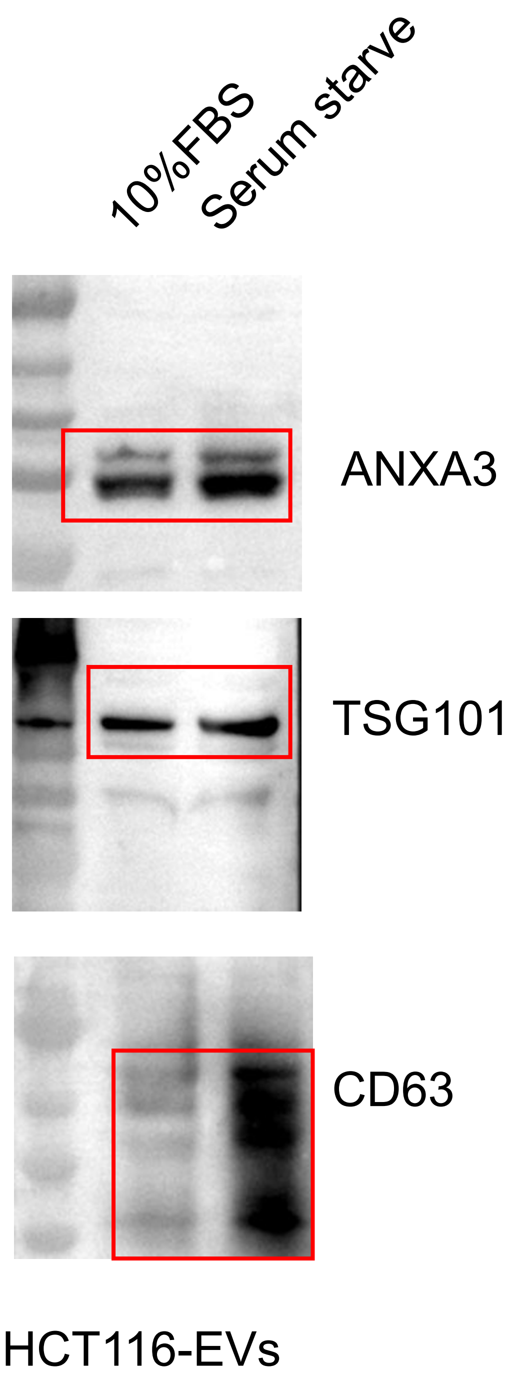

Figure S4C

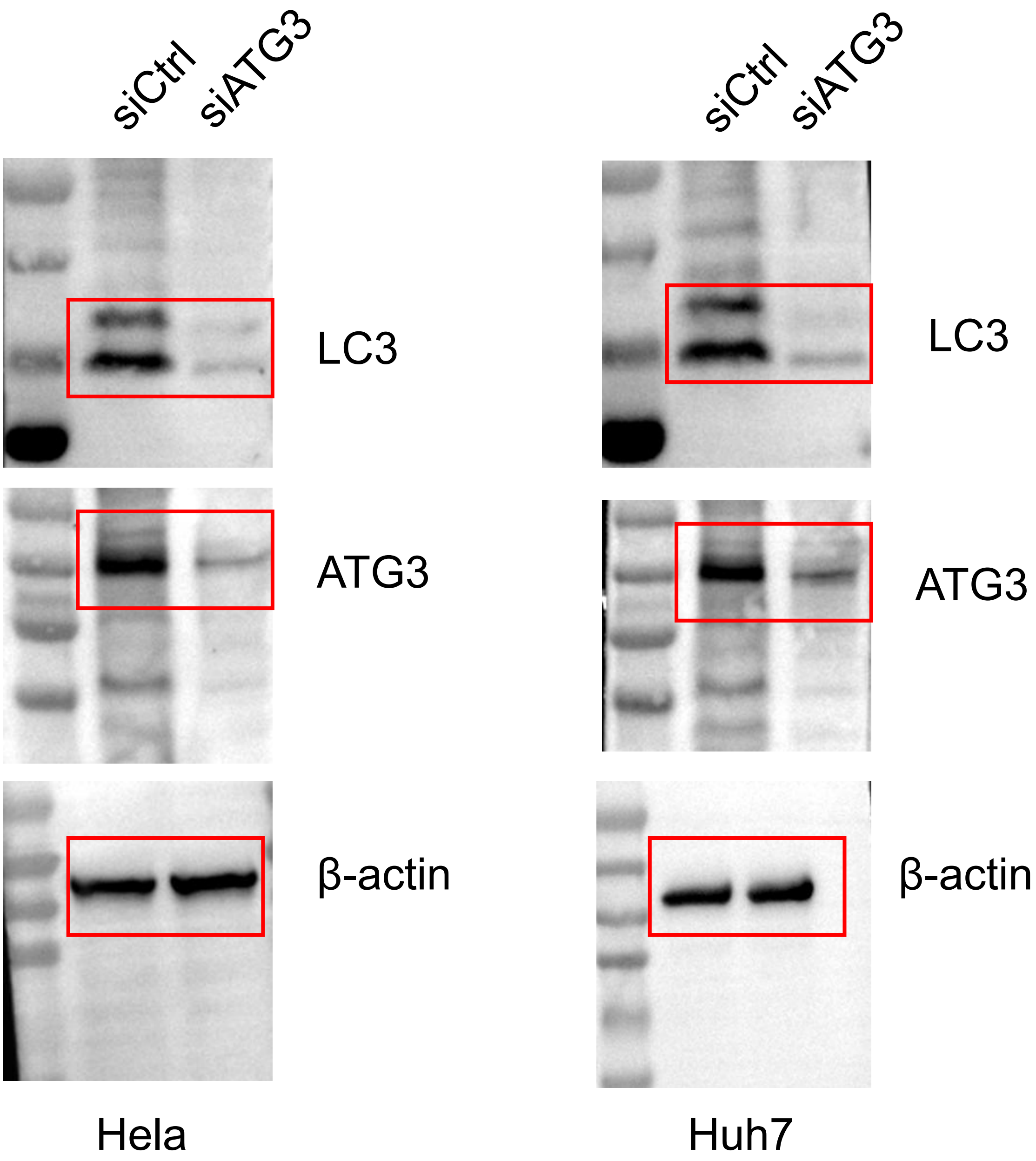
siCtrl
siATG3
LC3
ATG3
β-actin
Hela
siCtrl
siATG3
LC3
ATG3
β-actin
Huh7

Figure S4D

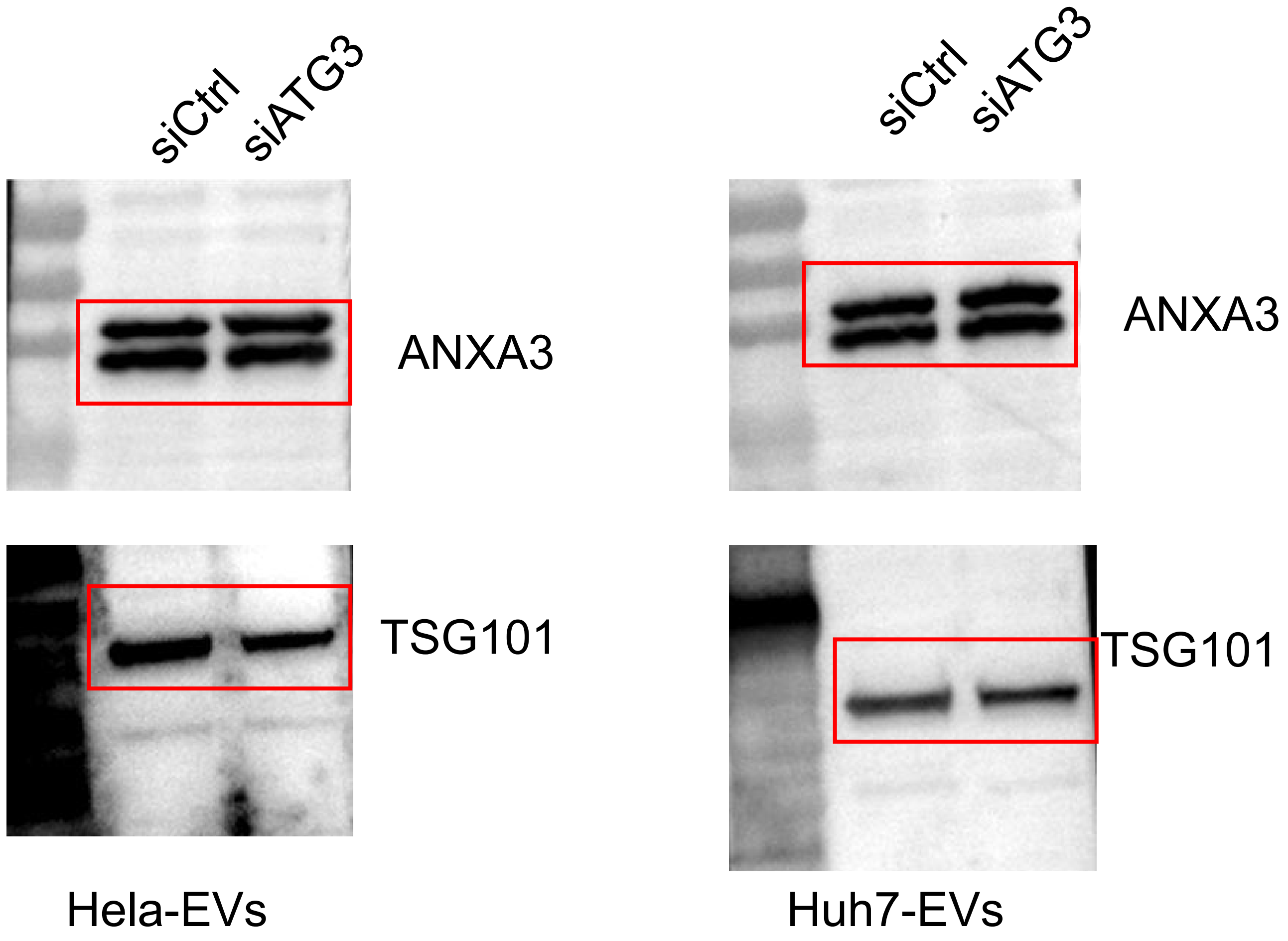
siCtrl
siATG3
ANXA3
TSG101
Hela-EVs
siCtrl
siATG3
ANXA3
TSG101
Huh7-EVs

Figure S5A

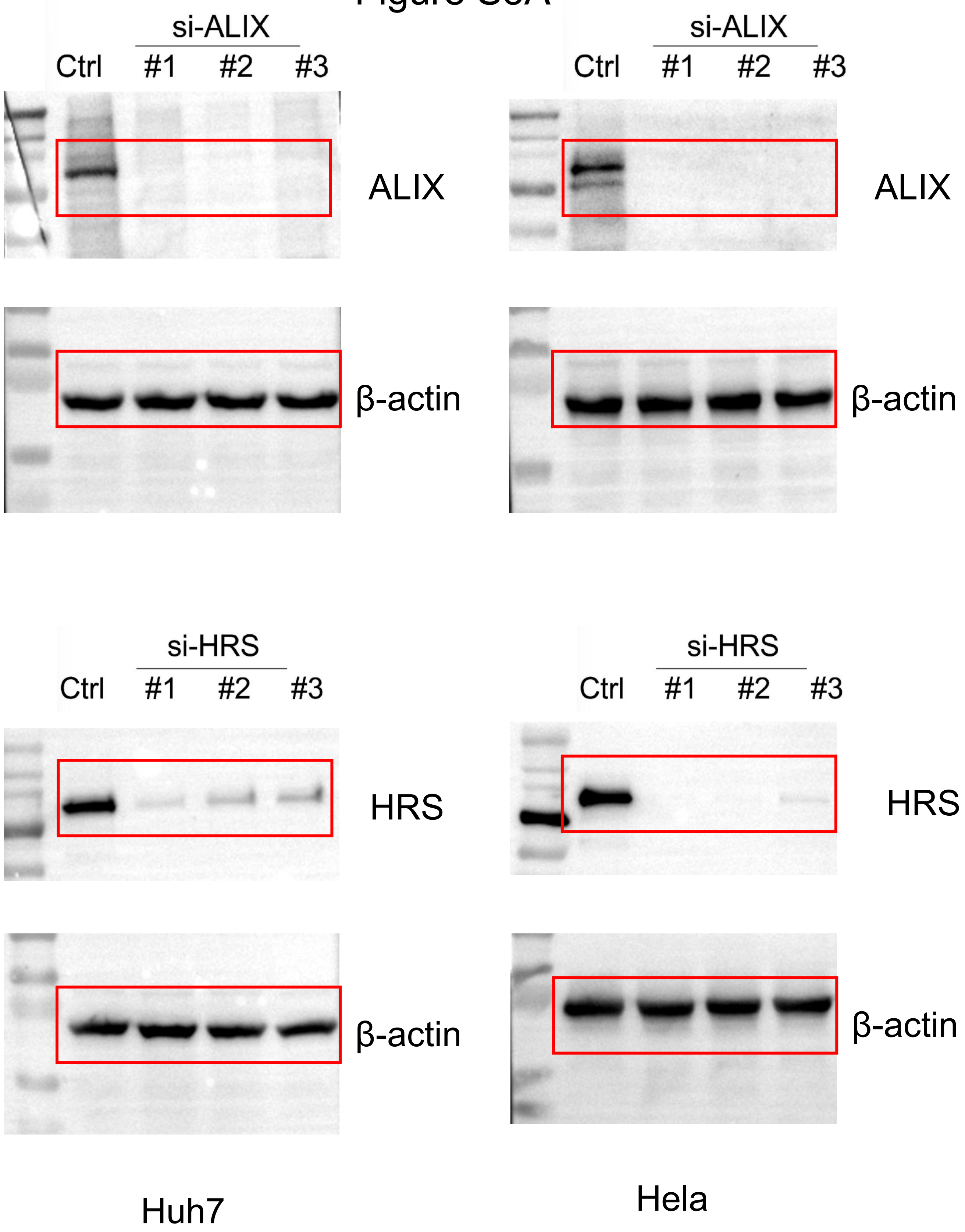
si-ALIX
Ctrl
#1
#2
#3
ALIX
β-actin
si-ALIX
Ctrl
#1
#2
#3
ALIX
β-actin
si-HRS
Ctrl
#1
#2
#3
HRS
β-actin
si-HRS
Ctrl
#1
#2
#3
HRS
β-actin
Huh7
Hela

Figure S5B

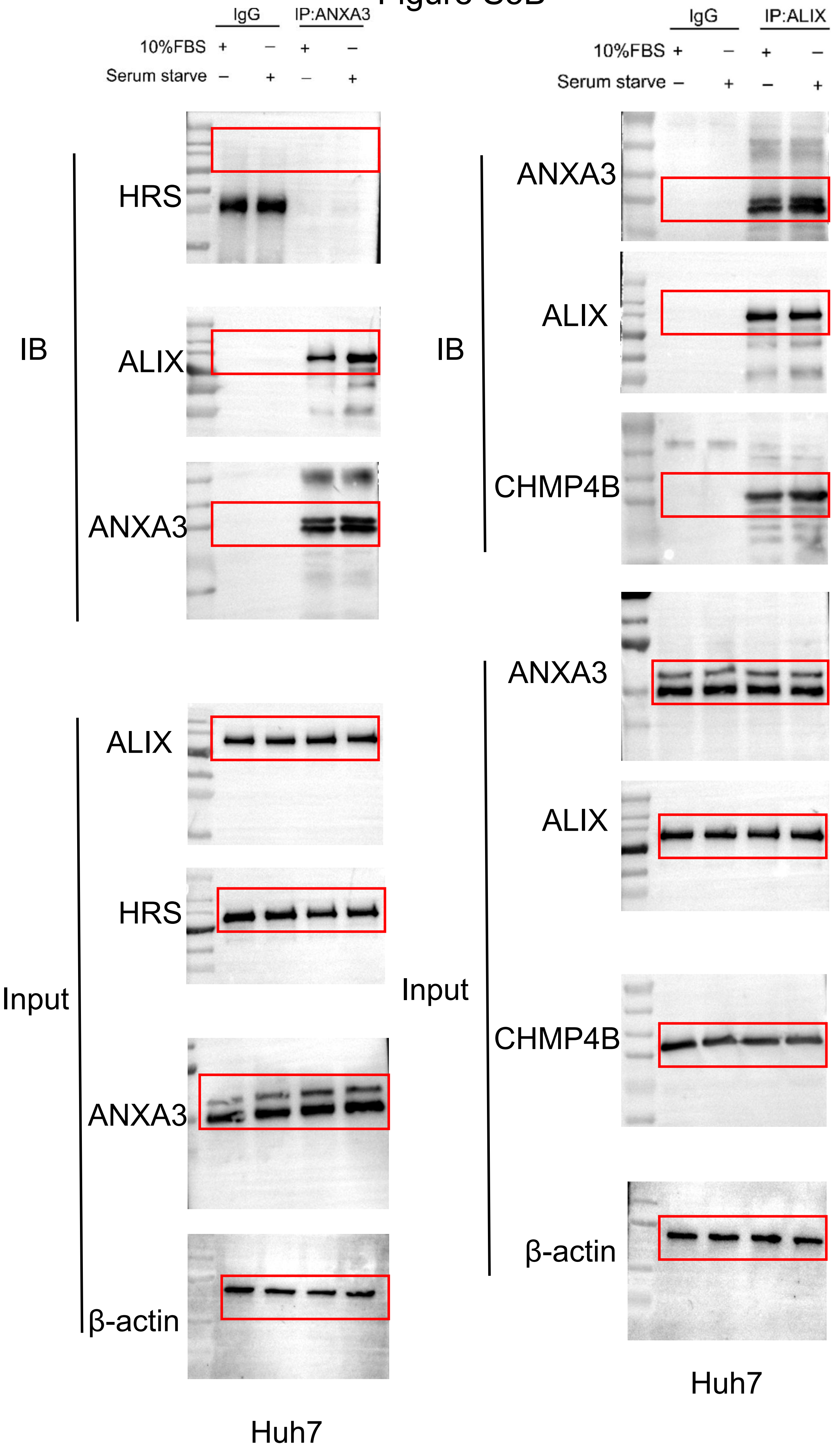
IgG
IP:ANXA3
10%FBS + – + –
Serum starve – + – +
IB
HRS
ALIX
ANXA3
Input
ALIX
HRS
ANXA3
β-actin
Huh7
IgG
IP:ALIX
10%FBS + – + –
Serum starve – + – +
IB
ANXA3
ALIX
CHMP4B
Input
ANXA3
ALIX
CHMP4B
β-actin
Huh7

Figure S5D

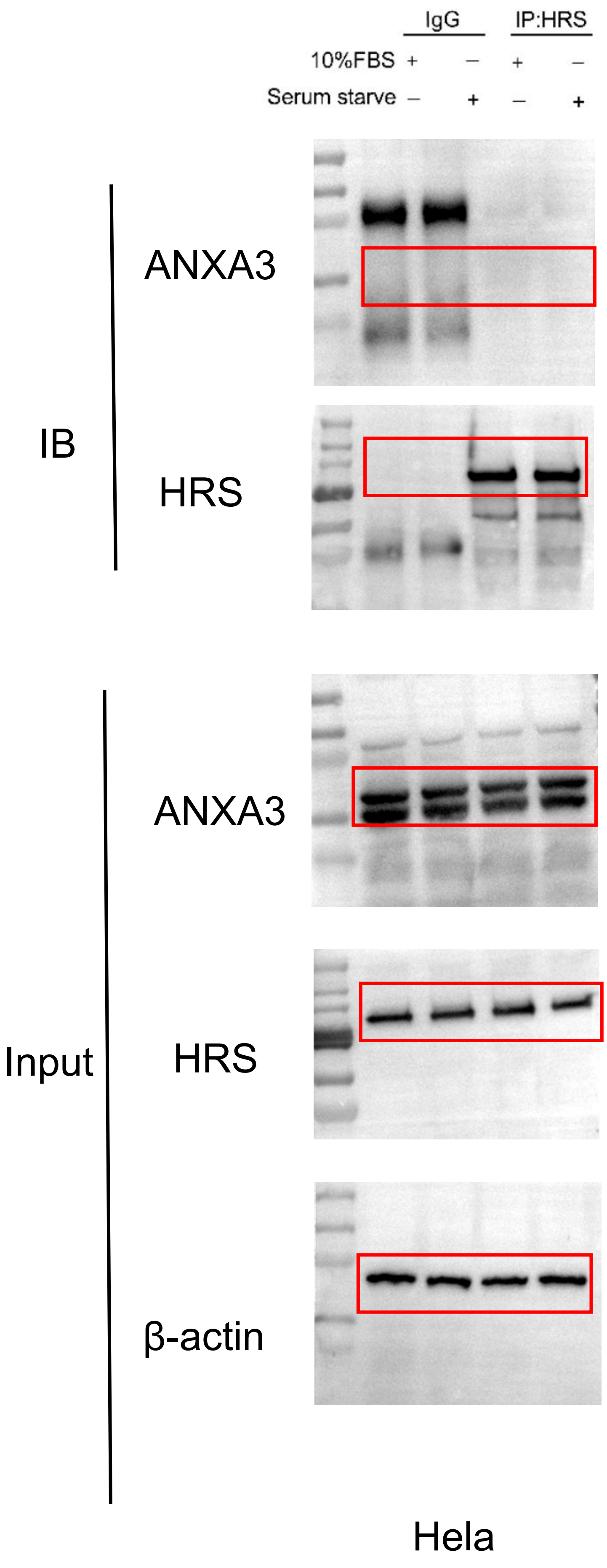
IgG
IP:HRS
10%FBS + – + –
Serum starve – + – +
IB
ANXA3
HRS
Input
ANXA3
HRS
β-actin
Hela

Figure S5E

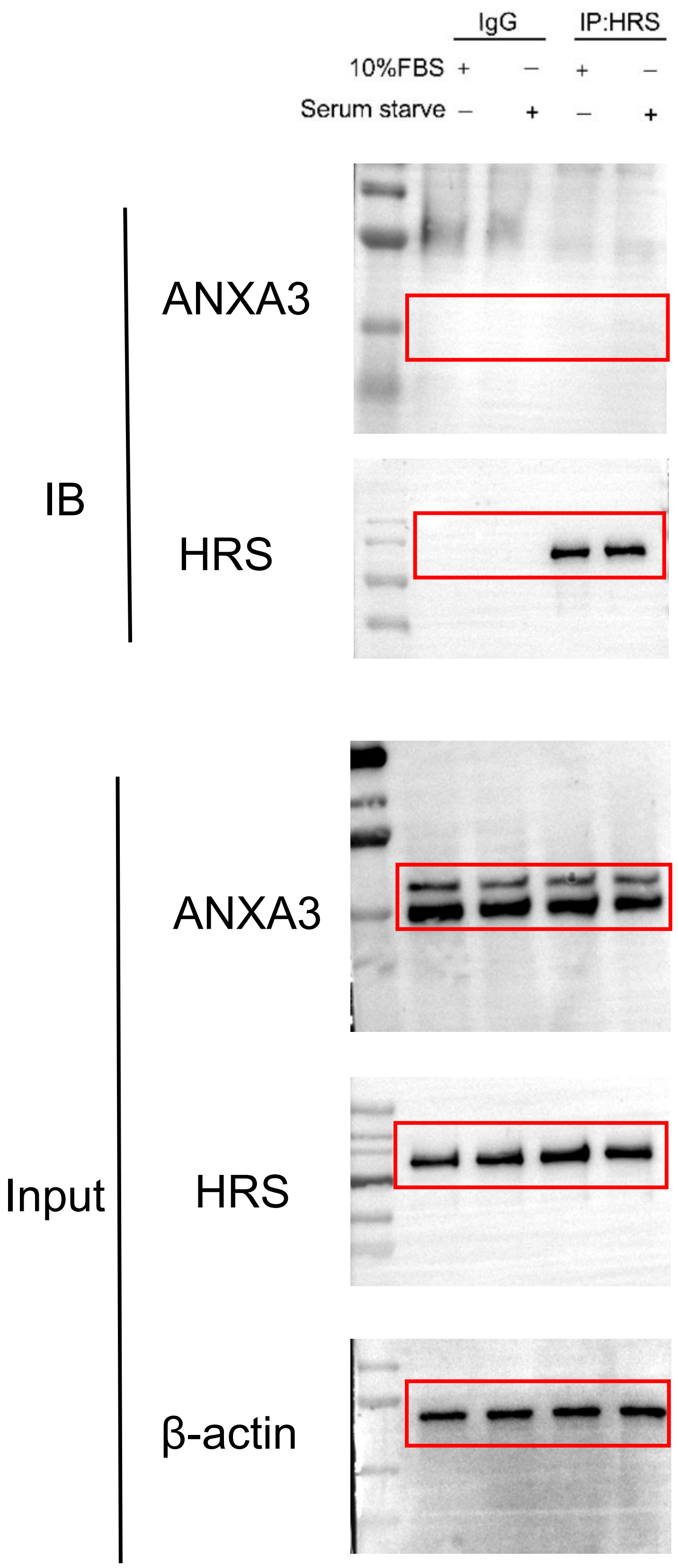

Huh7

Figure S5F

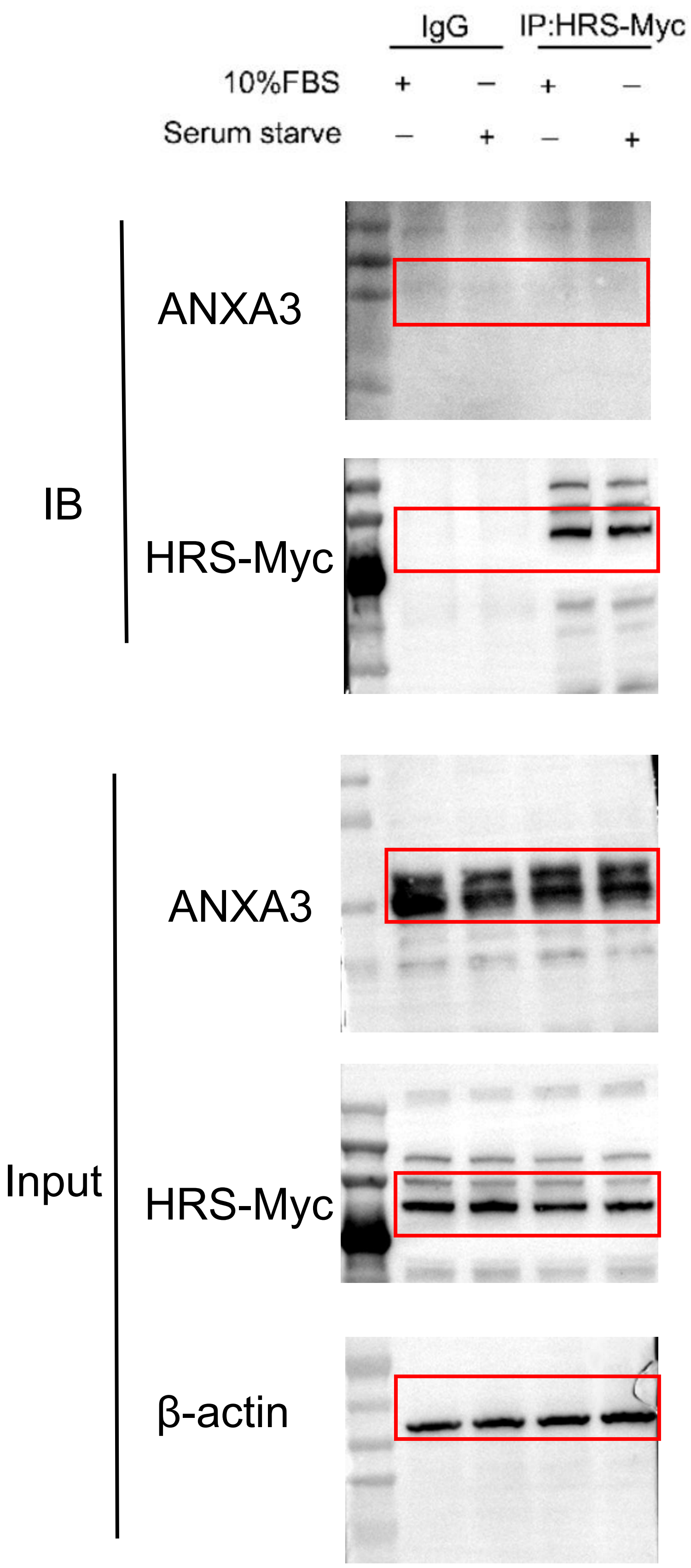

Hela

Figure S6A

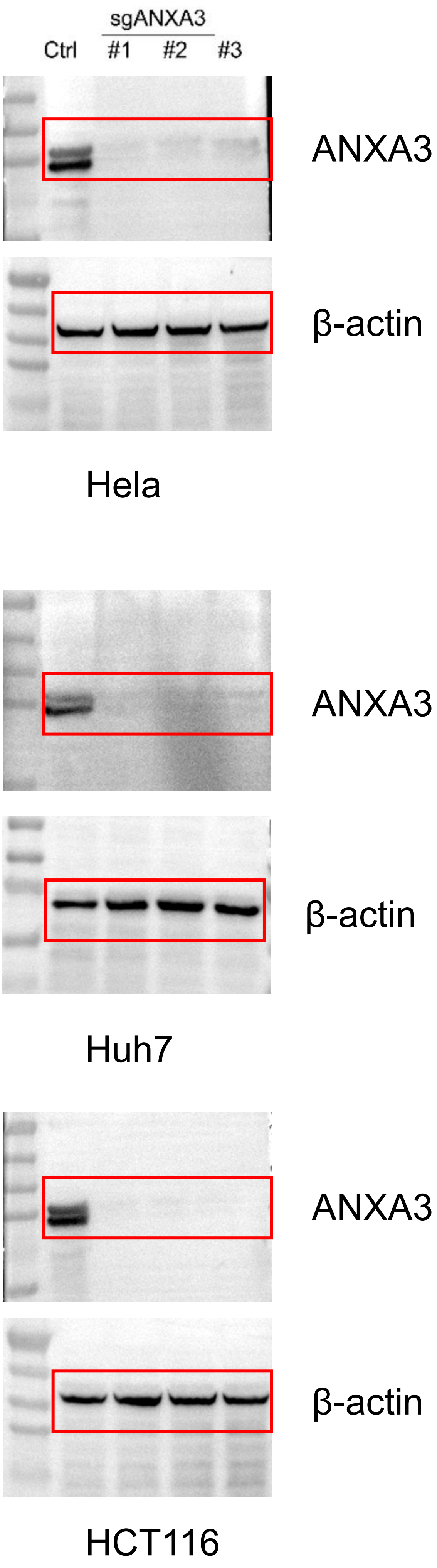
sgANXA3
Ctrl
#1
#2
#3
ANXA3
β-actin
Hela
ANXA3
β-actin
Huh7
ANXA3
β-actin
HCT116

Figure S6C

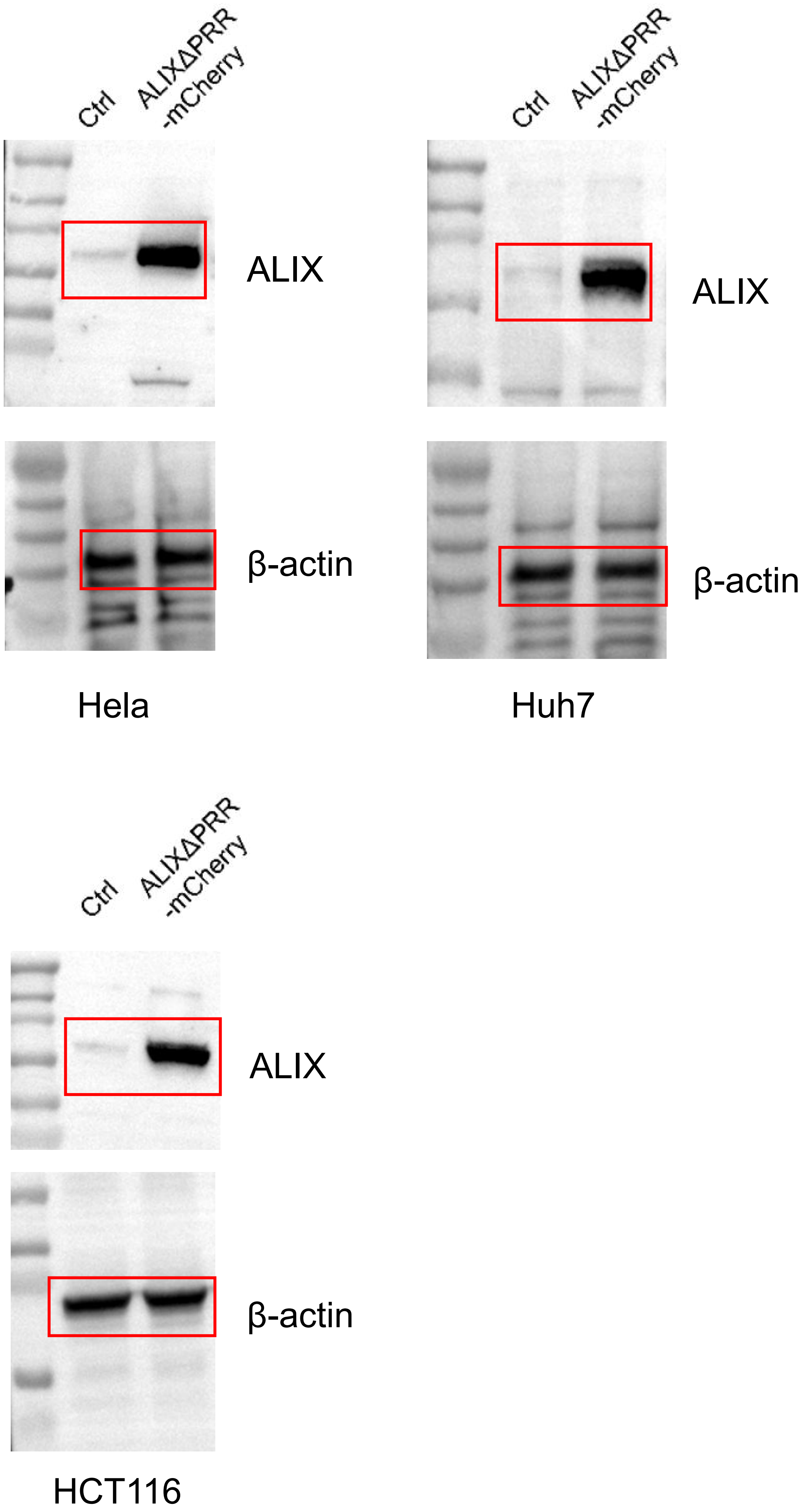
Ctrl
ALIXΔPRR
-mCherry
ALIX
β-actin
Hela
Ctrl
ALIXΔPRR
-mCherry
ALIX
β-actin
Huh7
Ctrl
ALIXΔPRR
-mCherry
ALIX
β-actin
HCT116

Figure S7A

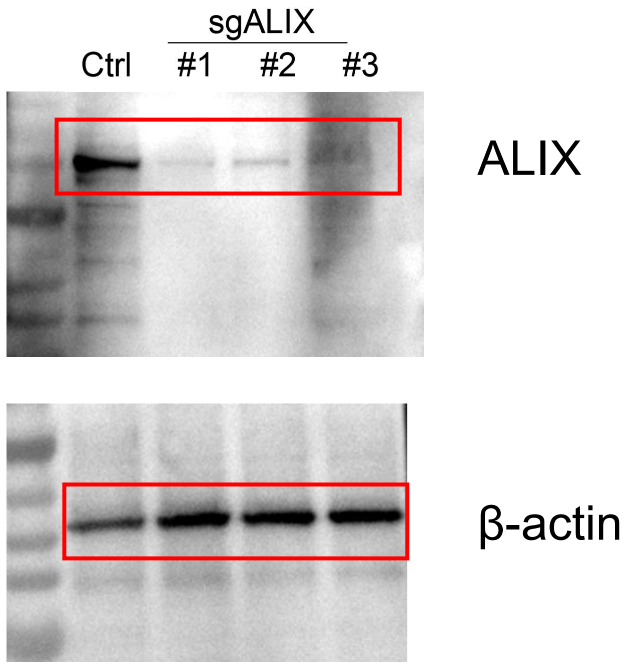

Supplement: Unedited blot and gel images [file jciinsight-11-197924-s272.pdf]
